# Synthesis and Transformation of Tricyclic KYNA Derivatives

**DOI:** 10.3390/ijms26136248

**Published:** 2025-06-28

**Authors:** Julián Robin Sárik, István Szatmári, Bálint Lőrinczi

**Affiliations:** 1 Institute of Pharmaceutical Chemistry, University of Szeged, Eotvos u. 6, H-6720 Szeged, Hungary; pomogacs98@gmail.com; 2 HUN-REN SZTE Stereochemistry Research Group, University of Szeged, Eötvös u. 6, H-6720 Szeged, Hungary

**Keywords:** KYNA, Mannich-reaction, Conrad–Limpach synthesis, tricyclic KYNA derivatives

## Abstract

Kynurenic acid (KYNA) derivatives condensed with an aromatic ring (tricyclic KYNA derivatives) have been successfully synthesized, and the reactivity of these analogues has been investigated in the modified Mannich reaction resulting in new Mannich bases. The *N,N*-dimethyl-ethylenediamine analogues of the tricyclic KYNA derivatives have also been successfully synthesized, and their reactivity in the modified Mannich reaction was investigated. The synthesized ring systems bear resemblance to molecules previously investigated as G-quadruplex binding agents. Based on this similarity, the synthesized tricyclic KYNA derivatives could be investigated as potential antiviral and anticancer molecules.

## 1. Introduction

KYNA, regarding its function, plays a prominent role in the human body. It is formed during the metabolism of tryptophan along with other endogenous mediators [1,2]. One of the most notable functions of KYNA is its antagonist effect on NMDA and AMPA glutamate receptors [3,4]. Due to its neuroprotective effect, deviation in KYNA levels can lead to neurodegenerative diseases [5,6,7,8,9,10,11]. Because of this and its sub-par blood–brain barrier (BBB) penetration, it is advantageous to synthesize new derivatives, which may grant specific biological functions and improve BBB penetration. During previous research, different KYNA amide derivatives were synthesized, which show prominent biological activity due to the introduction of a tertiary amine function into the amide side chain (**I** and **II**) [12,13,14,15]. This cationic function can also be formed using the modified Mannich reaction (mMr); thus its extension to the synthesis of different KYNA amides was carried out. These derivatives, due to their tertiary nitrogen atom bearing a new functional group from mMr reaction, were able to significantly increase BBB penetration [16,17].

**Figure 1:**
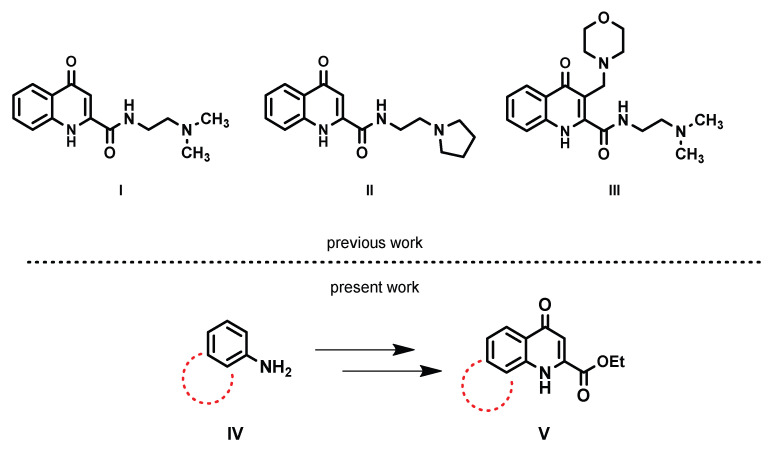
Comparison of previously synthesized KYNA amide derivatives and tricyclic KYNA derivatives.

From the previously synthesized Mannich bases of KYNA amides, compound **III** showed the highest BBB penetration Figure 1. The morpholinomethyl group exhibited outstanding penetration compared to other derivatives, indicating a possible structure–activity relationship [18,19]. Considering the positive effect of mMr, BBB penetration can further be enhanced by increasing the lipophilicity of the ligand. Of numerous options, the extension of the aromatic ring system is a possibility to achieve this desired property. Knowing this, aniline derivatives condensed with another aromatic ring were chosen as substrates for further investigation.

Besides this utilization, these molecules bear resemblance to structures present in ligands with capabilities of binding specific nucleic acids and forming metal complexes. Different transition metal and lanthanide complexes were observed as luminescent molecules [20,21,22]. These derivatives can be utilized in the detection of DNA fragments. Furthermore, this potent complex-forming and π-π stacking property can be used in the synthesis of G-quadruplex binding agents, as they can coordinate to the cations (typically Na^+^ and K^+^) that stabilize these systems. G-quadruplex nucleic acid structures are formed in guanine-rich sequences, and they are present in DNA and RNA as well. G-quadruplex structures were first discovered in the telomere regions, but since then their presence has also been observed in numerous human genome regions [23,24]. However, the property of DNA and RNA to form G-quadruplex cannot be regarded as a human-specific phenomenon. Recently, the presence of these structures has been investigated in viral nucleic acid, e.g., in HIV and West Nile virus as well [25,26]. Molecules binding to these structures were the candidates for research projects aimed at the synthesis of potential antitumor and antiviral derivatives [27,28,29]. Further increasing the potential of the structures described herein, different benzo[h]quinoline- and 1,10-phenanthroline-carboxamide derivatives were investigated in the literature as potential G-quadruplex binding molecules showing promising results in specific oligonucleotides [30]. These molecules can be regarded as potential lead structures to further expand our chemotherapeutic and antiviral molecule libraries.

## 2. Results and Discussion

**Scheme 1:**
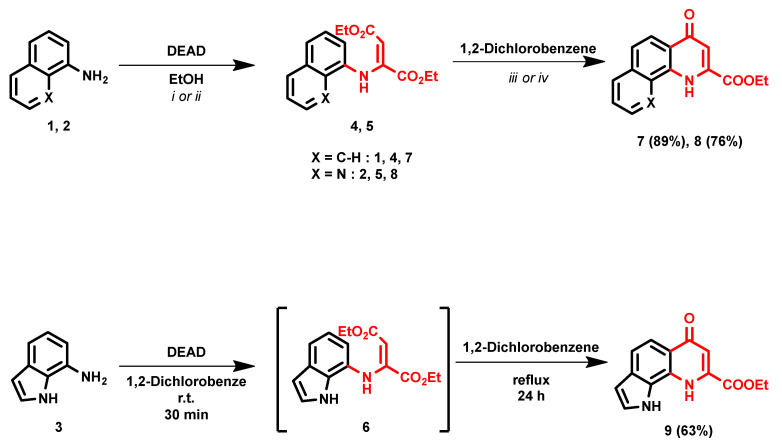
Synthesis of tricyclic KYNA derivatives; *i*: reflux, 2 h; *ii*: r.t., 2 h; *iii*: reflux, 12 h; *iv*: 200 °C, M.W., 2 h.

During the synthesis of the tricyclic KYNA derivatives, notable differences were observed in the enamine formation step. Compound **4** (and the end product **7**) have been synthesized using the Conrad–Limpach reaction in the literature [30]. However, a previously optimized method utilizing milder conditions [18] was applied with satisfactory yields (Scheme 1). Starting materials **2** and **3** showed higher reactivity than **1** as the formation of compounds **5** and **6** took place at room temperature (r.t.) with the same reaction time used for **4**. Furthermore, in the case of **6**, the formation of a side product was observed at higher temperatures; thus, for the synthesis of **6**, the temperature had to be strictly limited to r.t. The next step, the ring closure reaction, was carried out at different temperatures in a microwave reactor using 1,2-dichlorobenzene (DCB). A temperature of 180 °C was found to be inefficient in the case of compound **8**. The conversion reached a plateau at 200 °C (Table 1, entries 3–5), while further increasing the temperature had no notable effect on the reaction time. De-esterification and the following decarboxylation of **8** were observed at 230 °C (Table 1, Entry 6). The synthesis of compound **9** utilized reaction conditions similar to those applied for compound **7**; however, it included a crucial optimization step. Namely, the solution of the enamine was added dropwise to the preheated solvent, as higher enamine concentration led to the formation of a multicomponent reaction mixture and thus hindering the reaction and requiring further isolation of the desired product.

**Scheme 2:**
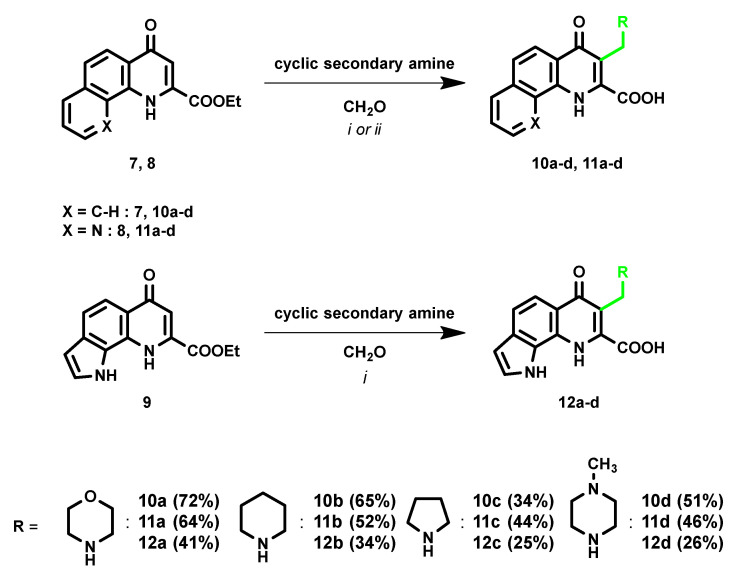
Investigation of mMr on tricyclic KYNA esters; *i*: 1,4-Dioxane, reflux, 12 h; *ii*: THF, 110 °C, M.W., 4 h.

As mentioned in the introduction, the mMr process is a possible way to introduce a tertiary nitrogen moiety, which shows causality with increased BBB penetration in the case of previous KYNA derivatives. Accordingly, the extension of mMr to the tricyclic KYNA derivatives was carried out. Paraformaldehyde was applied as a representative aldehyde. During the structure–activity analysis, the morpholinomethyl derivative gave the best BBB penetration increase. Thus, morpholine was used as the representative cyclic secondary amine for the optimization reactions [18,19]. Starting from compound **7** and **9**, the above-mentioned optimized method was applied with longer reaction times (Scheme 2). Compound **8** showed lower reactivity in 1,4-dioxane compared to other tricyclic KYNA derivatives; thus further optimization was necessary. Different solvents (DCM, EtOH, and THF) were tested in a microwave reactor at 110 °C. Among the solvents applied, no noticeable differences could be observed in the crude NMR spectra. Despite these results, THF was chosen as the optimal solvent as the desired product crystallized from the reaction mixture, and a more favorable work-up process could be applied.

The scopes and limitations of the reaction were investigated using different cyclic secondary amines (e.g., piperidine, pyrrolidine, and N-methylpiperazine). Compared to morpholine, other cyclic secondary amines showed similar reactivity in the optimized reaction. However, the work-up process was hindered by the presence of stronger bases and lower yields could be realized (Scheme 2).

On the basis of the reaction times, it can be surmised that the tricyclic KYNA derivatives have lower reactivity in comparison to other KYNA esters, synthesized previously. The introduction of a new aromatic ring onto the KYNA structure presumably creates a greater room for electron density to be further distributed and overall presents unfavorable conditions for mMr. Introducing a heteroatom in the C-ring will further decrease the electron density in the aromatic system.

As mentioned previously in the introduction, KYNA amides **I** and **II** were proven to have significant biological activity, with the *N,N*-dimethylethylenediamine derivative showing the highest increase in BBB penetration. Consequently, the synthesis of the corresponding tricyclic KYNA amide analogues was carried out.

**Scheme 3:**
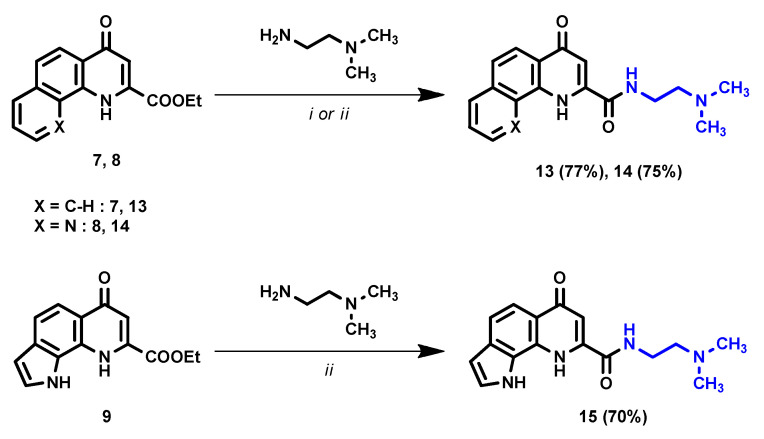
Synthesis of *N,N*-dimethylethylenediamine amide derivative. *i*: EtOH, 120 °C, M.W., 2 h; *ii*: neat, r.t., 2 h.

To achieve this goal, direct amidation was utilized. However, in the case of these tricyclic KYNA derivatives, the use of EtOH at reflux temperature was not sufficient (Scheme 3). For the synthesis of compound **13**, increasing the temperature to 120 °C in microwave conditions led to satisfactory conversions and yields. In the case of compounds **14** and **15**, the reaction was carried out in neat conditions due to more favorable reaction times and a simpler work-up process. The acidic N–H moiety in **9** presumably interfered with the amidation, as further purification had to be implemented in the work-up process to isolate **15**. Note, however, that purification was not necessary in the case of **13** and **14**.

**Scheme 4:**
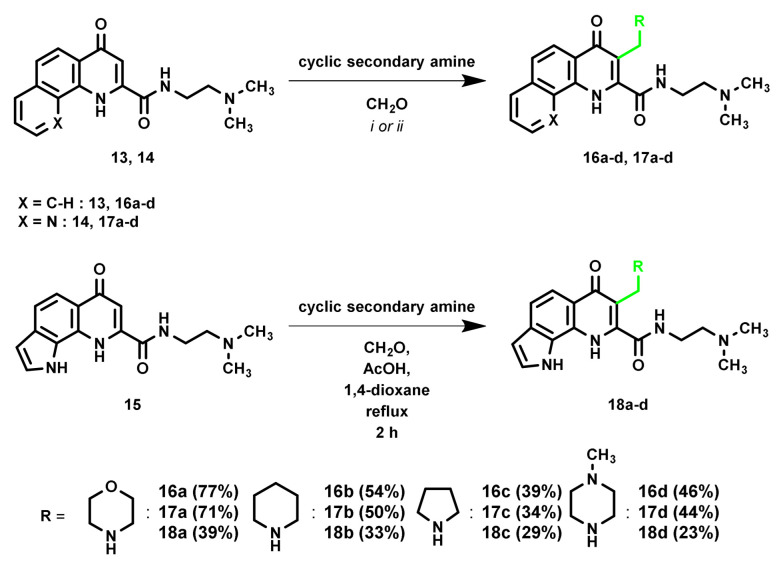
Investigation of mMr on tricyclic KYNA amides. *i*: 1,4-Dioxane, reflux, 8 h; *ii*: THF, 110 °C, M.W., 4 h.

To further investigate the reactivity of the tricyclic KYNA derivatives in the mMr, the aminoalkylation reaction was also tested on the amide analogues. As representative reagents, the aldehyde paraformaldehyde, while morpholine, a cyclic secondary amine was chosen. The reaction conditions, optimized for the ester derivative were applied for these test reactions (Scheme 4). In the case of **13** and **14**, the transformations took place in a satisfactory manner. In the case of **15**, in turn, using the same reaction conditions, a multicomponent reaction mixture was formed, and the desired product could not be isolated. However, despite testing other solvents (THF, DCM, and EtOH), no improvements could be achieved. As a further option, acidic (acetic acid) and basic (Et_3_N) additives were investigated. The application of acetic acid proved to be beneficial as the formation of the side products was hindered; thus the Mannich-base could be isolated.

Subsequently, the mMr was extended to cyclic secondary amines used previously. The results observed for these reactions bear resemblance to the ester derivative in terms of reactivity. Based on these results, it can be surmised that changing the ester to an amide side chain has no noticeable impact on the reactivity of the tricyclic KYNA derivatives. The only exception was compound **15**, where the pyrrole ring seemingly has a further impact on the reaction. The usage of an acidic additive was a necessity for the synthesis of the aminoalkyl derivatives.

## 3. Materials and Methods

^1^H and ^13^C-NMR spectra were recorded in DMSO-d_6_, CDCl_3_ and MeOH-d_4_ solutions in 5 mm tubes at room temperature (r.t.) on a Bruker Ascend 500 spectrometer with a 5 mm BBO Prodigy Probe or on a Bruker AVANCE III 600 MHz spectrometer equipped with a 5 mm CP-TCI triple-resonance cryoprobe (Bruker Biospin, Karlsruhe, Baden Württemberg, Germany) at 500 (1H) and 125 (13C) MHz or 600 (1H) and 150 (13C) MHz, with the deuterium signal of the solvent as the lock and TMS as internal standard (1H, 13C).

Melting points were determined on a Hinotek X-4 melting point apparatus. Merck Kieselgel 60F_254_ and Merck Aluminium Oxide 60F_254_ plates were used for TLC.

Reactions were conducted in a CEM Focused Microwave Synthesis System, Discover SP (CEM, Charlotte, NC, USA).

The flow injection analysis was performed with Thermo Scientific Orbitrap Exploris 240 hybrid quadrupole-Orbitrap (Thermo Fischer Scientific, Waltham, MA, USA) mass spectrometer coupled to a Waters Acquity I-Class UPLCTM (Waters, Manchester, UK).

### 3.1. Synthesis

#### 3.1.1. Synthesis of Tricyclic KYNA Derivatives

Method I: Naphtalen-1-amine (**1**) (0.9995 g, 6.98 mmol) was added to a 50 mL round-bottom flask and dissolved in 10 mL ethanol. Diethyl-acetylene dicarboxylate (1.426 g, 8.38 mmol) was added, and the solution was treated under reflux conditions for 2 h. After completion of the reaction, the solvent was evaporated under vacuum, and the residue was purified using column chromatography (SiO_2_, n-Hexane–EtOAc 4:1, R_f_ = 0.5). After purification, a yellow viscous liquid was acquired which was dissolved in 20 mL DCB. The solution was heated under reflux conditions for 12 h. The solvent was evaporated under vacuum, and the desired product was crystallized using EtOAc (10 mL) and washed with EtOAc (2 × 5 mL).

Method II: 8-Aminoquinoline (**2**) (1.006 g, 6.98 mmol) was added to a 50 mL round-bottom flask and dissolved in 10 mL ethanol. Diethyl-acetylene dicarboxylate (1.426 g, 8.38 mmol) was added, and the solution was stirred at r.t. for 2 h. After completion of the reaction, the solvent was evaporated under vacuum, and the residue was purified using column chromatography (Al_2_O_3_, n-Hexane–EtOAc 4:1, R_f_ = 0.5). After purification, a yellow viscous liquid was acquired which was dissolved in 5 mL DCB and put in a pressure-resistant 10 mL vessel. The reaction was heated in microwave reactor at 200 °C for 2 h. The solvent was evaporated under vacuum, and the residue was purified using column chromatography (Al_2_O_3_, DCM–EtOAc 1:1, R_f_ = 0.6). The desired product was crystallized using Et_2_O (10 mL).

Method III: 7-Amino-1*H*-indole (**3**) (0.9225 g, 6.98 mmol) was added to a 50 mL round-bottom flask and dissolved in 10 mL DCB. Diethyl-acetylene dicarboxylate (1.426 g, 8.38 mmol) was added, and the solution was stirred at r.t. for 30 min. After completion of the reaction, the solution was added dropwise to 20 mL preheated (180 °C) DCB over the course of 6 h. The mixture was refluxed for an additional 18 h. After competition of the reaction, a brown precipitate formed, which was filtered and washed with Et_2_O (3 × 10 mL).

**Diethyl 2-(naphthalen-1-ylamino)fumarate (4)**

Yield: 1.86 g, 85%. Mp.: yellow viscous liquid at r.t. HRMS calcd for [M + H^+^] m/z = 314.1387, found m/z = 314.1383; ^1^H NMR (CDCl_3_): 0.88 (3H, t, *J* = 7.04 Hz); 1.33 (3H, t, *J* = 7.04 Hz); 4.02 (2H, q, *J* = 7.18 Hz); 4.24 (2H, q, *J* = 7.18 Hz); 5.51 (1H, s); 6.97 (1H, d, *J* = 7.42 Hz); 7.34 (1H, t, *J* = 7.71 Hz); 7.49–7.57 (2H, m); 7.65 (1H, d, *J* = 8.14 Hz); 7.85 (1H, d, *J* = 7.83 Hz); 8.15 (1H, d, *J* = 8.02 Hz); 10.04 (1H, brs); ^13^C NMR (CDCl_3_): 13.5; 14.4; 60.0; 61.8; 93.9; 118.7; 122.2; 125.3; 125.4; 126.4; 126.5; 128.2; 128.3; 134.2; 136.8; 149.9; 164.3; 170.0 (Figures S1, S2 and S77)

**Diethyl 2-(quinolin-8-ylamino)fumarate (5)**

Yield: 1.67 g, 76%. Mp.: yellow viscous liquid at r.t. HRMS calcd for [M + H^+^] m/z = 315.1339, found m/z = 315.1335; ^1^H NMR (CDCl_3_): 1.15 (3H, t, *J* = 6.85 Hz); 1.32 (3H, t, *J* = 6.85 Hz); 4.20–4.30 (4H, m); 5.55 (1H, s); 6.98 (1H, d, *J* = 7.32 Hz); 7.36–7.46 (3H, m); 8.12 (1H, d, *J* = 8.14 Hz); 8.91 (1H, d, *J* = 4.07 Hz); 10.91 (1H, brs); ^13^C NMR (CDCl_3_): 13.8; 14.4; 60.0; 62.0; 96.3; 114.8; 121.4; 121.7; 126.3; 128.7; 136.0; 137.1; 140.0; 146.5; 149.0; 164.8; 168.9 (Figures S3, S4 and S78)

**Ethyl 4-oxo-1,4-dihydrobenzo[*h*]quinoline-2-carboxylate (7)**

Yield: 1.66 g, 89%. Mp.: 258.1–259.3 °C. HRMS calcd for [M + H^+^] m/z = 268.0968, found m/z = 268.0966; ^1^H NMR (CDCl_3_): 1.48 (3H, t, *J* = 7.04 Hz); 4.54 (2H, q, *J* = 7.14 Hz); 7.26 (1H, s); 7.74 (3H, t, *J* = 8.21 Hz); 7.99 (1H, t, *J* = 4.54 Hz); 8.23 (1H, t, *J* = 5.21 Hz); 8.33 (1H, d, *J* = 8.62 Hz); 9.80 (1H, brs); ^13^C NMR (CDCl_3_): 14.1; 63.5; 113.8; 120.0; 122.2; 122.9; 123.8; 125.1; 127.2; 129.1; 129.4; 135.2; 135.3; 136.1; 163.2; 179.1 (Figures S5, S6 and S79)

**Ethyl 4-oxo-1,4-dihydro-1,10-phenanthroline-2-carboxylate (8)**

Yield: 1.41 g, 76%. Mp.: decomposition over 150 °C. HRMS calcd for [M + H^+^] m/z = 269.0921, found m/z = 269.0918; ^1^H NMR (CDCl_3_): 1.49 (3H, t, *J* = 7.42 Hz); 4.55 (2H, q, *J* = 7.14 Hz); 7.22 (1H, s); 7.64–7.70 (2H, m); 8.27 (1H, d, *J* = 8.14 Hz); 8.36 (1H, d, *J* = 8.78 Hz); 8.98 (1H, d, *J* = 4.73 Hz); 11.00 (1H, brs); ^13^C NMR (CDCl_3_): 14.1; 63.2; 114.6; 119.2; 122.8; 123.0; 124.1; 125.1; 129.6; 135.9; 136.3; 137.0; 149.3; 162.5; 179.3 (Figures S7, S8 and S80)

**Ethyl 6-oxo-6,9-dihydro-1*H*-pyrrolo[3,2-*h*]quinoline-8-carboxylate (9)**

Yield: 1.13 g, 63%. Mp.: decomposition over 270 °C. HRMS calcd for [M + H^+^] m/z = 257.0921, found m/z = 257.0917; ^1^H NMR (DMSO-d_6_): 1.41 (3H, t, *J* = 6.97 Hz); 4.50 (2H, q, *J* = 7.10 Hz); 6.73–6.77 (1H, m); 7.29 (1H, s); 7.69–7.73 (1H, m); 7.79–7.83 (2H, m); 12.40 (1H, brs); ^13^C NMR (DMSO-d_6_): 14.5; 63.2; 104.2; 107.6; 112.2; 114.4; 114.5; 116.8; 118.8; 121.7; 125.3; 128.2; 130.7; 162.6 (Figures S9, S10 and S81)

#### 3.1.2. Synthesis of Mannich-Bases from Ester Derivatives

Method I: Ester derivative (**7** to achieve **10a**–**d** and **9** to achieve **12a**–**d**) (0.187 mmol), paraformaldehyde (6.7 mg, 0.224 mmol), and the corresponding cyclic secondary amine (morpholine in case of a, piperidine in case of b, pyrrolidine in case of c, and *N*-methylpiperazine in case of d) (0.224 mmol) were added to a round-bottom flask. The mixture was heated under reflux conditions in 1,4-dioxane (10 mL) for 12 h. After completion of the reaction, the solvent was removed under reduced pressure, and the desired product was crystallized using EtOAc (5 mL), filtered, and washed with 2 × 5 mL EtOAc.

Method II: Ester derivative (**8**) (50.2 mg, 0.187 mmol), paraformaldehyde (6.7 mg, 0.224 mmol), and the corresponding cyclic secondary amine (morpholine in case of **a**, piperidine in case of **b**, pyrrolidine in case of **c**, and *N*-methylpiperazine in case of **d**) (0.224 mmol) were added to a 10 mL pressure resistant vessel. The mixture was heated in microwave reactor in THF (10 mL) at 110 °C for 4 h. After completion of the reaction, the desired product crystallized from the solvent and the mixture was filtered and washed with Et_2_O (5 mL).

**3-(Morpholinomethyl)-4-oxo-1,4-dihydrobenzo[*h*]quinoline-2-carboxylic acid (10a)**

Yield: 45.6 mg, 72%. Mp.: 200.0–201.0 °C. HRMS calcd for [M + H^+^] m/z = 339.1339, found m/z = 339.1335; ^1^H NMR (CDCl_3_): 3.07–3.16 (2H, m); 3.40–3.47 (2H, m); 3.83–3.91 (2H, m); 4.06–4.12 (2H, m); 4.63 (2H, s); 7.63 (1H, t, *J* = 7.25 Hz); 7.69 (1H, t, *J* = 7.62 Hz); 7.75 (1H, d, *J* = 8.14 Hz); 7.94 (1H, d, *J* = 8.14 Hz); 8.24 (1H, d, *J* = 7.87 Hz); 8.27 (1H, d, *J* = 8.41 Hz); 10.95 (1H, brs); 14.49 (1H, brs); ^13^C NMR (CDCl_3_): 50.7; 51.3; 64.4; 110.7; 120.5; 121.8; 122.1; 122.9; 125.5; 127.3; 129.1; 129.2; 135.1; 135.3; 142.9; 165.3; 178.0 (Figures S11, S12 and S82)

**4-Oxo-3-(piperidin-1-ylmethyl)-1,4-dihydrobenzo[*h*]quinoline-2-carboxylic acid (10b)**

Yield: 40.9 mg, 65%. Mp.: 258.2–259.1 °C. HRMS calcd for [M + H^+^] m/z = 337.1547, found m/z = 337.1544; ^1^H NMR (MeOH-d_4_): 1.56–1.69 (1H, m); 1.71–1.90 (3H, m); 1.94–2.08 (2H, m); 3.06–3.20 (2H, m); 3.49–3.62 (2H, m); 4.67 (2H, s); 7.77–7.84 (3H, m); 8.01–8.06 (1H, m); 8.18 (1H, d, *J* = 7.87 Hz); 8.55–8.60 (1H, m); ^13^C NMR (MeOH-d_4_): 21.5; 23.0; 51.0; 52.1; 111.5; 120.9; 121.0; 121.4; 123.0; 125.4; 127.3; 128.7; 129.2; 135.2; 165.3; 178.4 (Figures S13, S14 and S83)

**4-Oxo-3-(pyrrolidin-1-ylmethyl)-1,4-dihydrobenzo[*h*]quinoline-2-carboxylic acid **(10c)****

Yield: 20.5 mg, 34%. Mp.: 252.4–253.6 °C. HRMS calcd for [M + H^+^] m/z = 323.1390, found m/z = 323.1386; ^1^H NMR (CDCl_3_): 2.11–2.29 (4H, m); 3.14–3.25 (2H, m); 3.69–3.79 (2H, m); 4.72–4.79 (2H, m); 7.62 (1H, t, *J* = 8.67 Hz); 7.68 (1H, t, *J* = 8.34 Hz); 7.73 (1H, d, *J* = 8.24 Hz); 7.93 (1H, d, *J* = 8.01 Hz); 8.24–8.30 (2H, m); 11.06 (1H, brs); 12.57 (1H, brs); ^13^C NMR (CDCl_3_): 23.7; 49.5; 52.8; 112.3; 120.7; 122.0; 122.2; 123.1; 125.1; 127.1; 129.0; 135.1; 135.2; 143.2; 165.1; 178.3 (Figures S15, S16 and S84)

**3-((4-Methylpiperazin-1-yl)methyl)-4-oxo-1,4-dihydrobenzo[*h*]quinoline-2-carboxylic acid (10d)**

Yield: 33.5 mg, 51%. Mp.: 141.3–142.8 °C. HRMS calcd for [M + H^+^] m/z = 352.1656, found m/z = 352.1651; 1H NMR (MeOH-d_4_): 2.32–2.56 (5H, m); 2.95–3.12 (2H, m); 3.21–3.35 (2H, m); 3.48–3.62 (2H, m); 4.69 (2H, s); 7.72–7.82 (3H, m); 7.97–8.03 (1H, m); 8.14 (1H, t, J = 8.87 Hz); 8.47–8.55 (1H, m); ^13^C NMR (MeOH-d_4_): 44.1; 50.6; 50.9; 51.6; 120.8; 120.9; 121.4; 122.9; 125.4; 127.3; 128.7; 129.1; 135.1; 135.5; 178.4 (Figures S17, S18 and S85)

**3-(Morpholinomethyl)-4-oxo-1,4-dihydro-1,10-phenanthroline-2-carboxylic acid (11a)**

Yield: 40.6 mg, 64%. Mp.: decomposition over 250 °C. HRMS calcd for [M + H^+^] m/z = 340.1292, found m/z = 340.1287; ^1^H NMR (CDCl_3_): 3.06–3.16 (2H, m); 3.73 (2H, d, *J* = 12.30 Hz); 3.91 (2H, t, *J* = 12.23 Hz); 4.09 (2H, d, *J* = 12.79 Hz); 4.64 (2H, s); 7.56–7.60 (1H, m); 7.64 (1H, d, *J* = 9.04 Hz); 8.19 (1H, d, *J* = 8.15 Hz); 8.27 (1H, d, *J* = 8.65 Hz); 11.64 (1H, brs); 14.64 (1H, brs); ^13^C NMR (CDCl_3_): 50.8; 51.3; 64.4; 111.7; 122.9; 123.1; 123.2; 124.1; 129.4; 135.9; 139.1; 144.0; 149.4; 165.0; 178.0 (Figures S19, S20 and S86)

**4-Oxo-3-(piperidin-1-ylmethyl)-1,4-dihydro-1,10-phenanthroline-2-carboxylic acid (11b)**

Yield: 32.8 mg, 52%. Mp.: decomposition over 265 °C. HRMS calcd for [M + H^+^] m/z = 338.1499, found m/z = 338.1498; ^1^H NMR (CDCl_3_): 1.63–1.71 (2H, m); 1.77–1.89 (2H, m); 1.94–2.02 (2H, m); 2.88 (2H, q, *J* = 10.92 Hz); 3.51 (2H, d, *J* = 12.37 Hz); 4.54 (2H, s); 7.61–7.66 (1H, m); 7.68 (1H, d, *J* = 8.84 Hz); 8.24 (1H, d, *J* = 8.17 Hz); 8.30 (1H, d, *J* = 8.17 Hz); 8.97 (1H, d, *J* = 4.40 Hz); 11.79 (1H, brs); 13.10 (1H, brs); ^13^C NMR (CDCl_3_): 22.4; 44.5; 51.0; 51.6; 111.9; 122.9; 123.1; 123.2; 124.1; 129.5; 136.0; 144.6; 149.6; 165.0; 178.1 (Figures S21, S22 and S87)

**4-Oxo-3-(pyrrolidin-1-ylmethyl)-1,4-dihydro-1,10-phenanthroline-2-carboxylic acid (11c)**

Yield: 26.6 mg, 44%. Mp.: decomposition over 250 °C. HRMS calcd for [M + H^+^] m/z = 324.1343, found m/z = 324.1339; ^1^H NMR (DMSO-d_6_): 1.86–1.99 (2H, m); 1.99–2.12 (2H, m); 3.10–3.22 (2H, m); 3.40–3.52 (2H, m); 4.65 (2H, s); 7.84–7.88 (2H, m); 8.18 (1H, d, *J* = 9.14 Hz); 8.57 (1H, d, *J* = 8.33 Hz); 9.10 (1H, d, *J* = 5.13 Hz); 10.84 (1H, brs); 11.66 (1H, brs); ^13^C NMR (DMSO-d_6_): 23.4; 49.2; 53.0; 113.8; 122.8; 122.9; 123.3; 125.0; 129.6; 135.3; 137.3; 139.0; 144.9; 150.6; 163.1; 177.7 (Figures S23, S24 and S88)

**3-((4-Methylpiperazin-1-yl)methyl)-4-oxo-1,4-dihydro-1,10-phenanthroline-2-carboxylic acid (11d)**

Yield: 30.3 mg, 46%. Mp.: decomposition over 255 °C. HRMS calcd for [M + H^+^] m/z = 353.1608, found m/z = 353.1604; 1H NMR (DMSO-d6): 2.16–2.29 (5H, m); 2.83–2.94 (2H, m); 3.04–3.14 (2H, m); 3.33–3.43 (2H, m); 4.55 (2H, s); 7.84–7.89 (2H, m); 8.17 (1H, d, *J* = 8.91 Hz); 8.58 (1H, d, *J* = 8.27 Hz); 9.11 (1H, d, *J* = 4.33 Hz); 11.57 (1H, brs); 12.15 (1H, brs); ^13^C NMR (DMSO-d_6_): 45.5; 49.7; 50.2; 52.2; 64.2; 112.8; 122.7; 122.9; 123.4; 125.1; 129.6; 135.4; 137.3; 138.9; 144.8; 150.6; 163.8; 177.6 (Figures S25, S26 and S89)

**7-(Morpholinomethyl)-6-oxo-6,9-dihydro-1*H*-pyrrolo[3,2-*h*]quinoline-8-carboxylic acid (12a)**

Yield: 25.1 mg, 41%. Mp.: decomposition over 230 °C. HRMS calcd for [M + H^+^] m/z = 328.1292, found m/z = 328.1290; ^1^H NMR (DMSO-d_6_): 3.02–3.32 (4H, m); 3.54–4.06 (4H, m); 4.51 (2H, s); 6.61–6.65 (1H, m); 7.54 (1H, d, *J* = 8.57 Hz); 7.58–7.62 (1H, m); 7.76 (1H, d, *J* = 8.57 Hz); 11.62 (1H, brs); 12.65 (1H, brs); ^13^C NMR (DMSO-d_6_): 50.2; 50.9; 64.2; 103.4; 109.0; 116.3; 118.1; 119.4; 124.2; 127.4; 127.7; 130.5; 146.3; 165.1; 177.4 (Figures S27–S29 and S90)

**6-oxo-7-(piperidin-1-ylmethyl)-6,9-dihydro-1*H*-pyrrolo[3,2-*h*]quinoline-8-carboxylic acid (12b)**

Yield: 20.7 mg, 34%. Mp.: decomposition over 230 °C. HRMS calcd for [M + H^+^] m/z = 326.1499, found m/z = 326.1495; ^1^H NMR (DMSO-d_6_): 1.42–1.72 (4H, m); 1.80–1.92 (2H, m); 2.89–3.01 (2H, m); 3.28–3.34 (2H, m); 4.41 (2H, s); 6.60–6.64 (1H, m); 7.53 (1H, d, *J* = 8.58 Hz); 7.57–7.61 (1H, m); 7.75 (1H, d, *J* = 8.58 Hz); 11.54 (1H, brs); 11.77 (1H, brs); 12.46 (1H, brs); ^13^C NMR (DMSO-d_6_): 22.1; 23.4; 50.5; 50.9; 103.4; 109.1; 116.4; 116.4; 118.0; 119.3; 124.2; 127.3; 127.7; 130.5; 146.5; 165.0; 177.4 (Figures S30–S32 and S91)

**6-oxo-7-(pyrrolidin-1-ylmethyl)-6,9-dihydro-1*H*-pyrrolo[3,2-*h*]quinoline-8-carboxylic acid (12c)**

Yield: 14.6 mg, 25%. Mp.: decomposition over 220 °C. HRMS calcd for [M + H^+^] m/z = 312.1343, found m/z = 312.1340; ^1^H NMR (DMSO-d_6_): 1.86–2.11 (4H, m); 3.11–3.22 (2H, m); 3.32–3.48 (2H, m); 4.57 (2H, s); 6.62–6.65 (1H, m); 7.54 (1H, d, *J* = 8.51 Hz); 7.59–7.62 (1H, m); 7.77 (1H, d, *J* = 8.52 Hz); 10.82 (1H, brs); 11.69 (1H, brs); 12.94 (1H, brs); ^13^C NMR (DMSO-d_6_): 23.4; 49.8; 52.7; 103.4; 110.4; 116.2; 118.1; 119.5; 124.3; 127.3; 127.7; 130.5; 146.0; 164.8; 177.6 (Figures S33, S34 and S92)

**7-((4-methylpiperazin-1-yl)methyl)-6-oxo-6,9-dihydro-1*H*-pyrrolo[3,2-*h*]quinoline-8-carboxylic acid (12d)**

Yield: 16.5 mg, 26%. Mp.: decomposition over 250 °C. HRMS calcd for [M + H^+^] m/z = 314.1608, found m/z = 314.1605; 1H NMR (DMSO-d6): 2.10–2.29 (5H, m); 2.73–2.94 (2H, m); 2.94–3.14 (2H, m); 3.21–3.31 (2H, m); 4.41 (2H, s); 6.60–6.65 (1H, m); 7.53 (1H, d, *J* = 8.35 Hz); 7.58–7.61 (1H, m); 7.76 (1H, d, *J* = 8.64 Hz); 11.56 (1H, brs); 12.41 (1H, brs); 12.53 (1H, brs); ^13^C NMR (DMSO-d_6_): 40.4; 45.5; 50.0; 103.4; 116.3; 117.9; 119.3; 124.2; 127.3; 127.7; 130.4; 146.4; 165.2; 177.4 (Figures S35–S37 and S93)

#### 3.1.3. Synthesis of KYNA Amide Derivatives

Method I: Ester derivative (**7**) (499.8 mg, 1.87 mmol) was added to a pressure resistant 10 mL vessel and was dissolved in 5 mL ethanol. *N,N*-dimethylethylenediamine (197.5 mg, 2.24 mmol) was added, and the solution was heated in microwave reactor at 120 °C for 2 h. After the completion of the reaction, the mixture was transferred to a 25 mL round-bottom flask, and the solvent was evaporated under vacuum. The desired product was crystallized using DCM (10 mL) and washed with DCM (2 × 5 mL).

Method II: Ester derivative (**8** to achieve **14**, or **9** to achieve **15**) (1.87 mmol) was added to an excess of *N,N*-dimethylethylenediamine (1 mL, 9.08 mmol) in a 10 mL pressure-resistant vessel and closed. The reaction was stirred at r.t. for 2 h. After the completion of the reaction, the mixture was transferred to a 25 mL round-bottom flask. In the case of compound 14, the desired product was crystallized using Et_2_O–EtOH (99:1) and washed with Et_2_O (2 × 5 mL). In the case of compound 15, the mixture was purified using column chromatography (SiO_2_, DCM–MeOH 1:1, R_f_ = 0.3). The desired product was crystallized using EtOAc (10 mL) and washed with EtOAc (2 × 5 mL).

***N*-(2-(dimethylamino)ethyl)-4-oxo-1,4-dihydrobenzo[*h*]quinoline-2-carboxamide (13)**

Yield: 445.5 mg, 77 %. Mp.: 252.7–253.8 °C. HRMS calcd for [M + H^+^] m/z = 310.1550, found m/z = 315.1547; ^1^H NMR (MeOH-d_4_): 2.49 (6H, s); 2.81 (2H, t, *J* = 7.14 Hz); 3.72 (2H, t, *J* = 7.47 Hz); 7.62 (1H, s); 7.69–7.75 (2H, m); 7.81 (1H, d, *J* = 7.87 Hz); 7.92–7.96 (1H, m); 8.15 (1H, d, *J* = 8.34 Hz); 9.31–9.36 (1H, m); ^13^C NMR (MeOH-d_4_): 36.5; 44.0; 58.0; 104.4; 119.3; 119.8; 124.04; 126.3; 126.4; 125.5; 127.5; 128.0; 130.6; 134.3; 145.8; 148.5; 165.2; 166.3 (Figures S38, S39 and S94)

***N*-(2-(dimethylamino)ethyl)-4-oxo-1,4-dihydro-1,10-phenanthroline-2-carboxamide (14)**

Yield: 435.3 mg, 75 %. Mp.: 256.0–256.7 °C. HRMS calcd for [M + H^+^] m/z = 311.1503, found m/z = 311.1499; ^1^H NMR (MeOH-d_4_): 2.52 (6H, s); 2.87 (2H, t, *J* = 6.79 Hz); 3.72 (2H, t, *J* = 6.49 Hz); 7.36 (1H, s); 7.76–7.82 (2H, m); 8.27 (1H, d, *J* = 8.98 Hz); 8.45 (1H, d, *J* = 8.17 Hz); 9.05 (1H, d, *J* = 4.20 Hz); ^13^C NMR (MeOH-d_4_): 36.7; 43.8; 57.6; 108.1; 121.6; 123.6; 123.7; 129.7; 136.7; 149.2; 154.9; 164.4; 176.9; 184.9 (Figures S40, S41 and S95)

***N*-(2-(dimethylamino)ethyl)-6-oxo-6,9-dihydro-1*H*-pyrrolo[3,2-*h*]quinoline-8-carboxamide (15)**

Yield: 390.5 mg, 70 %. Mp.: 163.0–163.8 °C. HRMS calcd for [M + H^+^] m/z = 299.1503, found m/z = 299.1497; ^1^H NMR (MeOH-d_4_): 2.41 (6H, s); 2.71 (2H, t, *J* = 6.53 Hz); 3.67 (2H, t, *J* = 6.51 Hz); 6.68 (1H, d, *J* = 2.69 Hz); 7.30 (1H, s); 7.48 (1H, d, *J* = 2.48 Hz); 7.71 (1H, d, *J* = 8.78 Hz); 7.83 (1H, d, *J* = 8.65 Hz); ^13^C NMR (MeOH-d_4_): 36.8; 44.1; 58.0; 102.5; 103.1; 106.7; 111.4; 113.4; 118.3; 120.1; 124.7; 128.8; 135.6; 146.1; 165.3; 188.8 (Figures S42–S45 and S96)

#### 3.1.4. Synthesis of Mannich-Bases from Amide Derivatives

Method I: Amide derivative (**13**) (50.1 mg, 0.162 mmol), paraformaldehyde (5.8 mg, 0.194 mmol), and the corresponding cyclic secondary amine (morpholine in case of **a**, piperidine in case of **b**, pyrrolidine in case of **c**, and *N*-methylpiperazine in case of **d**) (0.194 mmol) were added to a round-bottom flask. The mixture was treated under reflux conditions in 1,4-dioxane (10 mL) for 8 h. After completion of the reaction, the solvent was removed under reduced pressure, and the desired product was crystallized using EtOAc (5 mL), filtered, and washed with 2 × 5 mL EtOAc.

Method II: Amide derivative (**14**) (50.3 mg, 0.162 mmol), paraformaldehyde (5.8 mg, 0.194 mmol), and the corresponding cyclic secondary amine (morpholine in case of **a**, piperidine in case of **b**, pyrrolidine in case of **c**, and *N*-methylpiperazine in case of **d**) (0.194 mmol) were added to a pressure resistant 10 mL vessel. The mixture was treated in microwave reactor in THF (10 mL) at 110 °C for 4 h. After completion of the reaction, the desired product crystallized from the solvent and the mixture was filtered and washed with Et_2_O (5 mL).

Method III: Amide derivative (**15**) (48.3 mg, 0.162 mmol), paraformaldehyde (5.8 mg, 0.194 mmol), the corresponding cyclic secondary amine (morpholine in case of **a**, piperidine in case of **b**, pyrrolidine in case of **c**, and *N*-methylpiperazine in case of **d**) (0.194 mmol), and acetic acid (11.6 mg, 0.194 mmol) were added to a round-bottom flask. The mixture was treated under reflux conditions in 1,4-dioxane (10 mL) for 2 h. After completion of the reaction, the solvent was removed under reduced pressure, and the desired product was crystallized using Et_2_O (5 mL), filtered, and washed with 2 × 5 mL Et_2_O.

***N*-(2-(dimethylamino)ethyl)-3-(morpholinomethyl)-4-oxo-1,4-dihydrobenzo[*h*]quinoline-2-carboxamide (16a)**

Yield: 51.0 mg, 77 %. Mp.: 130.5–132.1 °C. HRMS calcd for [M + H^+^] m/z = 409.2234, found m/z = 409.2229; ^1^H NMR (MeOH-d_4_): 2.41 (6H, s); 2.70 (2H, t, *J* = 7.37 Hz); 2.90–2.97 (4H, m); 3.66 (2H, t, *J* = 7.34 Hz); 3.80–3.86 (4H, m); 4.45 (2H, s); 7.67–7.73 (2H, m); 7.77 (1H, d, *J* = 7.94 Hz); 7.91–7.97 (1H, m); 8.18 (1H, d, *J* = 8.01 Hz); 9.08–9.14 (1H, m); ^13^C NMR (MeOH-d_4_): 36.9; 44.2; 51.8; 53.9; 58.1; 65.5; 111.9; 120.4; 120.7; 123.5; 125.5; 126.3; 127.7; 128.0; 129.2; 134.5; 143.0; 146.8; 167.3; 171.5 (Figures S46, S47 and S97)

***N*-(2-(dimethylamino)ethyl)-4-oxo-3-(piperidin-1-ylmethyl)-1,4-dihydrobenzo[*h*]quinoline-2-carboxamide (16b)**

Yield: 35.6 mg, 54 %. Mp.: 112.3–113.6 °C. HRMS calcd for [M + H^+^] m/z = 407.2442, found m/z = 407.2438; ^1^H NMR (MeOH-d_4_): 1.60–2.00 (6H, m); 2.42 (6H, s); 2.73 (2H, t, *J* = 7.57 Hz); 3.02–3.38 (4H, m); 3.68 (2H, t, *J* = 7.91 Hz); 4.67 (2H, s); 7.62–7.68 (2H, m); 7.71 (1H, d, *J* = 8.34 Hz); 7.87–7.92 (1H, m); 8.24 (1H, d, *J* = 8.34 Hz); 9.25–9.31 (1H, m); ^13^C NMR (MeOH-d_4_): 21.8; 23.2; 36.7; 44.2; 52.0; 53.2; 58.2; 109.8; 121.3; 122.7; 124.4; 124.5; 125.7; 127.2; 127.3; 131.5; 134.5; 145.7; 147.9; 169.3; 173.9 (Figures S48, S49 and S98)

***N*-(2-(dimethylamino)ethyl)-4-oxo-3-(pyrrolidin-1-ylmethyl)-1,4-dihydrobenzo[*h*]quinoline-2-carboxamide (16c)**

Yield: 24.8 mg, 39 %. Mp.: 146.7–148.2 °C. HRMS calcd for [M + H^+^] m/z = 393.2285, found m/z = 393.2283; ^1^H NMR (MeOH-d_4_): 2.00–2.08 (4H, m); 2.31 (6H, s); 2.61 (2H, t, *J* = 7.71 Hz); 3.31–3.38 (4H, m); 3.56 (2H, t, *J* = 7.67 Hz); 4.71 (2H, s); 7.50–7.55 (2H, m); 7.58 (1H, d, *J* = 7.67 Hz); 7.74–7.79 (1H, m); 8.13 (1H, d, *J* = 8.27 Hz); 9.12–9.18 (1H, m); ^13^C NMR (MeOH-d_4_): 22.9; 36.7; 44.2; 51.4; 52.8; 58.2; 110.9; 121.4; 123.1; 124.3; 124.4; 125.6; 127.2; 127.3; 131.6; 134.5; 145.8; 147.7; 169.1; 174.1 (Figures S50, S51 and S99)

***N*-(2-(dimethylamino)ethyl)-3-((4-methylpiperazin-1-yl)methyl)-4-oxo-1,4-dihydrobenzo[*h*]quinoline-2-carboxamide (16d)**

Yield: 31.4 mg, 46 %. Mp.: 109.0–109.9 °C. HRMS calcd for [M + H^+^] m/z = 422.2551, found m/z = 422.2549; ^1^H NMR (MeOH-d_4_): 2.35 (3H, s); 2.41 (6H, s); 2.57–2.73 (4H, m); 2.70 (2H, t, *J* = 7.14 Hz); 2.99–3.13 (4H, m); 3.66 (2H, t, *J* = 6.31 Hz); 4.54 (2H, s); 7.65–7.72 (2H, m); 7.75 (1H, d, *J* = 8.98 Hz); 7.90–7.95 (1H, m); 8.18 (1H, d, *J* = 8.78 Hz); 9.12–9.18 (1H, m); ^13^C NMR (MeOH-d_4_): 36.9; 44.2; 44.3; 51.0; 53.0; 53.3; 58.1; 111.4; 120.5; 121.1; 123.8; 125.3; 126.2; 127.6; 127.8; 129.9; 134.5; 143.8; 147.1; 167.9; 171.8 (Figures S52, S53 and S100)

***N*-(2-(dimethylamino)ethyl)-3-(morpholinomethyl)-4-oxo-1,4-dihydro-1,10-phenanthroline-2-carboxamide (17a)**

Yield: 47.1 mg, 71 %. Mp.: 145.6–147.1 °C. HRMS calcd for [M + H^+^] m/z = 410.2187, found m/z = 410.2184; ^1^H NMR (CDCl_3_): 2.30 (6H, s); 2.59 (2H, t, *J* = 6.13 Hz); 2.68–2.74 (4H, m); 3.66 (2H, q, *J* = 5.95 Hz); 3.68–3.76 (4H, m); 3.88 (2H, s); 7.60–7.68 (2H, m); 8.24 (1H, d, *J* = 8.29 Hz); 8.33 (1H, d, *J* = 8.29 Hz); 8.96 (1H, d, *J* = 3.98 Hz); 11.39 (1H, brs); ^13^C NMR (CDCl_3_): 38.5; 45.5; 50.8; 51.9; 58.7; 66.9; 118.0; 122.7; 123.4; 124.0; 129.4; 136.1; 139.8; 141.2; 149.3; 163.2; 178.5 (Figures S54–S56 and S101)

***N*-(2-(dimethylamino)ethyl)-4-oxo-3-(piperidin-1-ylmethyl)-1,4-dihydro-1,10-phenanthroline-2-carboxamide (17b)**

Yield: 33.0 mg, 50 %. Mp.: 112.1–112.8 °C. HRMS calcd for [M + H^+^] m/z = 408.2394, found m/z = 408.2391; ^1^H NMR (MeOH-d_4_): 1.64–1.76 (2H, m); 1.80–1.92 (4H, m); 2.39 (6H, s); 2.73 (2H, t, *J* = 7.46 Hz); 3.12–3.30 (4H, m); 3.68 (2H, t, *J* = 7.46 Hz); 4.69 (2H, s); 7.70–7.76 (2H, m); 8.37 (1H, d, *J* = 8.37 Hz); 8.42 (1H, d, *J* = 8.37 Hz); 9.01 (1H, d, *J* = 4.33 Hz); ^13^C NMR (MeOH-d_4_): 22.0; 23.5; 36.8; 44.2; 52.1; 52.5; 58.2; 122.8; 122.9; 123.1; 125.2; 129.9; 136.9; 145.0; 148.4; 168.8; 175.1 (Figures S57–S59 and S102)

***N*-(2-(dimethylamino)ethyl)-4-oxo-3-(pyrrolidin-1-ylmethyl)-1,4-dihydro-1,10-phenanthroline-2-carboxamide (17c)**

Yield: 21.7 mg, 34 %. Mp.: 71.2–71.9 °C. HRMS calcd for [M + H^+^] m/z = 394.2238, found m/z = 394.2235; ^1^H NMR (CDCl_3_): 1.75–1.89 (4H, m); 2.30 (6H, s); 2.56 (2H, t, *J* = 6.13 Hz); 2.69–2.83 (4H, m); 3.64 (2H, t, *J* = 5.77 Hz); 4.01 (2H, s); 7.58–7.68 (2H, m); 8.24 (1H, d, *J* = 8.36 Hz); 8.35 (1H, d, *J* = 8.36 Hz); 8.96 (1H, d, *J* = 3.45 Hz); 11.35 (1H, brs); 12.13 (1H, brs); ^13^C NMR (CDCl_3_): 23.7; 38.6; 45.5; 46.7; 52.1; 58.5; 117.3; 122.5; 123.1; 123.4; 123.8; 129.4; 136.1; 149.2; 154.7; 176.0 (Figures S60, S61 and S103)

***N*-(2-(dimethylamino)ethyl)-3-((4-methylpiperazin-1-yl)methyl)-4-oxo-1,4-dihydro-1,10-phenanthroline-2-carboxamide (17d)**

Yield: 30.1 mg, 44%. Mp.: 161.3–163.0 °C. HRMS calcd for [M + H^+^] m/z = 423.2503, found m/z = 423.2502; ^1^H NMR (DMSO-d_6_): 2.17 (3H, s); 2.21 (6H, s); 2.28–2.35 (4H, m); 2.49 (2H, t, *J* = 6.40 Hz); 2.52 (4H, m); 3.54 (2H, q, *J* = 5.86 Hz); 3.73 (2H, s); 7.81–7.87 (2H, m); 8.15 (1H, d, *J* = 8.83 Hz); 8.56 (1H, d, *J* = 8.31 Hz); 9.08 (1H, d, *J* = 4.30 Hz); 11.11(1H, brs); 11.40 (1H, brs); ^13^C NMR (DMSO-d_6_): 38.4; 45.7; 46.1; 49.9; 51.4; 55.0; 58.7; 118.0; 122.5; 123.0; 123.3; 125.1; 129.5; 135.7; 137.3; 138.8; 141.1; 150.5; 162.7; 177.6 (Figures S62–S65 and S104)

***N*-(2-(dimethylamino)ethyl)-7-(morpholinomethyl)-6-oxo-6,9-dihydro-1*H*-pyrrolo[3,2-*h*]quinoline-8-carboxamide (18a)**

Yield: 25.1 mg, 39%. Mp.: decomposition over 210 °C. HRMS calcd for [M + H^+^] m/z = 398.2187, found m/z = 398.2183; ^1^H NMR (MeOH-d_4_): 2.38 (6H, s); 2.67 (2H, t, *J* = 6.53 Hz); 2.75–2.84 (4H, m); 3.66 (2H, t, *J* = 6.53 Hz); 3.73–3.83 (4H, m); 4.16 (2H, s); 6.67 (1H, d, *J* = 2.85 Hz); 7.48 (1H, d, *J* = 2.71 Hz); 7.65 (1H, d, *J* = 8.62 Hz); 7.86 (1H, d, *J* = 8.66 Hz); ^13^C NMR (MeOH-d_4_): 37.2; 44.3; 51.8; 52.3; 58.2; 66.1; 103.2; 106.7; 111.6; 114.7; 118.9; 119.2; 125.3; 129.6; 135.4; 144.0; 165.5; 190.0 (Figures S66, S67 and S105)

***N*-(2-(dimethylamino)ethyl)-6-oxo-7-(piperidin-1-ylmethyl)-6,9-dihydro-1*H*-pyrrolo[3,2-*h*]quinoline-8-carboxamide (18b)**

Yield: 21.1 mg, 33%. Mp.: decomposition over 215 °C. HRMS calcd for [M + H^+^] m/z = 396.2394, found m/z = 396.2390; ^1^H NMR (MeOH-d_4_): 1.64–1.94 (8H, m); 2.41 (6H, s); 2.71 (2H, t, *J* = 6.46 Hz); 3.19–3.30 (2H, m); 3.65 (2H, t, *J* = 6.37 Hz); 4.72 (2H, s); 6.60 (1H, d, *J* = 2.51 Hz); 7.38 (1H, d, *J* = 2.59 Hz); 7.58 (1H, d, *J* = 8.63 Hz); 7.88 (1H, d, *J* = 8.63 Hz); ^13^C NMR (MeOH-d_4_): 22.0; 23.4; 36.6; 44.2; 51.9; 53.3; 58.4; 102.6; 106.6; 110.6; 114.6; 118.6; 120.8; 123.4; 127.7; 136.6; 146.7; 165.2; 185.0 (Figures S68, S69 and S106)

***N*-(2-(dimethylamino)ethyl)-6-oxo-7-(pyrrolidin-1-ylmethyl)-6,9-dihydro-1*H*-pyrrolo[3,2-*h*]quinoline-8-carboxamide (18c)**

Yield: 17.9 mg, 29%. Mp.: decomposition over 220 °C. HRMS calcd for [M + H^+^] m/z = 382.2238, found m/z = 382.2235; ^1^H NMR (MeOH-d_4_): 2.11–2.18 (4H, m); 2.41 (6H, s); 2.71 (2H, t, *J* = 6.46 Hz); 3.40–3.47 (4H, m); 3.65 (2H, t, *J* = 7.73 Hz); 4.82 (2H, s); 6.59 (1H, d, *J* = 2.62 Hz); 7.37 (1H, d, *J* = 2.62 Hz); 7.57 (1H, d, *J* = 8.65 Hz); 7.89 (1H, d, *J* = 8.74 Hz); ^13^C NMR (MeOH-d_4_): 22.9; 36.6; 44.2; 51.5; 52.7; 58.3; 102.5; 108.4; 114.8; 118.4; 121.2; 123.3; 127.5; 131.5; 146.5; 170.0; 174.4 (Figures S70–S72 and S107)

***N*-(2-(dimethylamino)ethyl)-7-((4-methylpiperazin-1-yl)methyl)-6-oxo-6,9-dihydro-1*H*-pyrrolo [3,2-*h*]quinoline-8-carboxamide (18d)**

Yield: 15.3 mg, 23%. Mp.: decomposition over 210 °C. HRMS calcd for [M + H^+^] m/z = 411.2503, found m/z = 411.2502; ^1^H NMR (MeOH-d_4_): 2.34 (3H, s); 2.38 (6H, s); 2.51–2.69 (4H, m); 2.67 (2H, t, *J* = 6.53 Hz); 2.82–3.00 (4H, m); 3.65 (2H, t, *J* = 6.61 Hz); 4.26 (2H, s); 6.66 (1H, d, *J* = 2.82 Hz); 7.47 (1H, d, *J* = 3.01 Hz); 7.64 (1H, d, *J* = 8.73 Hz); 7.86 (1H, d, *J* = 8.59 Hz); ^13^C NMR (MeOH-d_4_): 37.1; 44.3; 44.5; 50.9; 52.0; 53.8; 58.2; 103.1; 110.1; 114.7; 119.1; 125.0; 126.2; 128.0; 166.1; 174.9 (Figures S73–S76 and S108)

## 4. Conclusions

The synthesis of KYNA derivatives condensed with an aromatic ring (tricyclic KYNA derivatives) has been investigated. The formation of the new ring systems was achieved using the Conrad–Limpach synthesis with different modifications for the corresponding tricyclic KYNA derivative. The optimization of the temperature was necessary for the enamine formation. In the case of the heterocyclic derivatives (**5**, **6**), the formation of undesired side products was observed at reflux temperature, and thus the reaction temperature was limited to r.t. In the case of 1,10-phenanthroline derivative **8**, ring closure had to be adjusted as the reflux temperature of DCB was not sufficient enough, but the desired product could be achieved using microwave reactor to reach 200 °C. The reactivity of these analogues has been investigated in the modified Mannich reaction using different cyclic secondary amines and paraformaldehyde as a representative aldehyde. The tricyclic KYNA derivatives showed lower reactivity compared to KYNA, as longer reaction times were required under the same conditions. In the case of the 1,10-phenanthroline derivative (**8**), a different solvent was employed. In terms of reactivity, noticeable differences were not observed between the different cyclic secondary amines. However, the work-up process was hindered by the presence of a stronger base in the case of piperidine, pyrrolidine, and *N*-methylpiperazine achieving lower yields. The tricyclic KYNA amide analogue of a KYNA amide with significant biological activity was also synthesized, and the reactivity of these compounds in the modified Mannich reaction were investigated as well. The reactivity of the amides bears similarity to the ester derivatives. However, in the case of derivative **15** condensed with pyrrole, the use of acidic additives was necessary, as the previous reaction conditions yielded a multicomponent reaction mixture. The synthesized ring systems bear resemblance to compounds with significant G-quadruplex binding capabilities. Consequently, they could be investigated as potential molecular scaffolds in the synthesis of anticancer and antiviral compounds.

## Figures and Tables

**Table 1:** Optimization of the ring closure reaction for compound **8** (using 1,2-dichlorobenzene as solvent and 2 h as reaction time).

Entry	Temperature (°C)	Conversion to 8 (%) ^1^	Conversion to 4-OH-1,10-phenantroline (%) ^1^
1	180	17	-
2	190	76	-
3	200	99	-
4	210	99	-
5	220	99	-
6	230	84	15

^1^ determined from the crude NMR spectra.

## Data Availability

Data are contained within the article and Supplementary Materials.

## Supplementary Materials

The following supporting information can be downloaded at: https://www.mdpi.com/article/10.3390/ijms26136248/s1.
