# Supplementary material for: Synthesis and Transformation of Tricyclic KYNA Derivatives
Source: Int J Mol Sci. 2025 Jun 28;26(13):6248. doi: 10.3390/ijms26136248 (PMC12250539; doi:10.3390/ijms26136248)
Supplement: Supplementary file 1 [file ijms-26-06248-s001.zip › ijms-3700713-supplementary.pdf]

Supplementary Information

Synthesis and Transformation of Tricyclic KYNA Derivatives

Julián Robin Sárik[1], István Szatmári[1,2,*] and Bálint Lőrinczi [1,*]
[1]Institute of Pharmaceutical Chemistry, University of Szeged, Eötvös u. 6, H-6720 Szeged, Hungary
[2]HUN-REN SZTE Stereochemistry Research Group, University of Szeged, Eötvös u. 6, H-6720 Szeged, Hungary

Contents

1. ^{1}H NMR, ^{13}C NMR, HSQC and HMBC spectra of synthesized compounds .... 2.
2. HR-MS spectra of synthesized compounds .... 43.

Diethyl 2-(naphthalen-1-ylamino)fumarate (**4**)

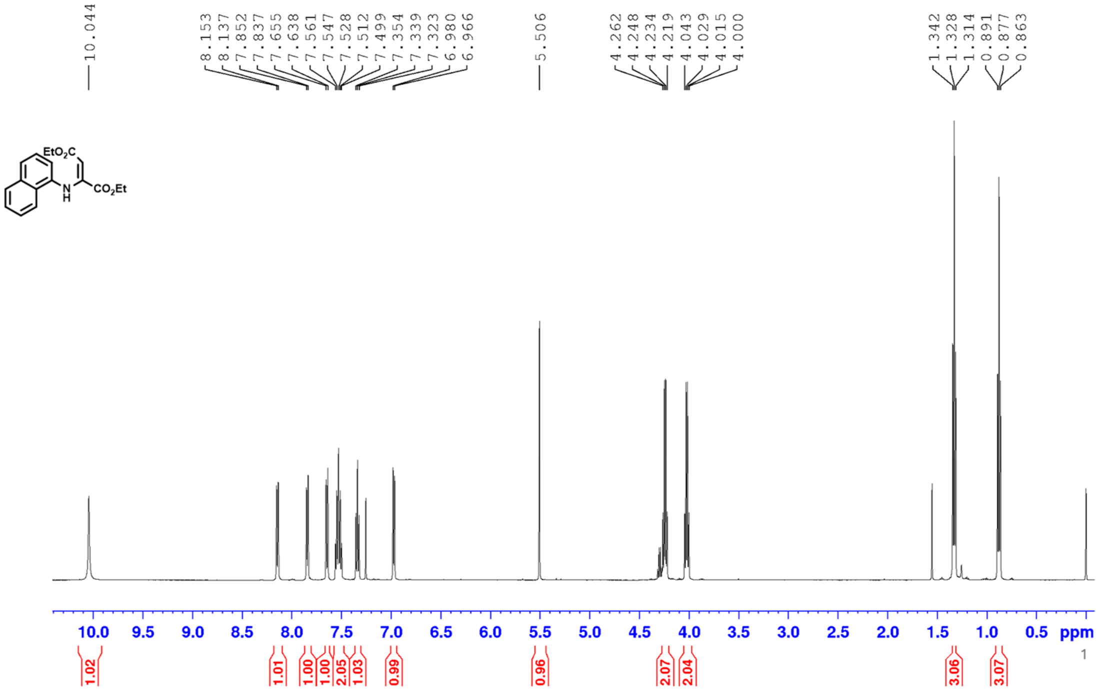

Figure S1. ^{1}H-NMR spectrum of **4**

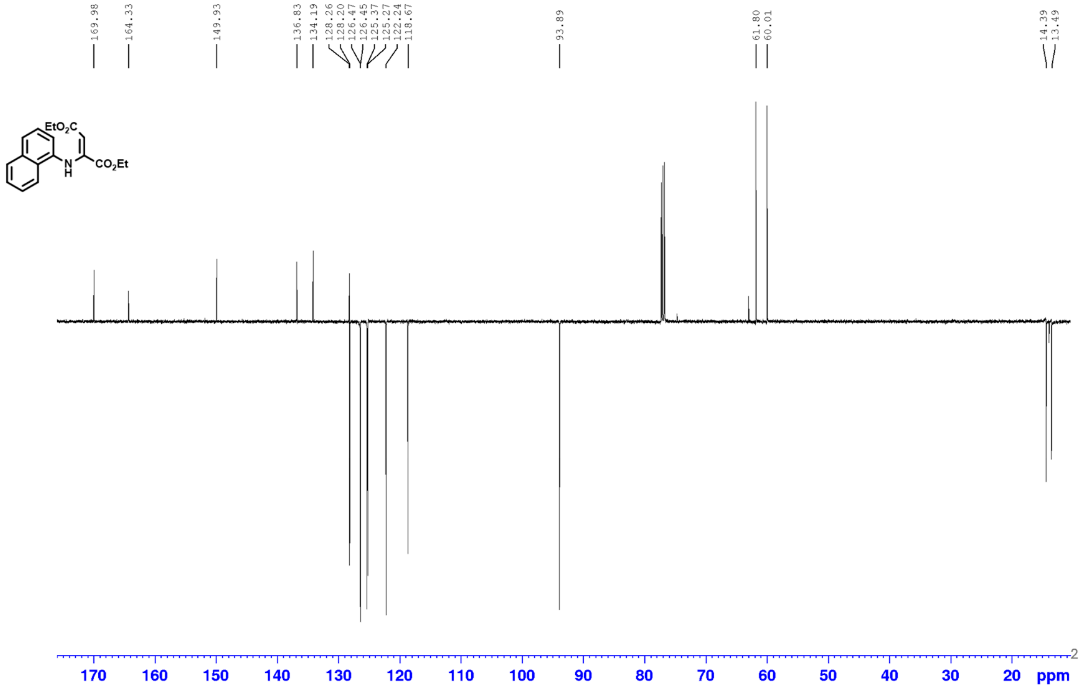

Figure S2. ^{13}C-NMR spectrum of **4**

Diethyl 2-(quinolin-8-ylamino)fumarate (**5**)

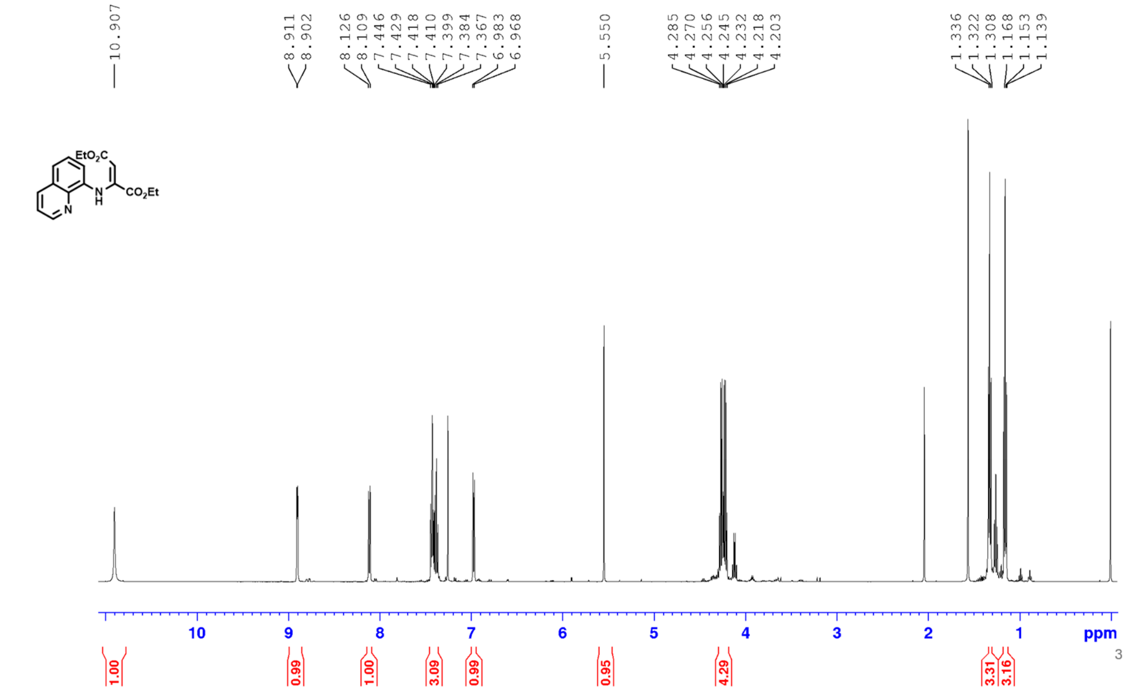

Figure S3. ^{1}H-NMR spectrum of **5**

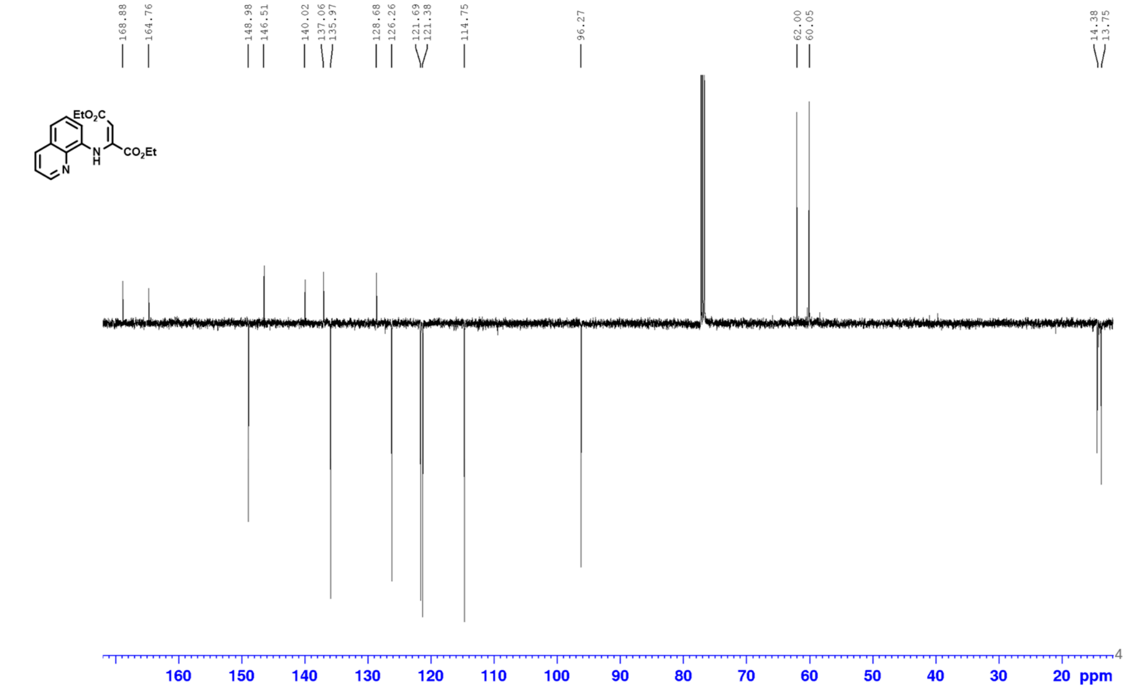

Figure S4. ^{13}C-NMR spectrum of **5**

Ethyl 4-oxo-1,4-dihydrobenzo[h]quinoline-2-carboxylate (**7**)

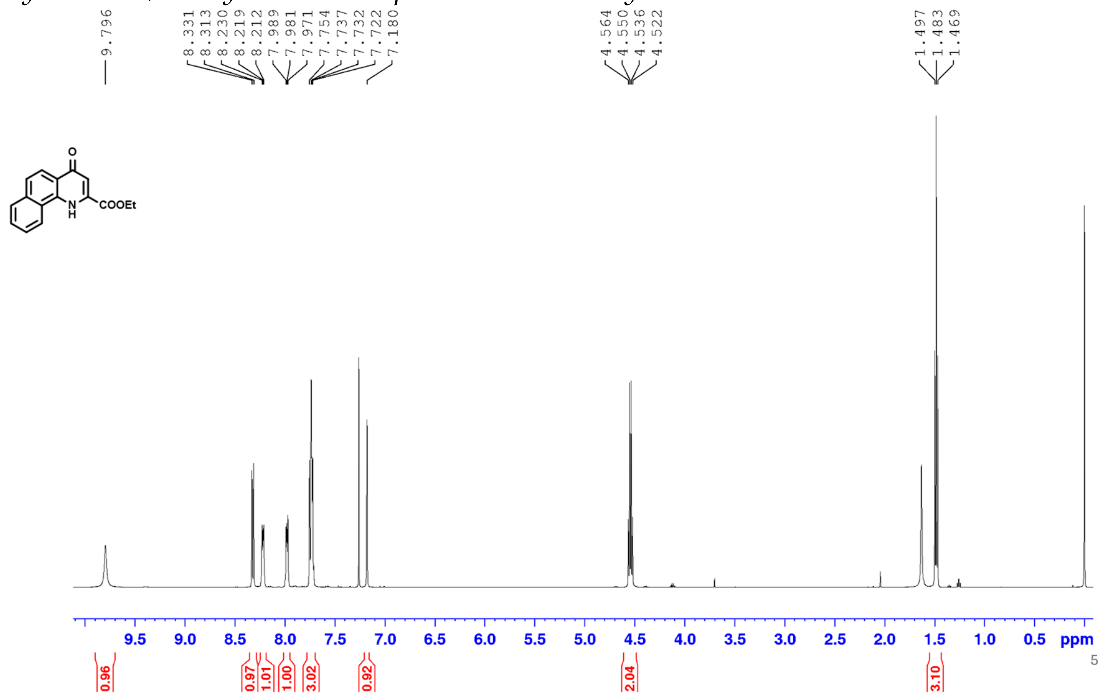

Figure S5. ^{1}H-NMR spectrum of **7**

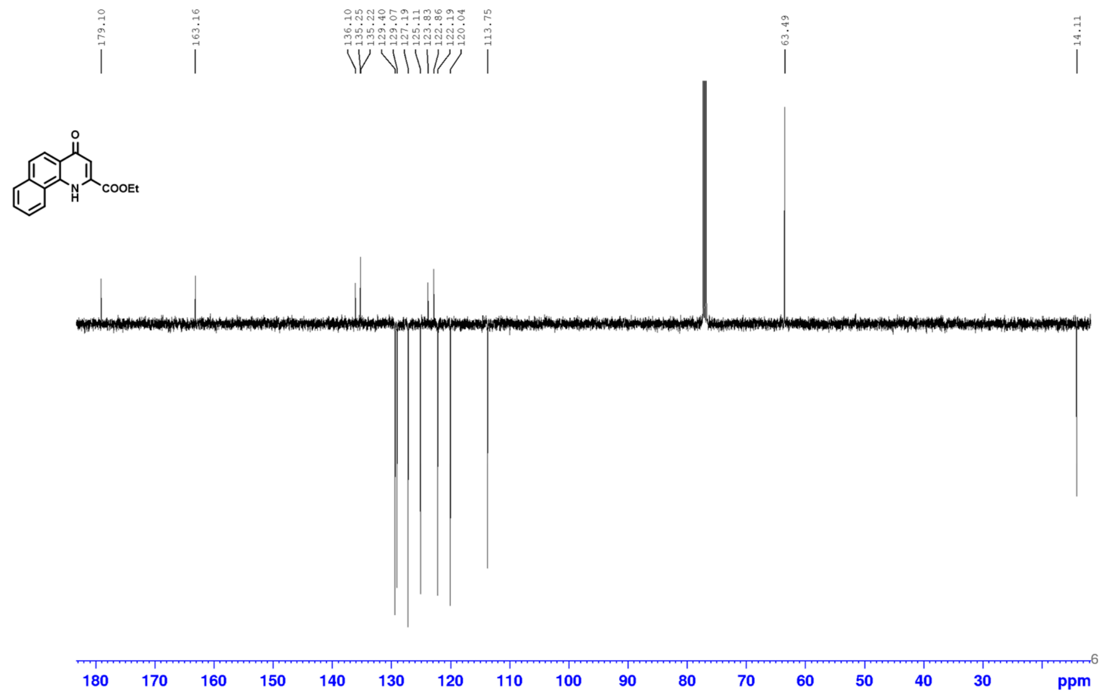

Figure S6. ^{13}C-NMR spectrum of **7**

Ethyl 4-oxo-1,4-dihydro-1,10-phenanthroline-2-carboxylate (**8**)

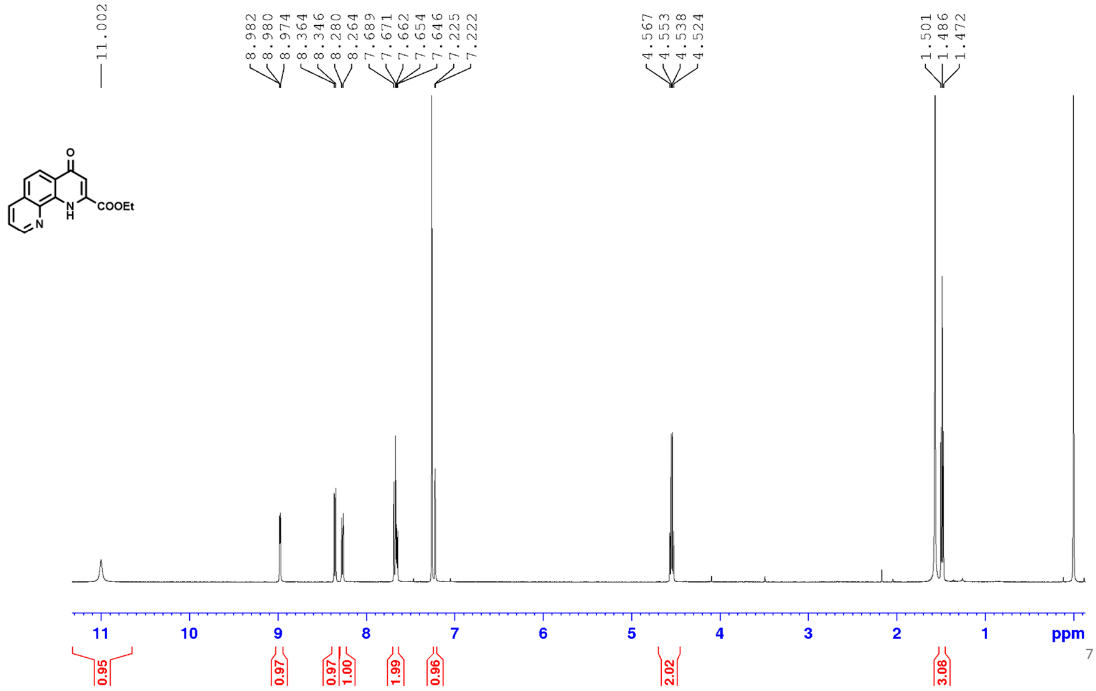

Figure S7. ^{1}H-NMR spectrum of **8**

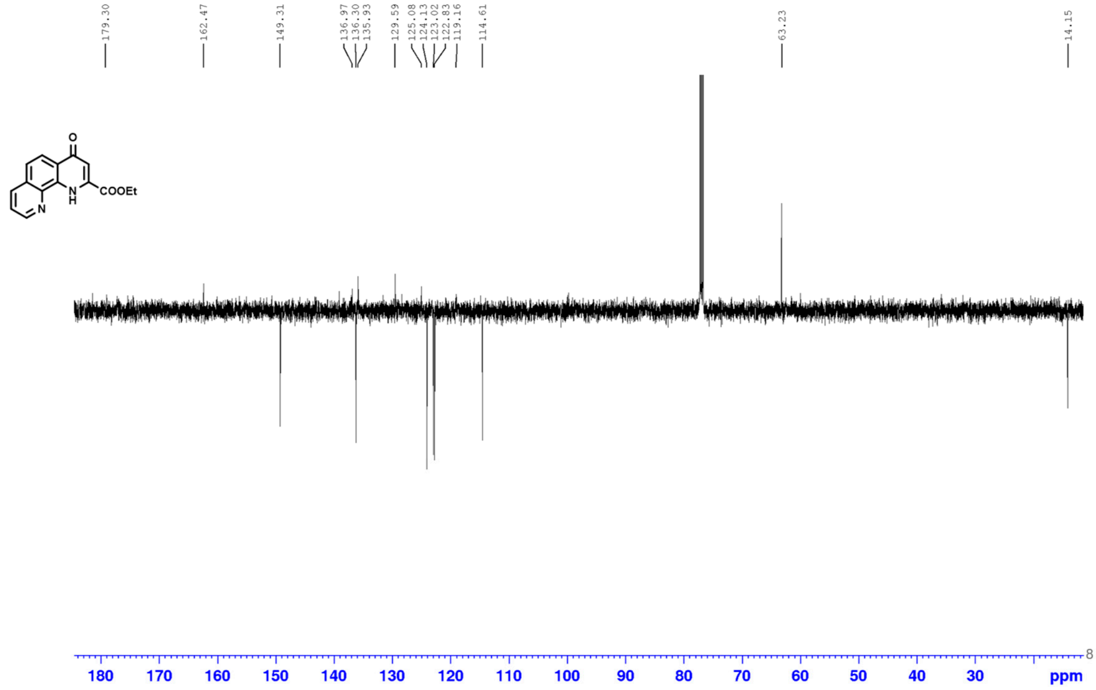

Figure S8. ^{13}C-NMR spectrum of **8**

Ethyl 6-oxo-6,9-dihydro-1H-pyrrolo[3,2-h]quinoline-8-carboxylate (**9**)

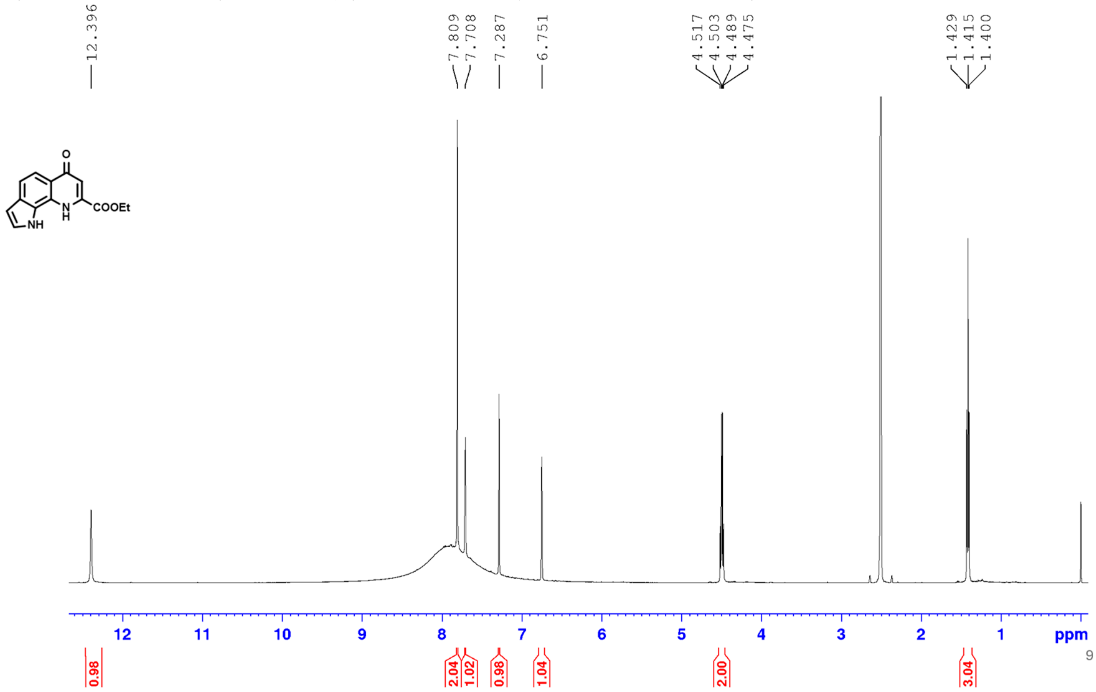

Figure S9. ^{1}H-NMR spectrum of **9** in DMSO-*d6* and 0,8 V/V% F_3C-COOH

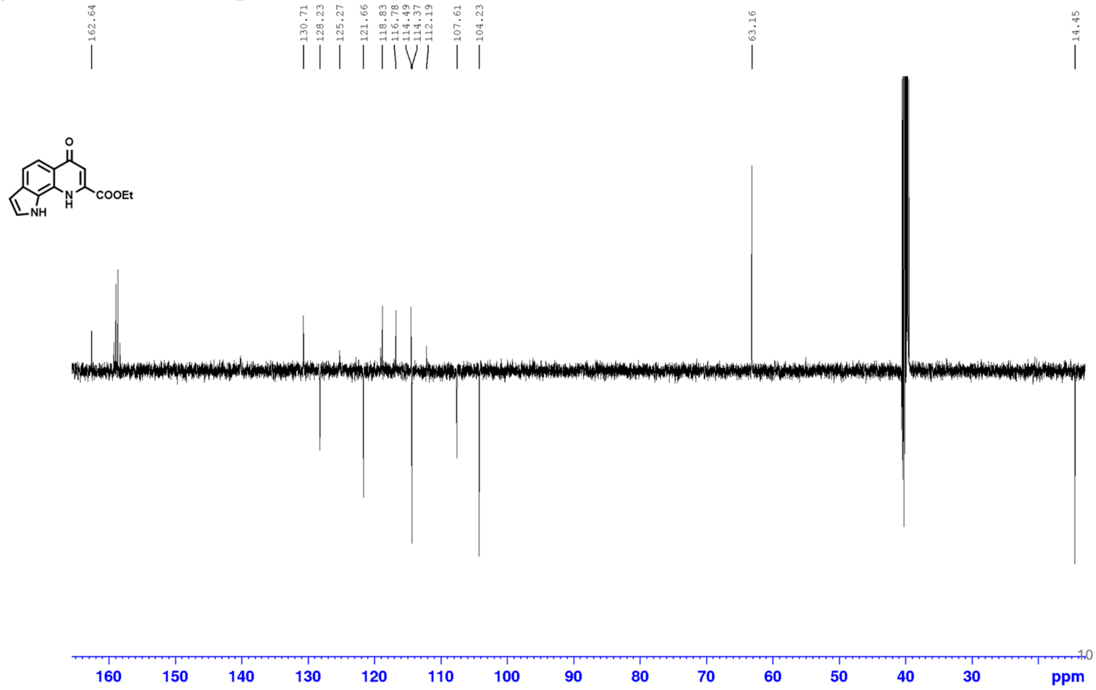

Figure S10. ^{13}C-NMR spectrum of **9** in DMSO-*d6* and 0,8 V/V% F_3C-COOH

3-(Morpholinomethyl)-4-oxo-1,4-dihydrobenzo[h]quinoline-2-carboxylic acid (**10a**)

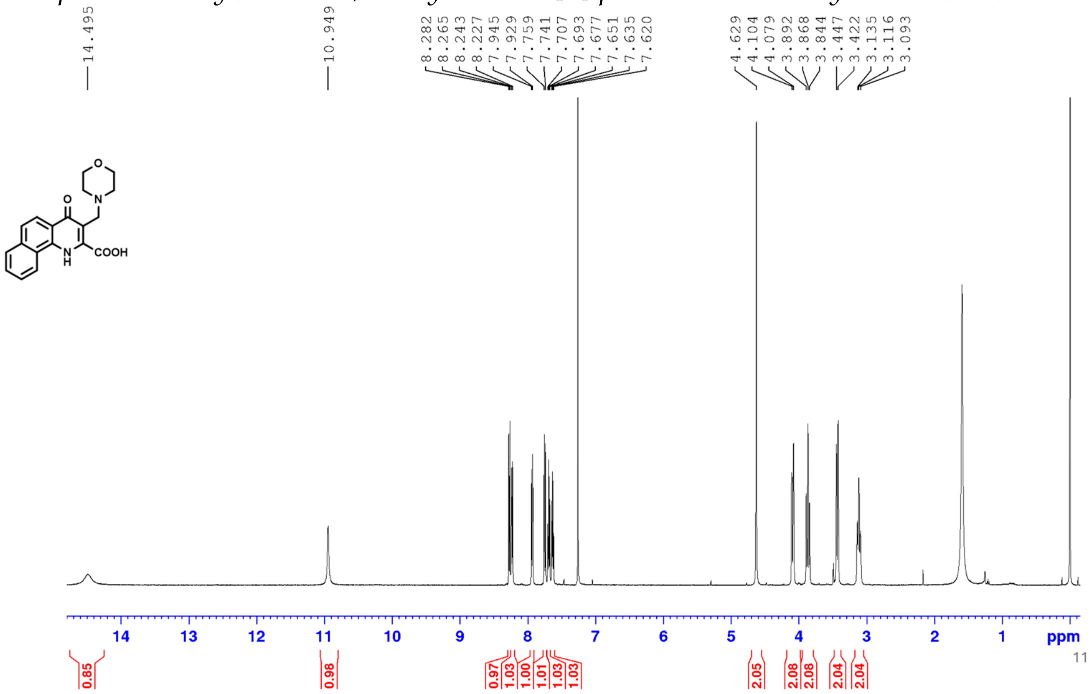

Figure S11. ^{1}H-NMR spectrum of **10a**

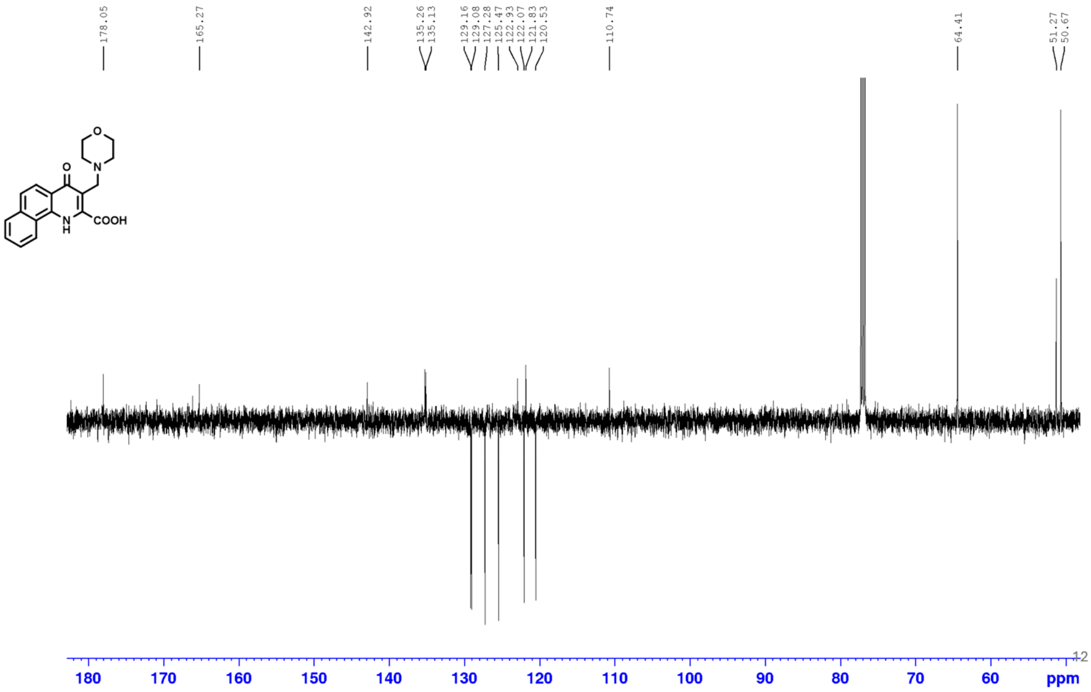

Figure S12. ^{13}C-NMR spectrum of **10a**

4-Oxo-3-(piperidin-1-ylmethyl)-1,4-dihydrobenzo[h]quinoline-2-carboxylic acid (**10b**)

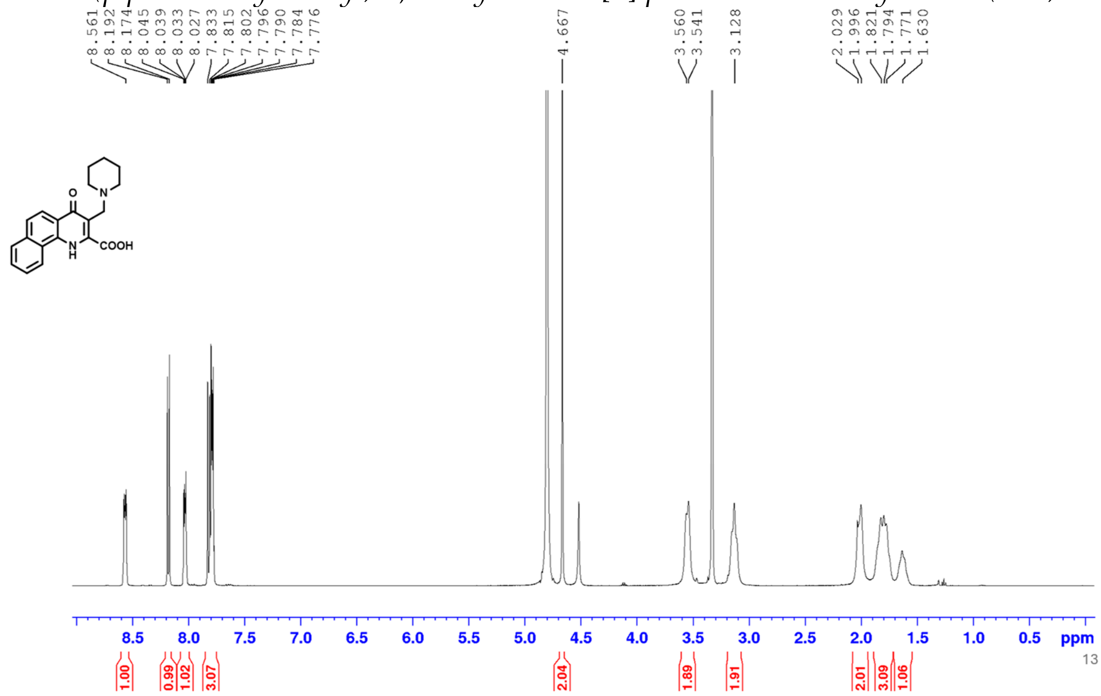

Figure S13. ^{1}H-NMR spectrum of **10b**

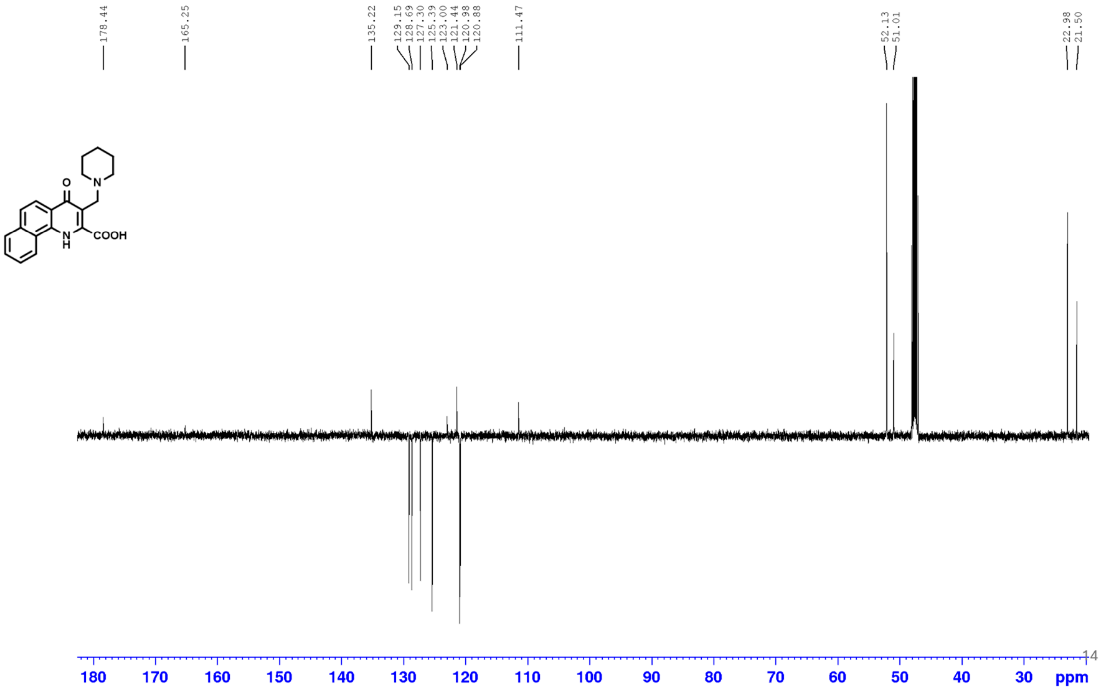

Figure S14. ^{13}C-NMR spectrum of **10b**

4-Oxo-3-(pyrrolidin-1-ylmethyl)-1,4-dihydrobenzo[h]quinoline-2-carboxylic acid (**10c**)

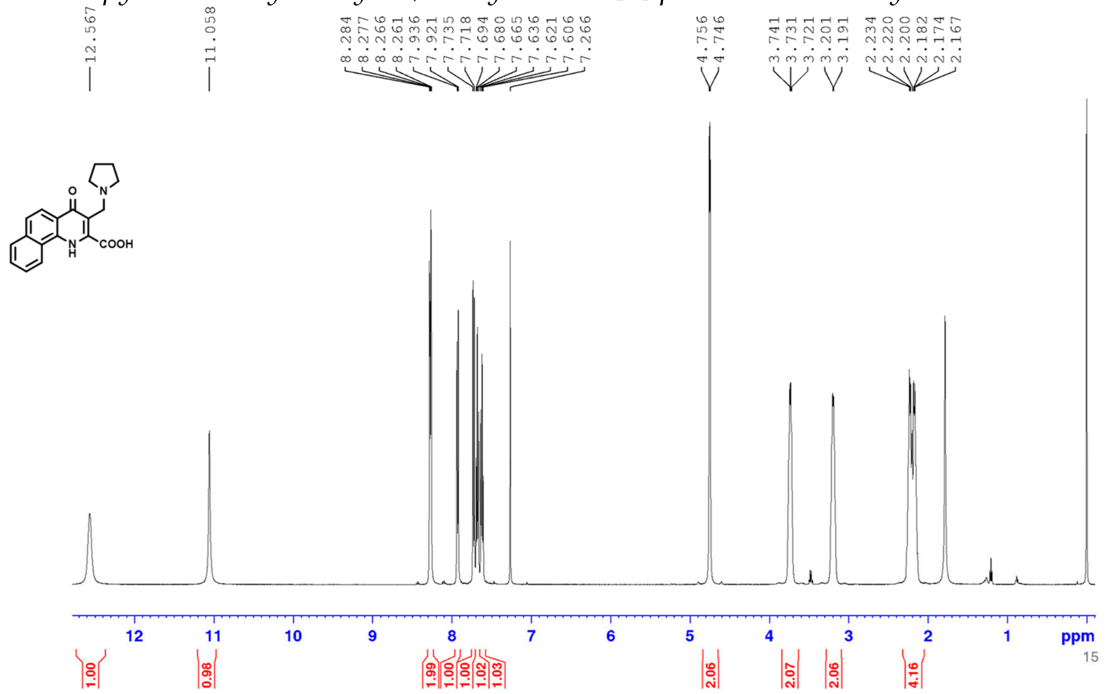

Figure S15. ^{1}H-NMR spectrum of **10c**

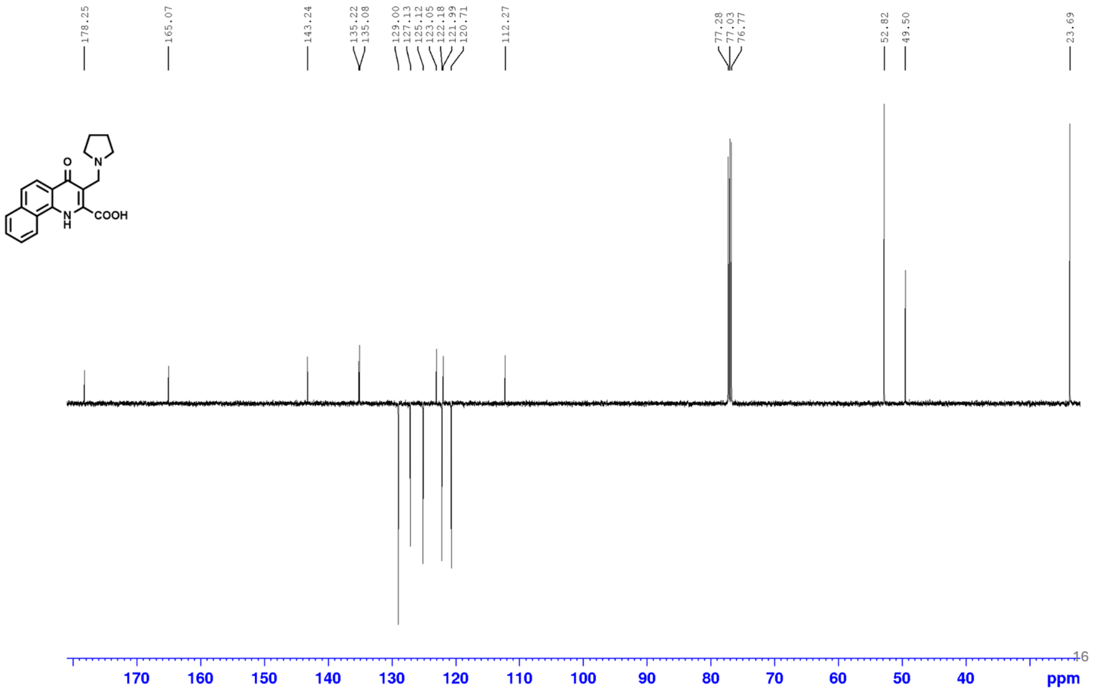

Figure S16. ^{13}C-NMR spectrumof **10c**

3-((4-Methylpiperazin-1-yl)methyl)-4-oxo-1,4-dihydrobenzo[h]quinoline-2-carboxylic acid (**10d**)

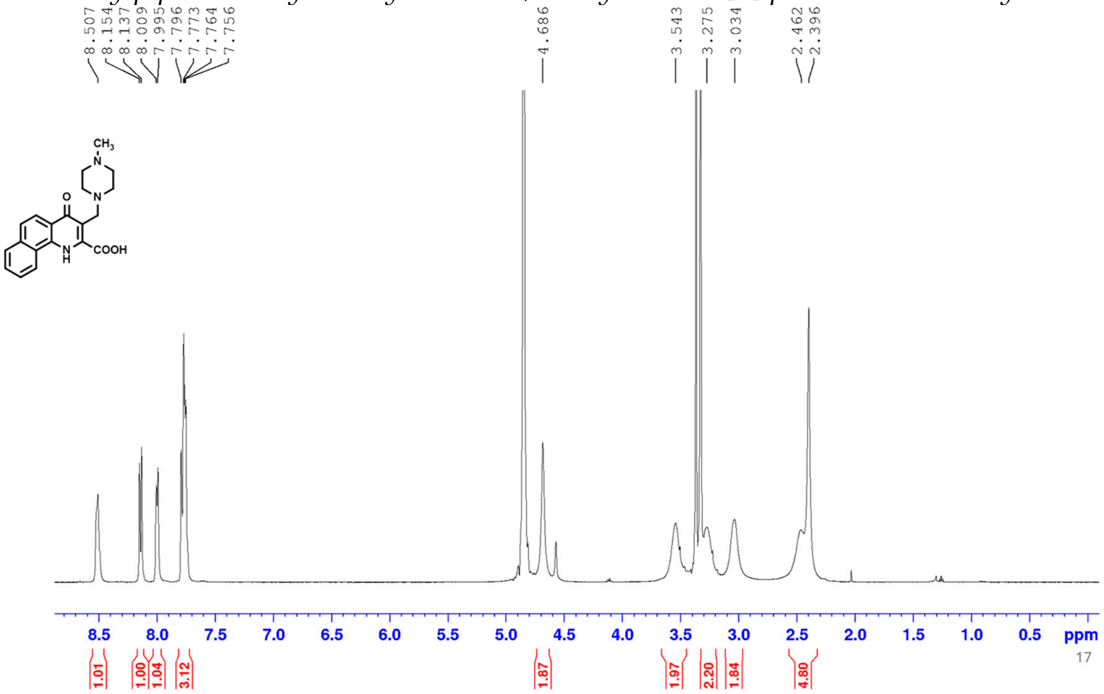

Figure S17. ^{1}H-NMR spectrum of **10d**

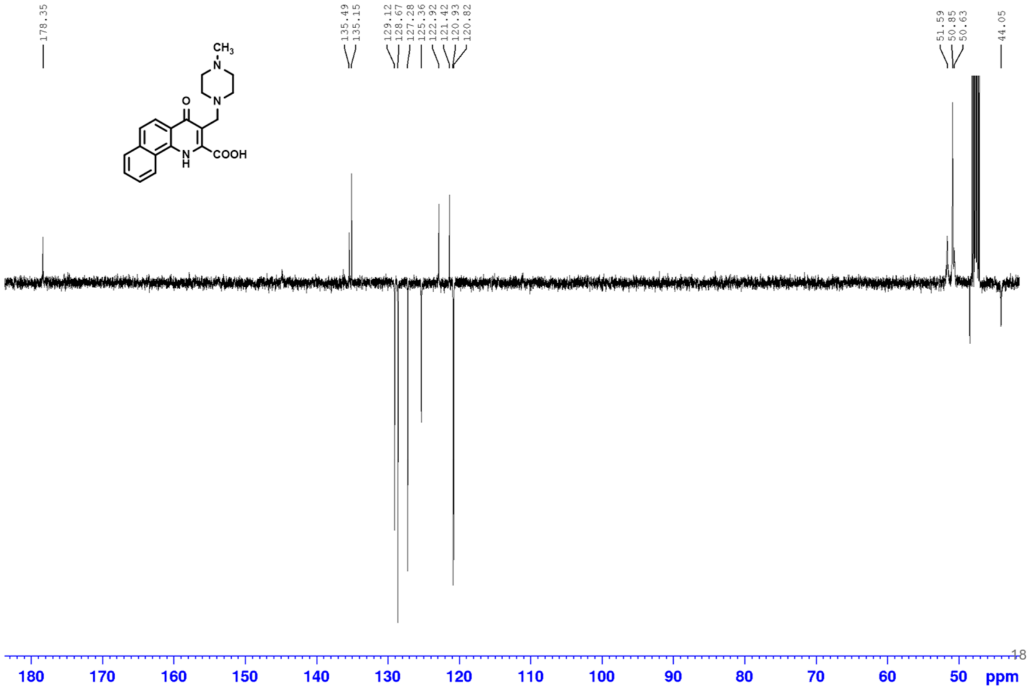

Figure S18. ^{13}C-NMR spectrumof **10d**

*3-(Morpholinomethyl)-4-oxo-1,4-dihydro-1,10-phenanthroline-2-carboxylic acid (**11a**)*

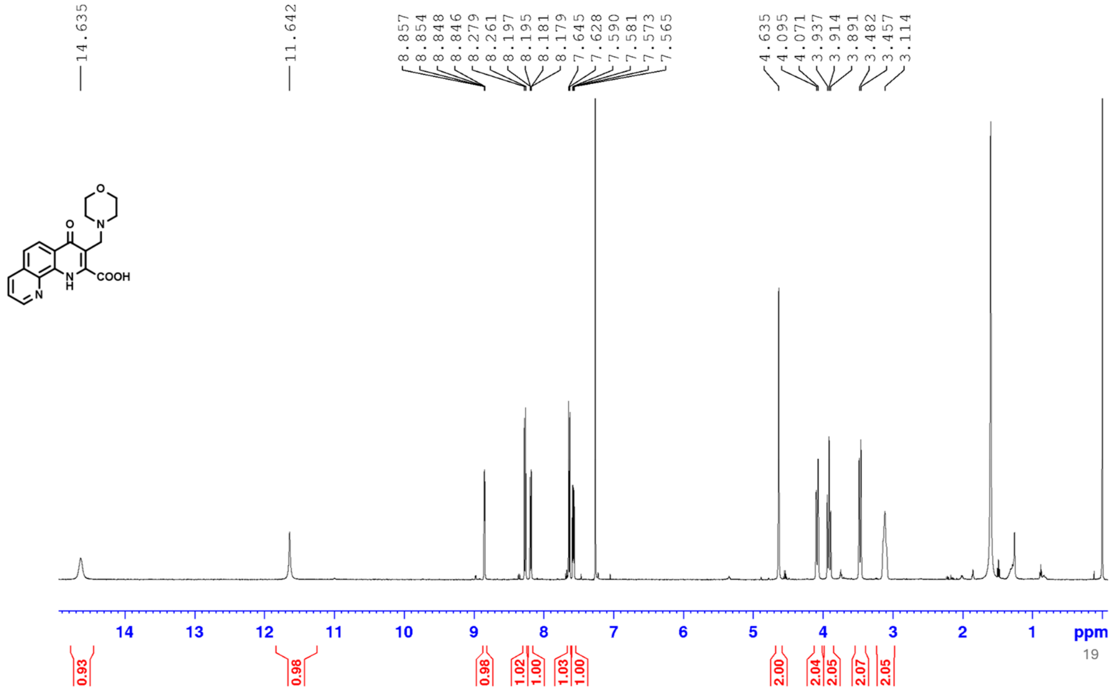

Figure S19. ^{1}H-NMR spectrum of **11a**

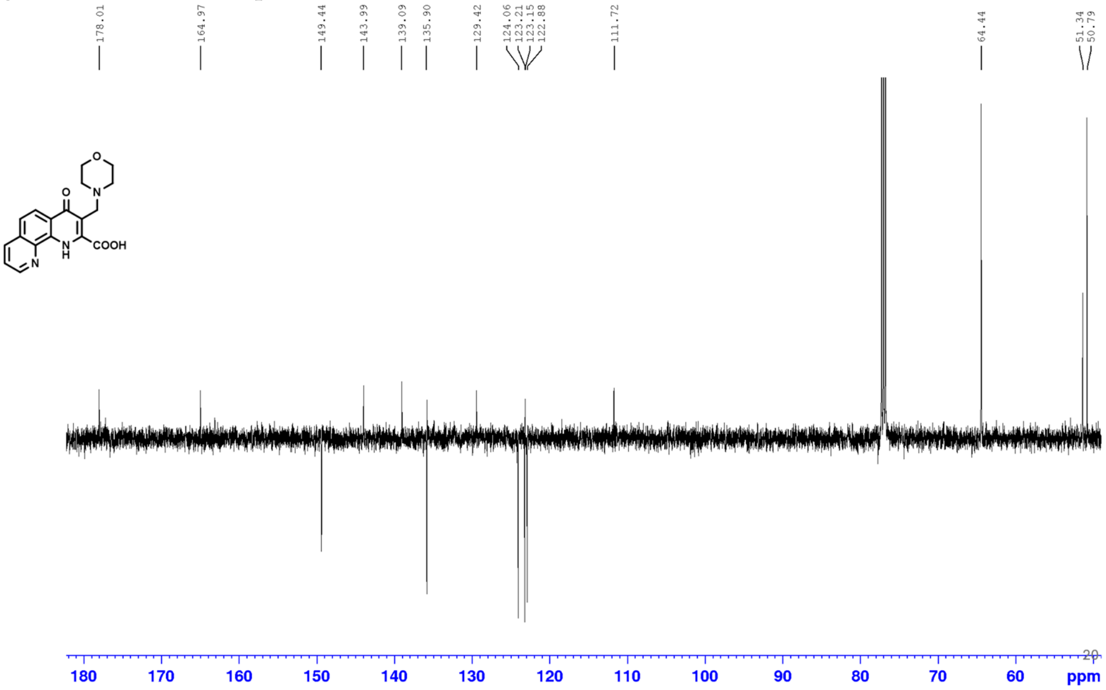

Figure S20. ^{13}C-NMR spectrum of **11a**

4-Oxo-3-(piperidin-1-ylmethyl)-1,4-dihydro-1,10-phenanthroline-2-carboxylic acid (**11b**)

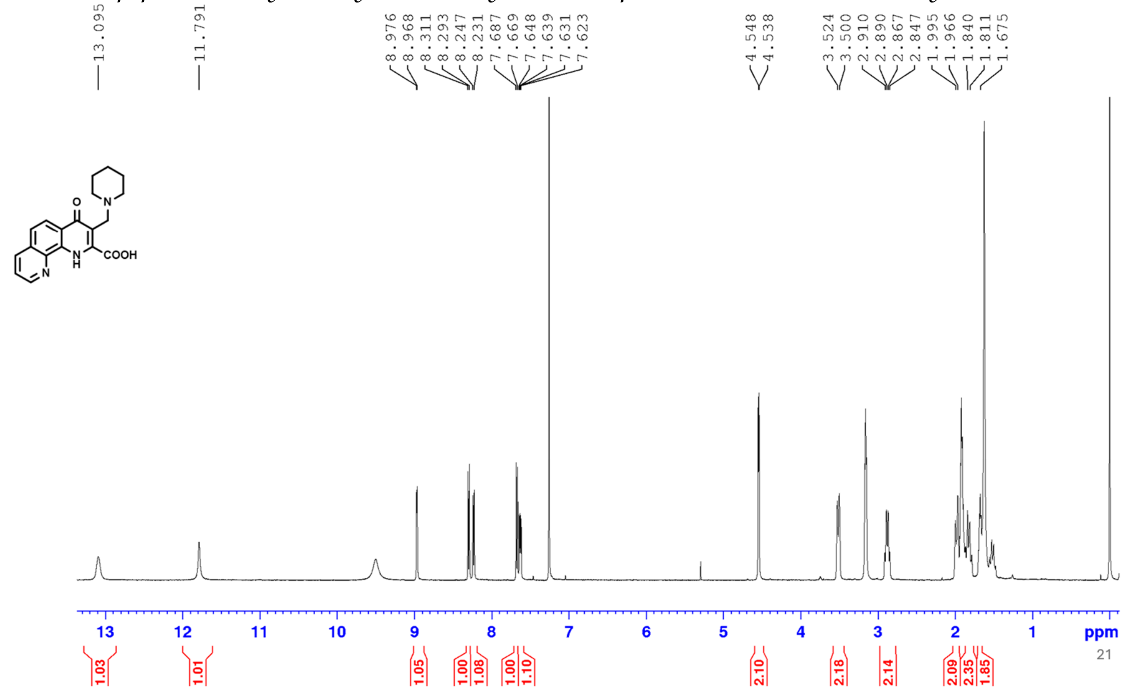

Figure S21. ^{1}H-NMR spectrum of **11b**

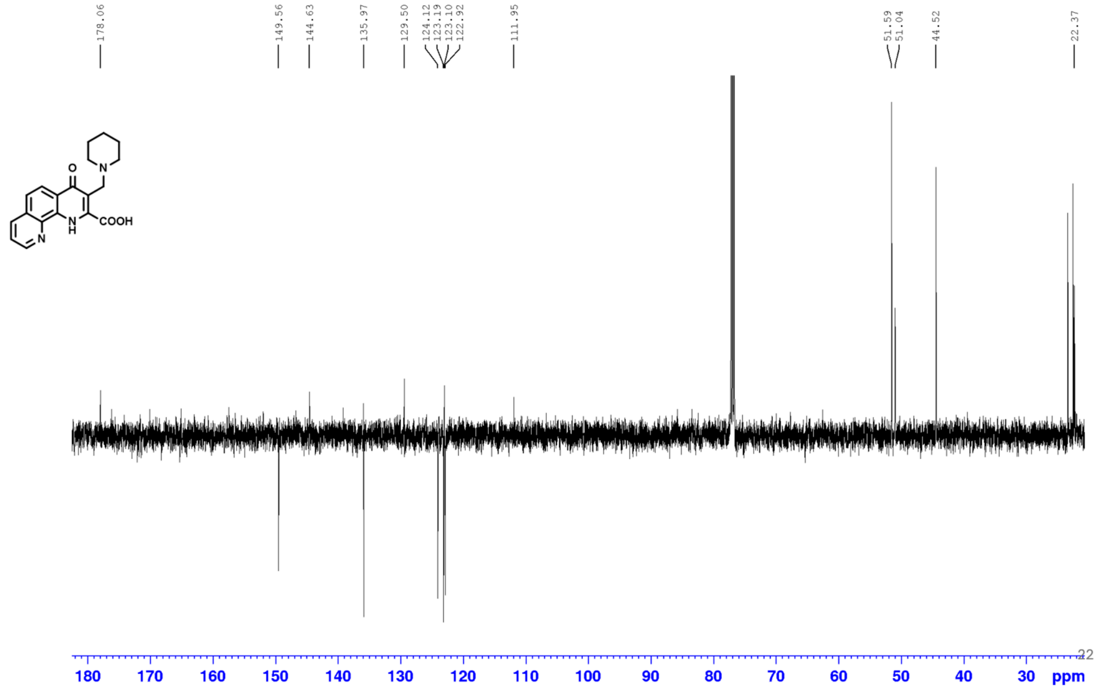

Figure S22. ^{13}C-NMR spectrum of **11b**

4-Oxo-3-(pyrrolidin-1-ylmethyl)-1,4-dihydro-1,10-phenanthroline-2-carboxylic acid (**11c**)

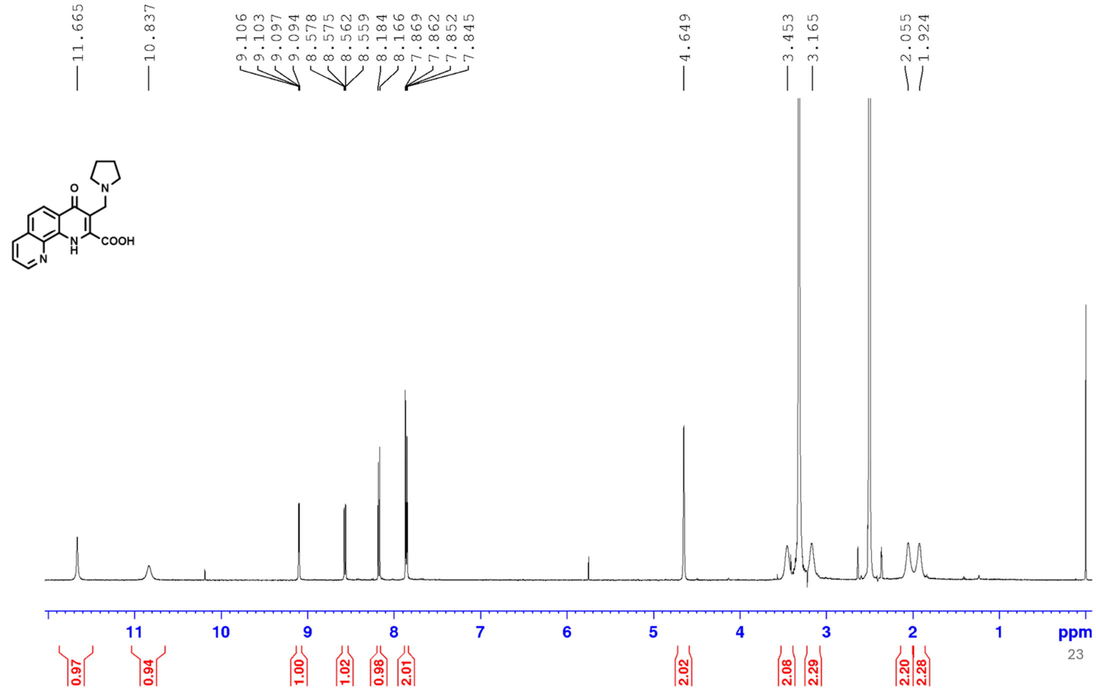

Figure S23. ^{1}H-NMR spectrum of **11c**

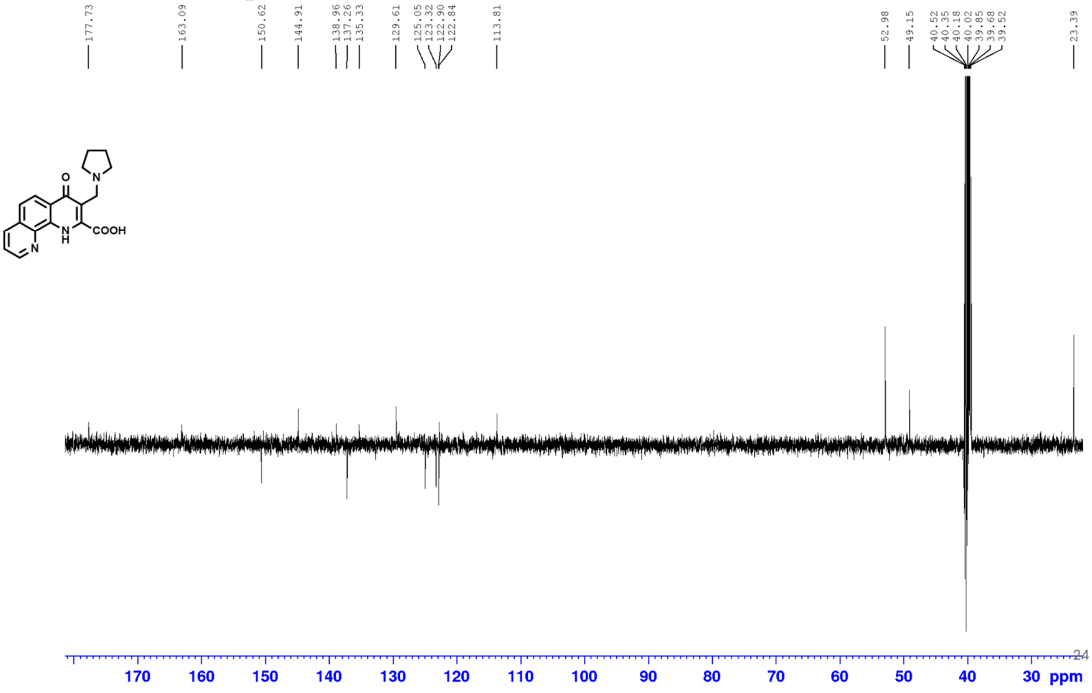

Figure S24. ^{13}C-NMR spectrum of **11c**

*3-((4-Methylpiperazin-1-yl)methyl)-4-oxo-1,4-dihydro-1,10-phenanthroline-2-carboxylic acid (**11d**)*

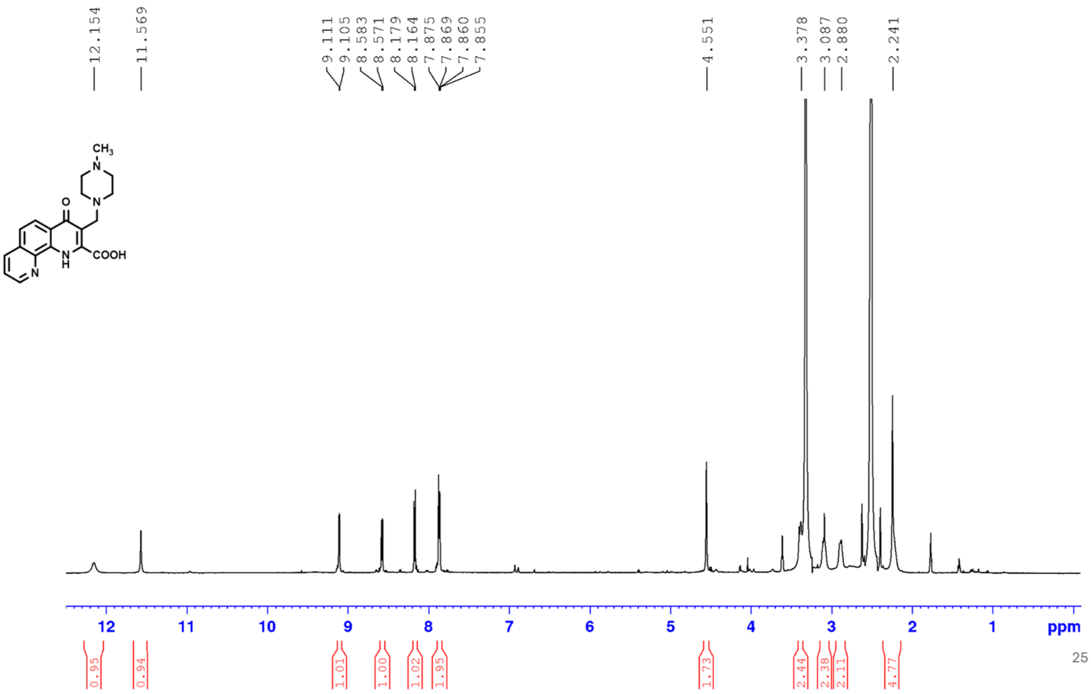

Figure S25. ^{1}H-NMR spectrum of **11d**

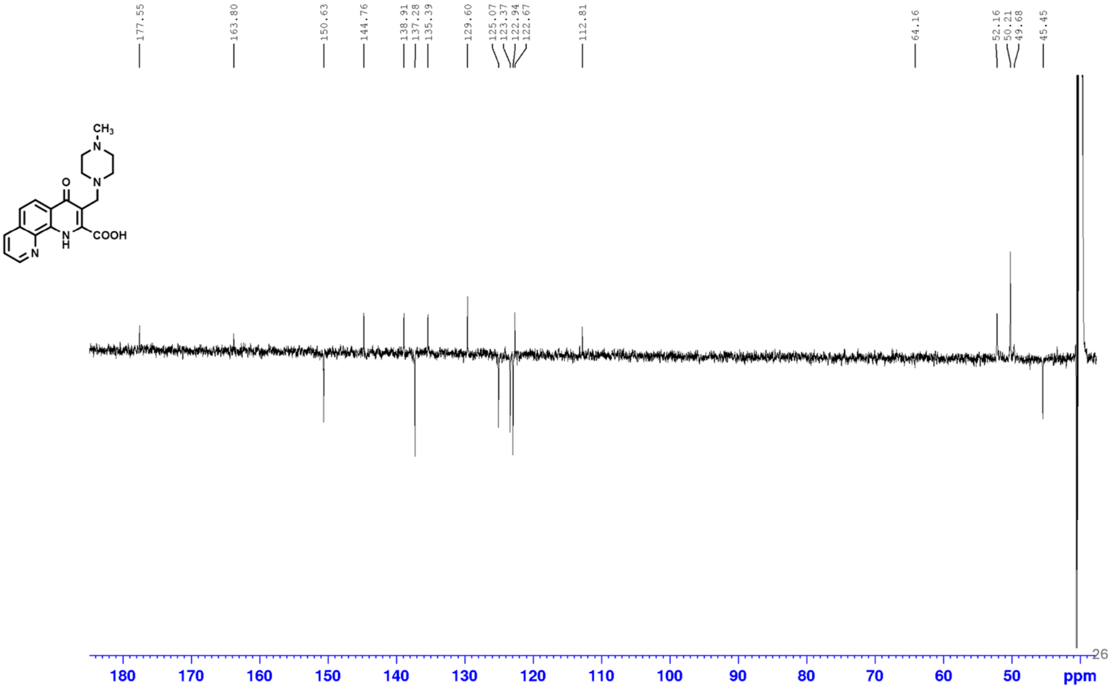

Figure S26. ^{13}C-NMR spectrum of **11d**

7-(Morpholinomethyl)-6-oxo-6,9-dihydro-1H-pyrrolo[3,2-h]quinoline-8-carboxylic acid (**12a**)

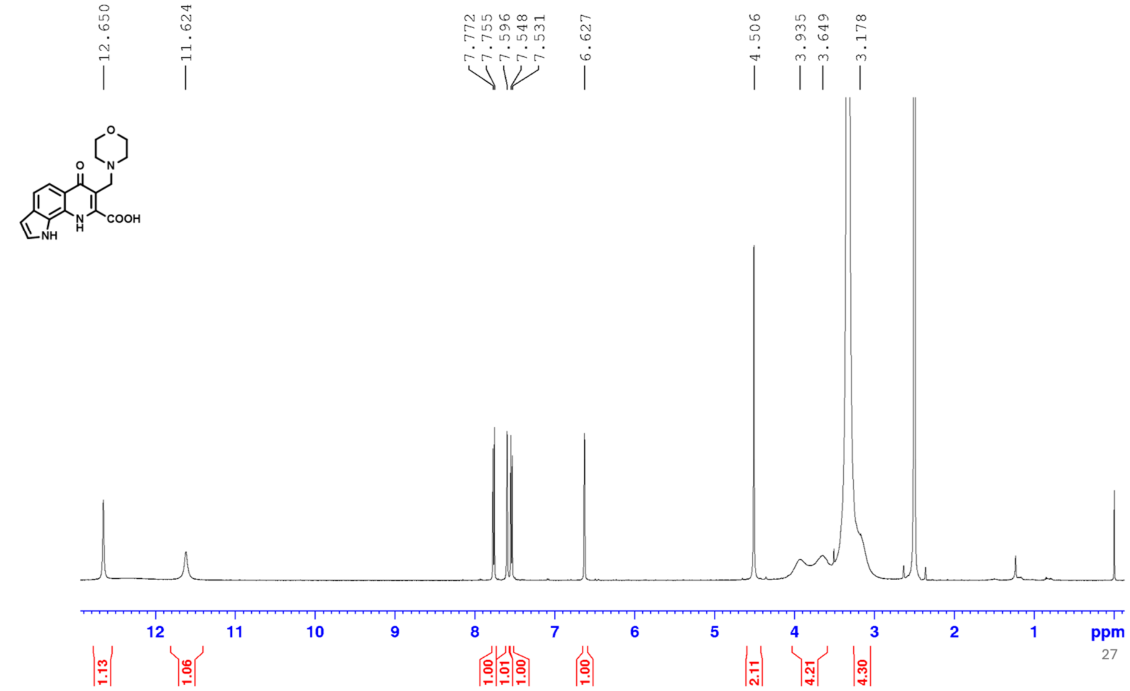

Figure S27. ¹H-NMR spectrum of **12a**

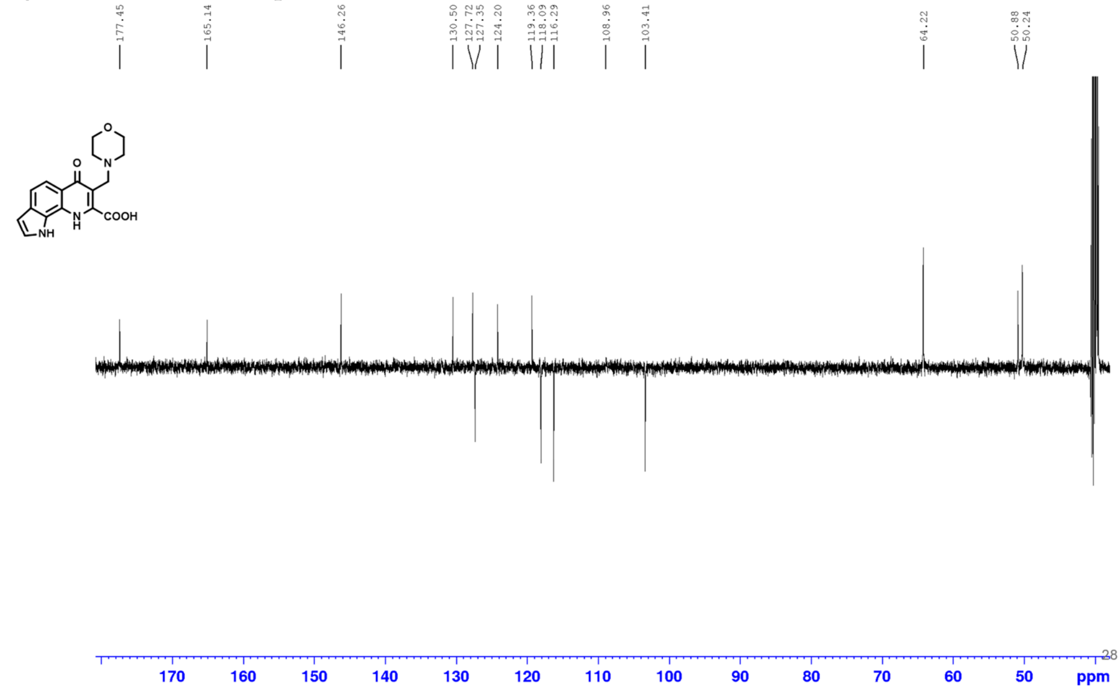

Figure S28. ¹³C-NMR spectrum of **12a**

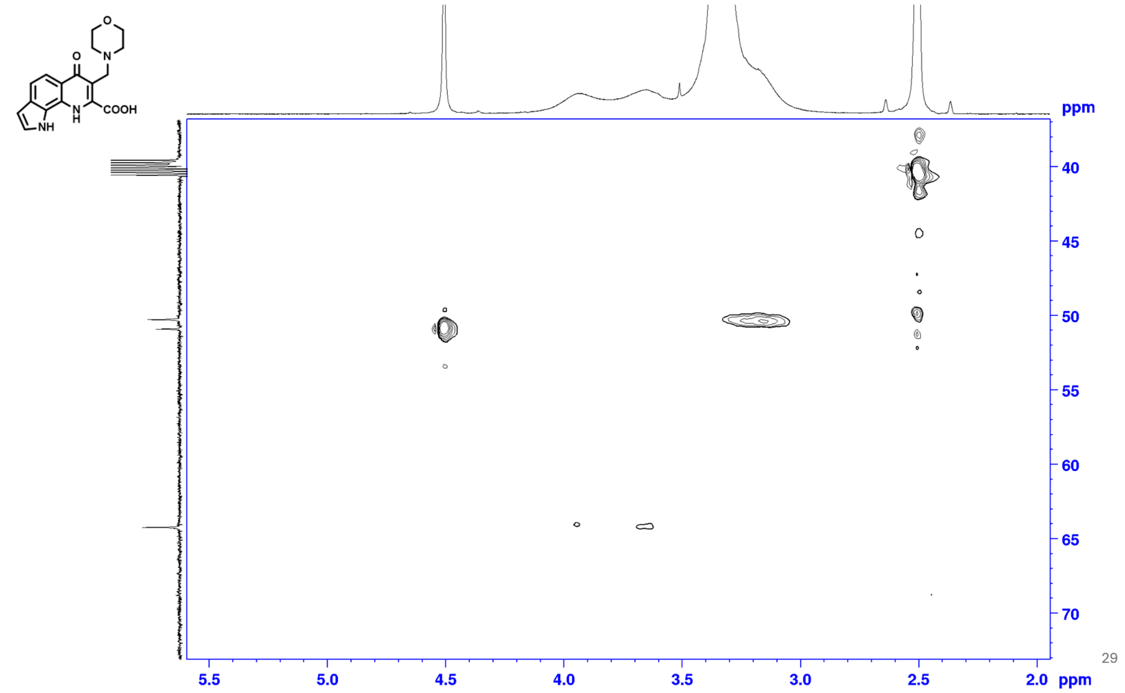

Figure S29. HSQC NMR spectrum of **12a**

6-oxo-7-(piperidin-1-ylmethyl)-6,9-dihydro-1H-pyrrolo[3,2-h]quinoline-8-carboxylic acid (**12b**)

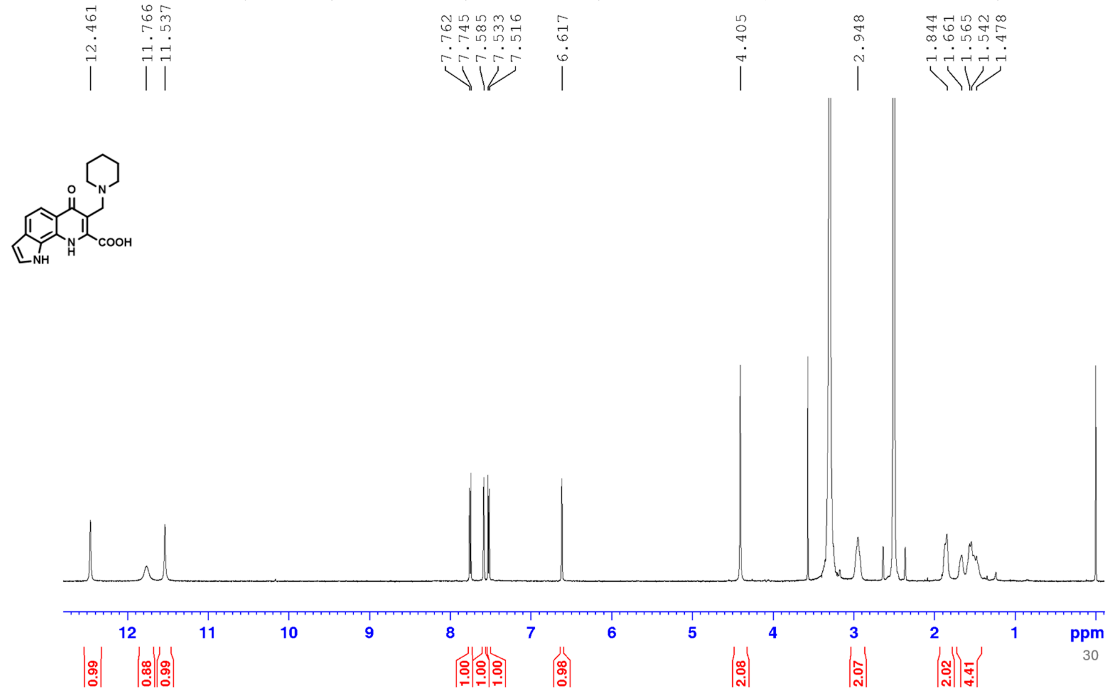

Figure S30. ^{1}H-NMR spectrum of **12b**

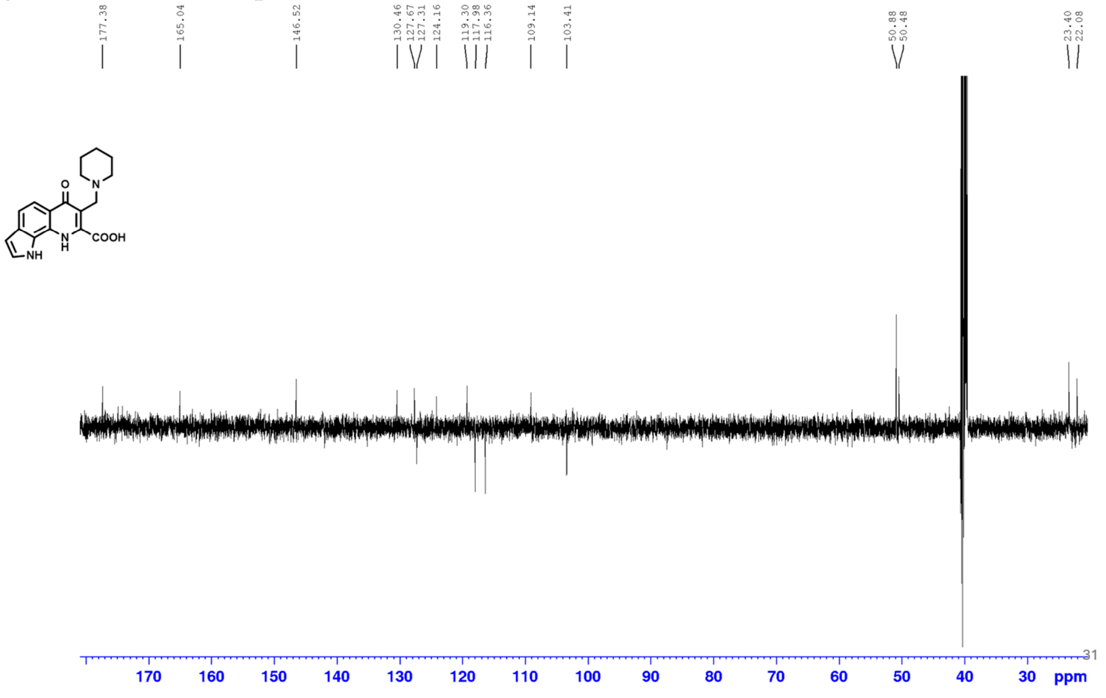

Figure S31. ^{13}C-NMR spectrum of **12b**

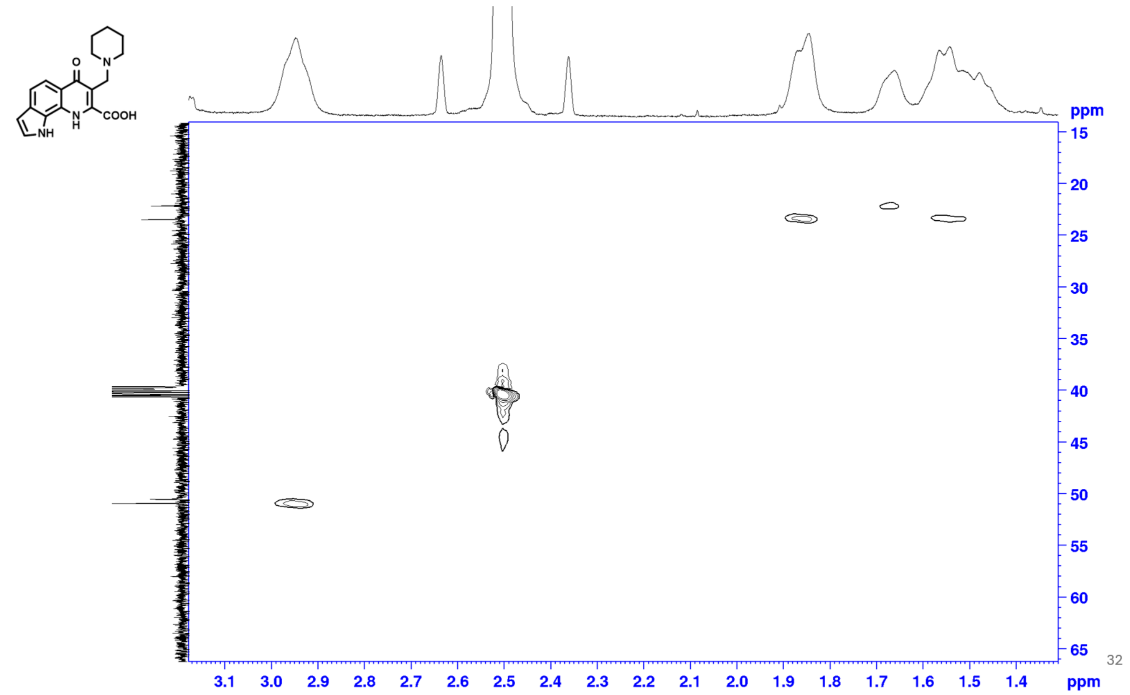

Figure S32. HSQC NMR spectrum of **12b**

6-oxo-7-(pyrrolidin-1-ylmethyl)-6,9-dihydro-1H-pyrrolo[3,2-h]quinoline-8-carboxylic acid (**12c**)

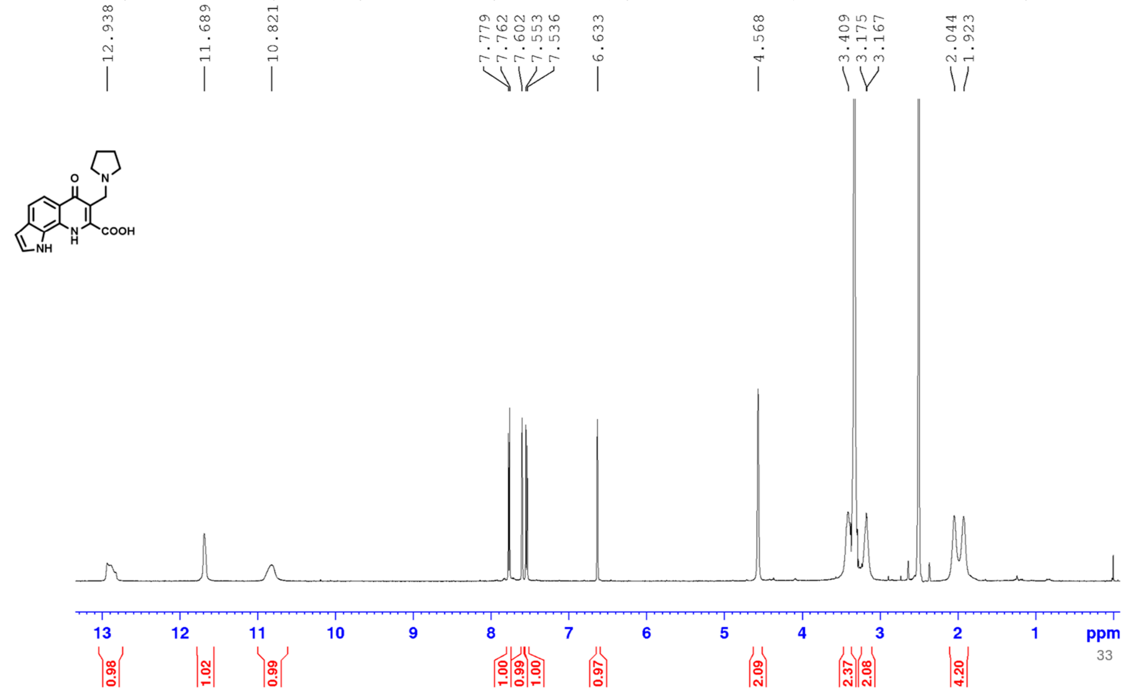

Figure S33. ^{1}H-NMR spectrum of **12c**

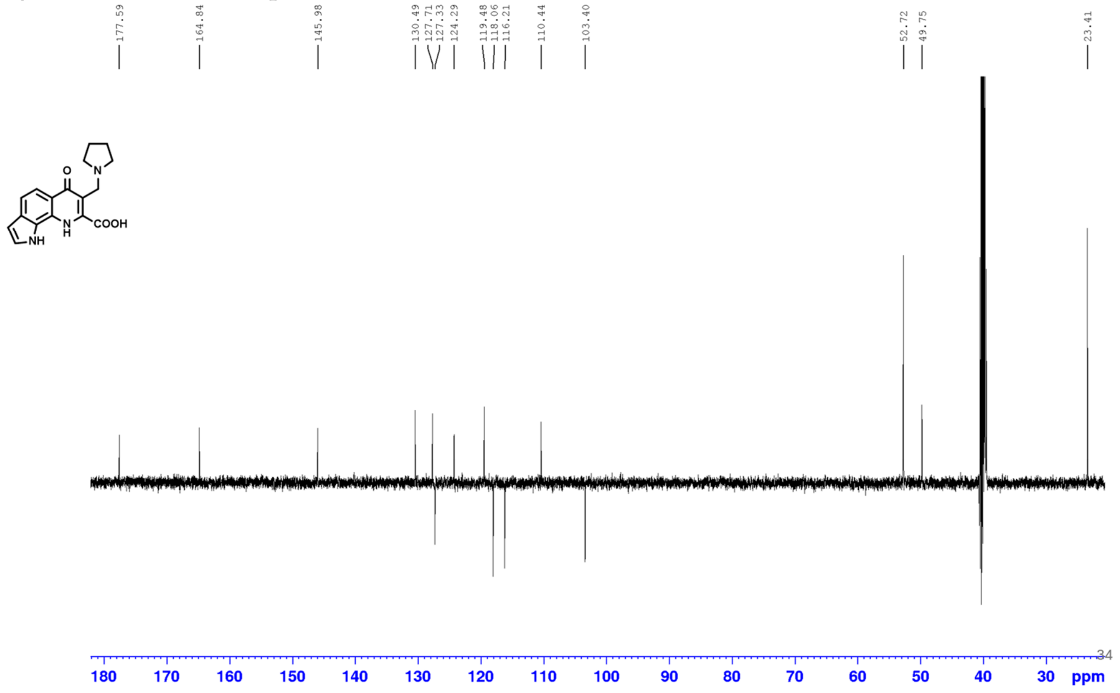

Figure S34. ^{13}C-NMR spectrum of **12c**

*7-((4-methylpiperazin-1-yl)methyl)-6-oxo-6,9-dihydro-1H-pyrrolo[3,2-h]quinoline-8-carboxylic acid (**12d**)*

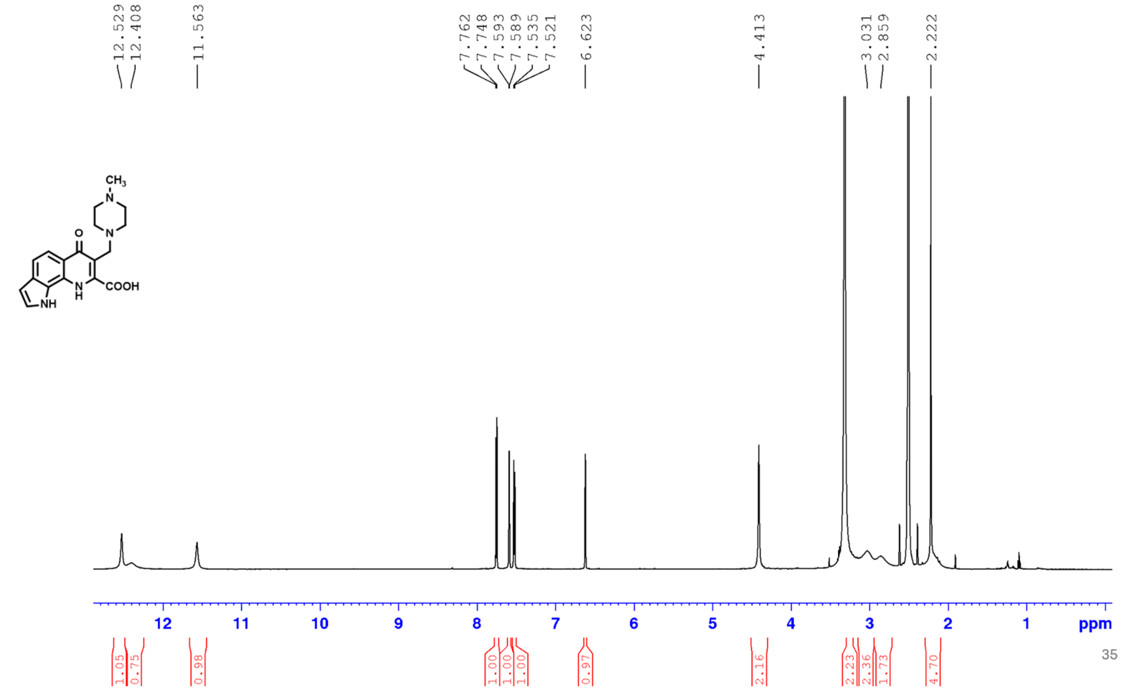

Figure S35. ¹H-NMR spectrum of **12d**

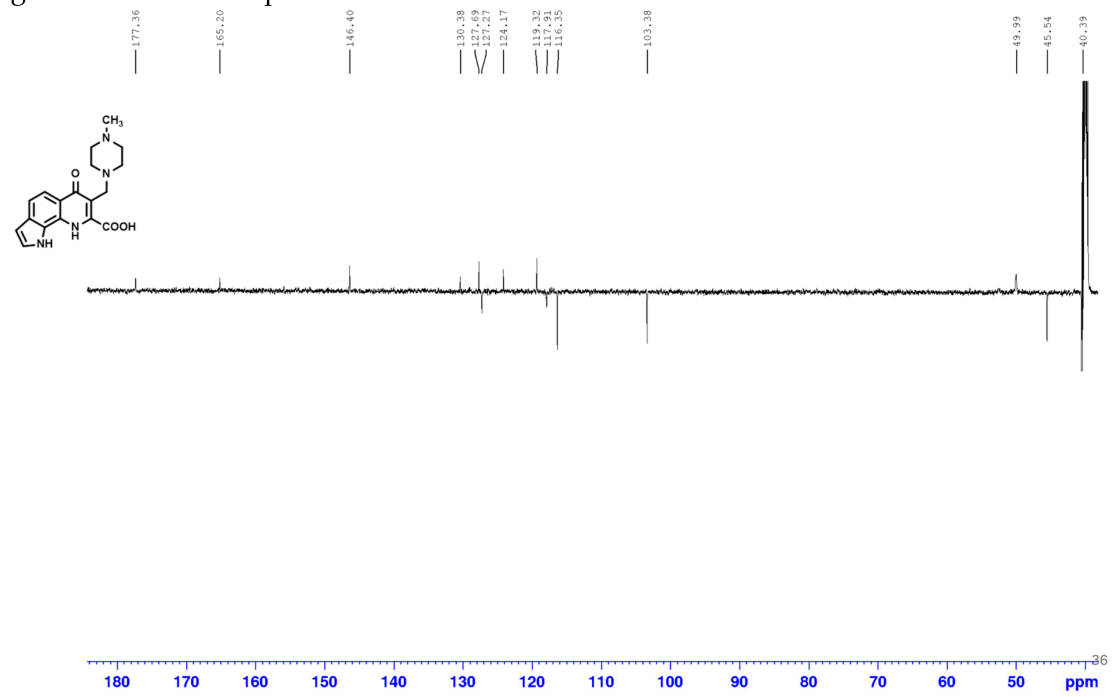

Figure S36. ¹³C-NMR spectrum of **12d**

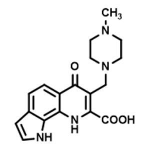

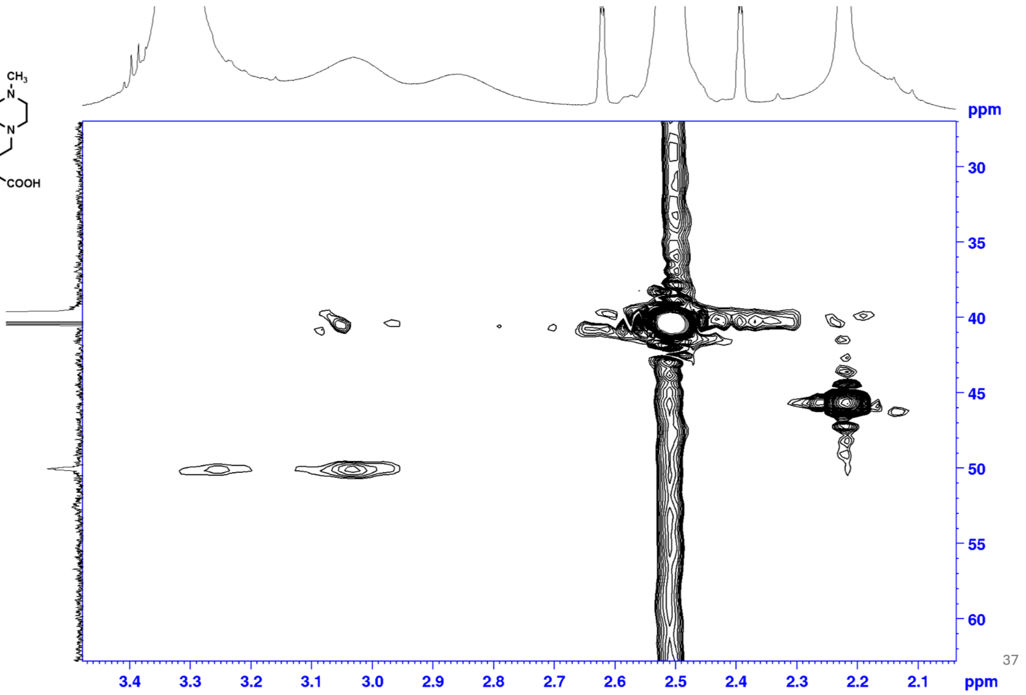

Figure S37. HSQC NMR spectrum of **12d**

N-(2-(dimethylamino)ethyl)-4-oxo-1,4-dihydrobenzo[h]quinoline-2-carboxamide (**13**)

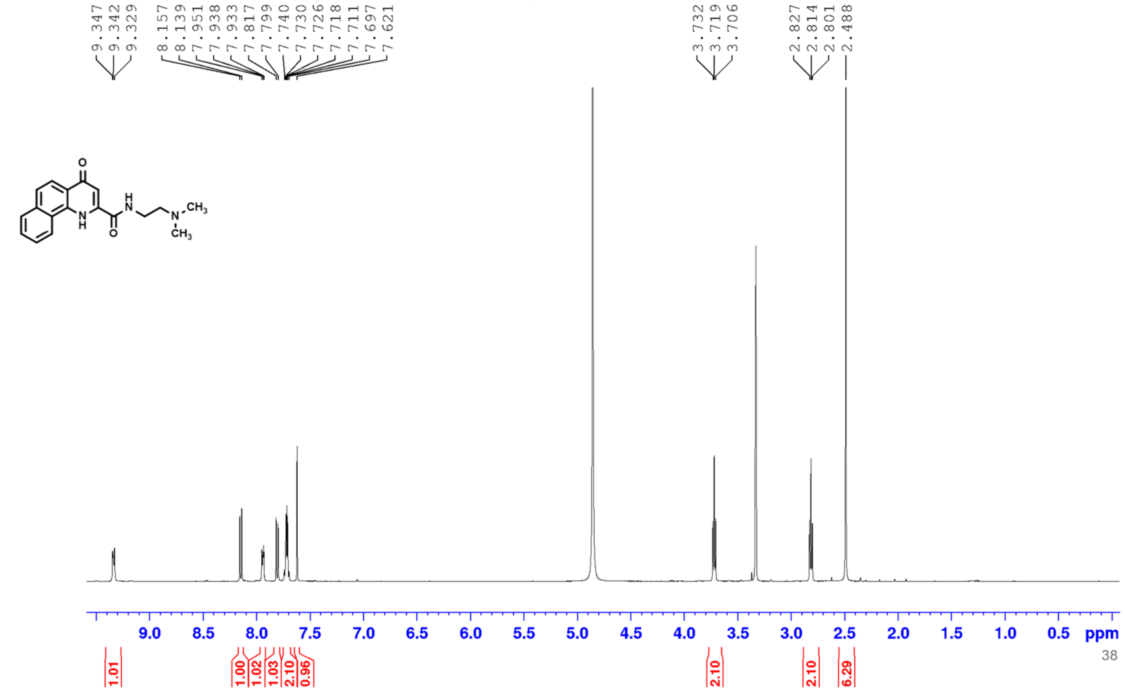

Figure S38. ^{1}H-NMR spectrum of **13**

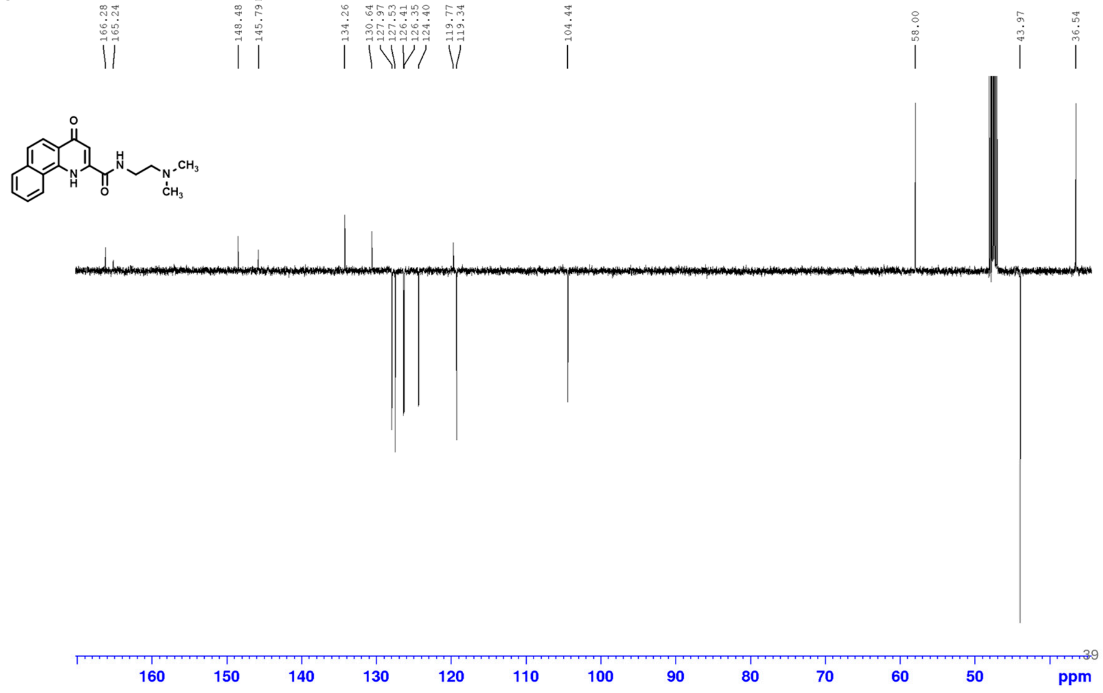

Figure S39. ^{13}C-NMR spectrum of **13**

N-(2-(dimethylamino)ethyl)-4-oxo-1,4-dihydro-1,10-phenanthroline-2-carboxamide (**14**)

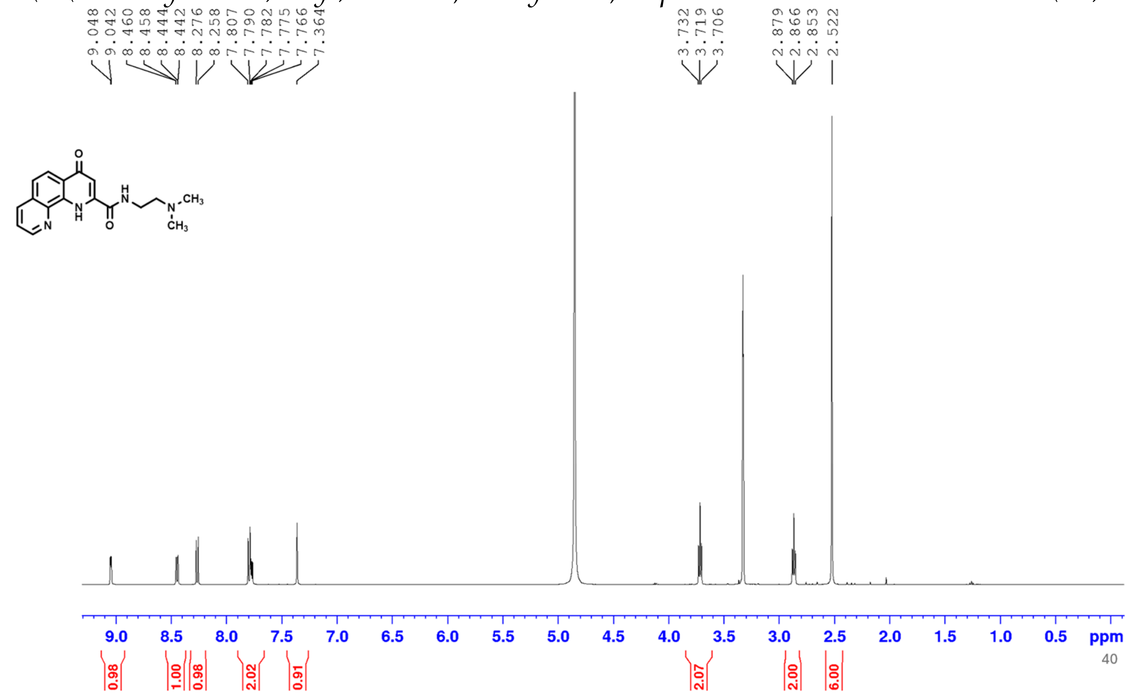

Figure S40. ^{1}H-NMR spectrum of **14**

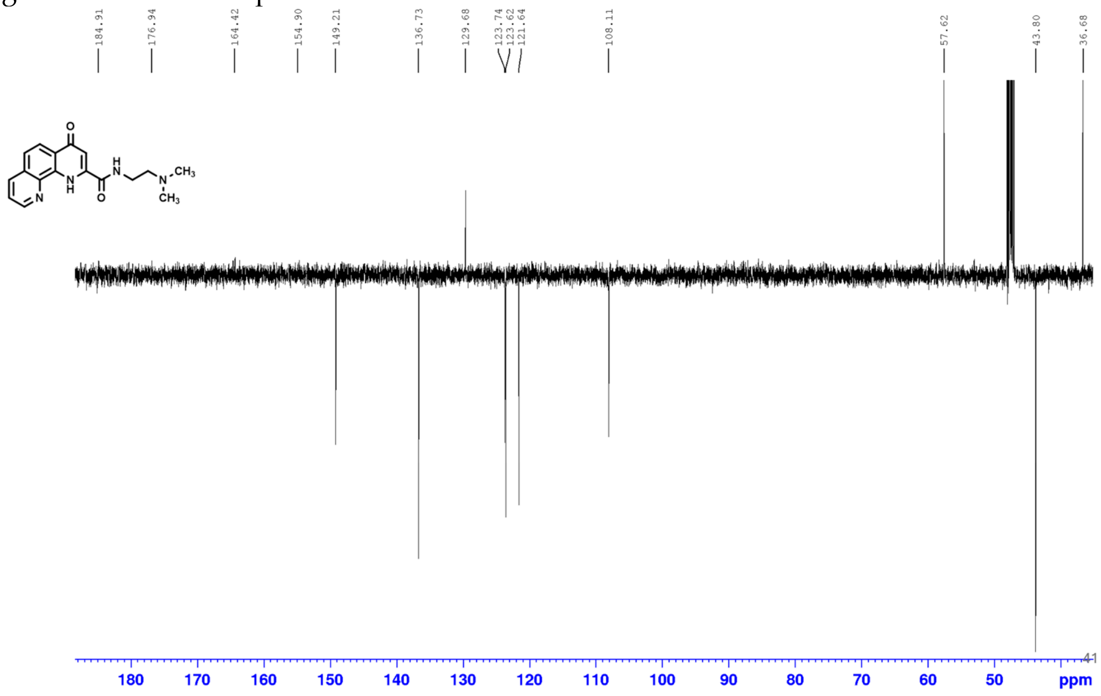

Figure S41. ^{13}C-NMR spectrum of **14**

N-(2-(dimethylamino)ethyl)-6-oxo-6,9-dihydro-1H-pyrrolo[3,2-h]quinoline-8-carboxamide (**15**)

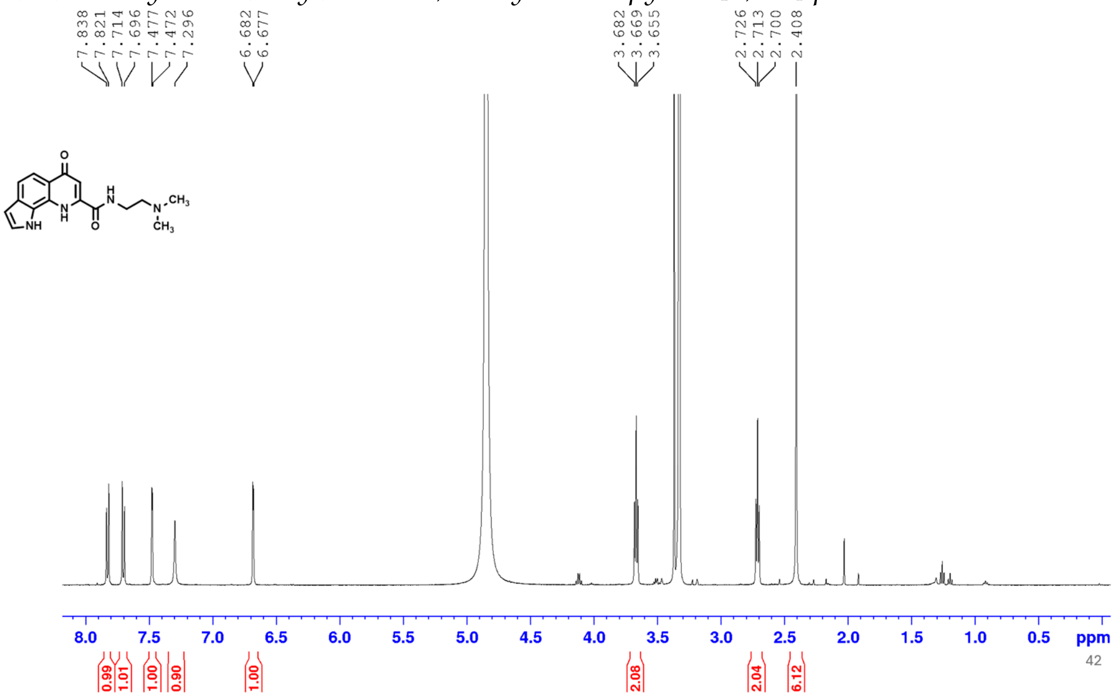

Figure S42. ^{1}H-NMR spectrum of **15**

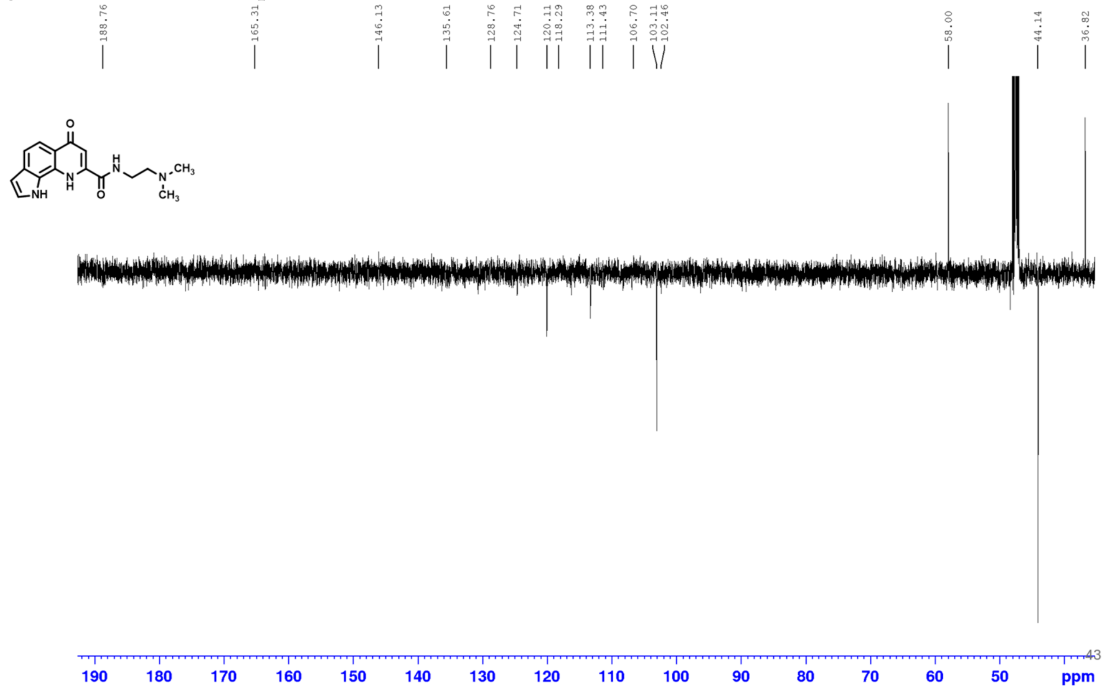

Figure S43. ^{13}C-NMR spectrum of **15**

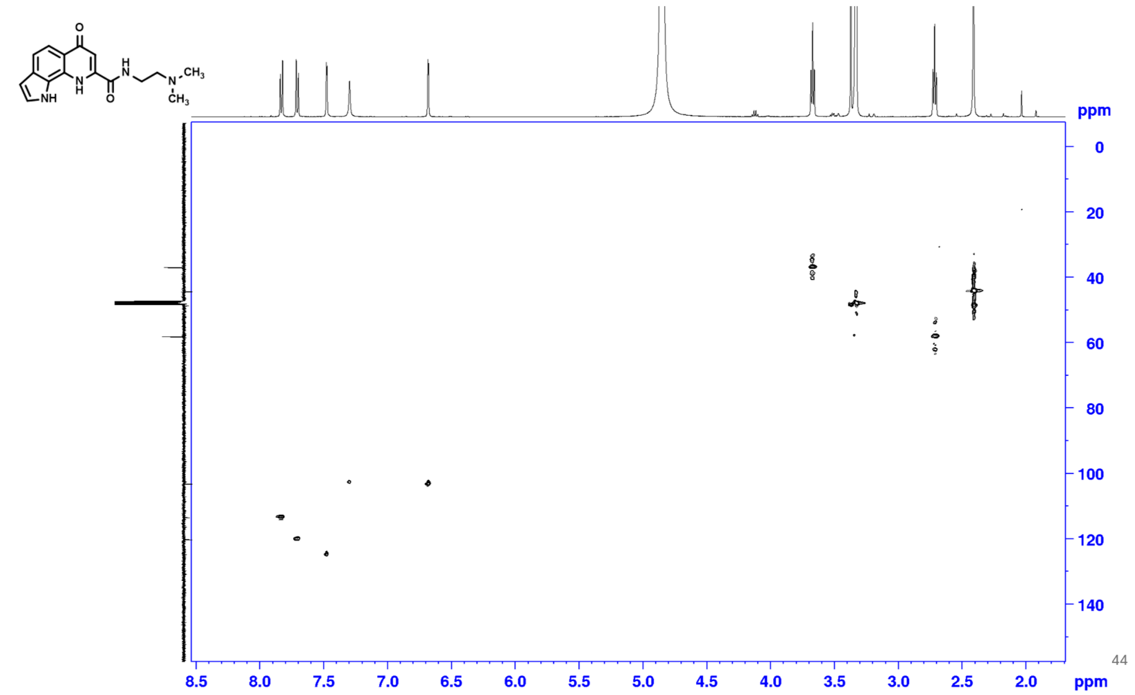

Figure S44. HSQC NMR spectrum of **15**

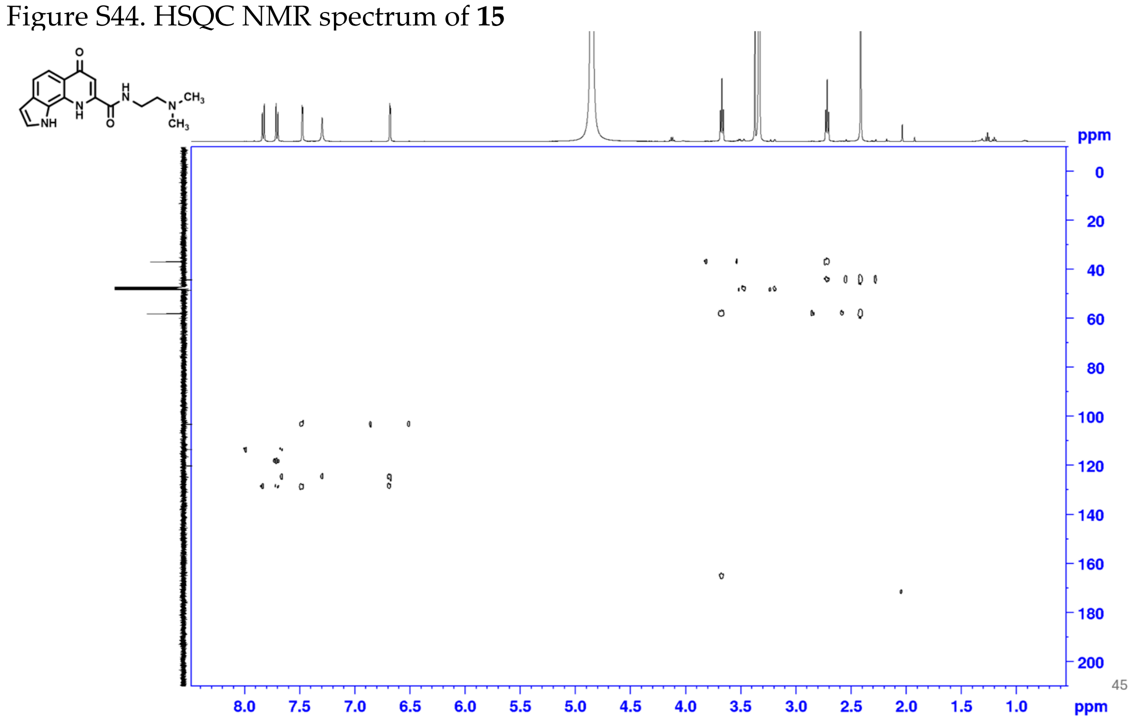

Figure S45. HMBC NMR spectrum of **15**

N-(2-(dimethylamino)ethyl)-3-(morpholinomethyl)-4-oxo-1,4-dihydrobenzo[h]quinoline-2-carboxamide (**16a**)

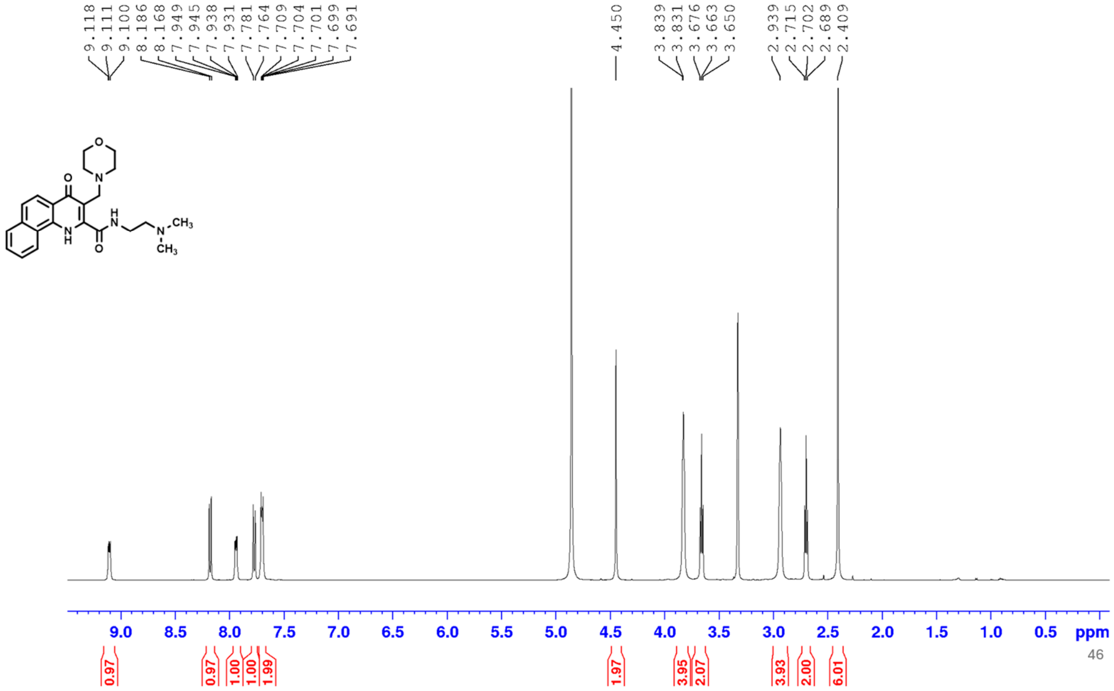

Figure S46. ^{1}H-NMR spectrum of **16a**

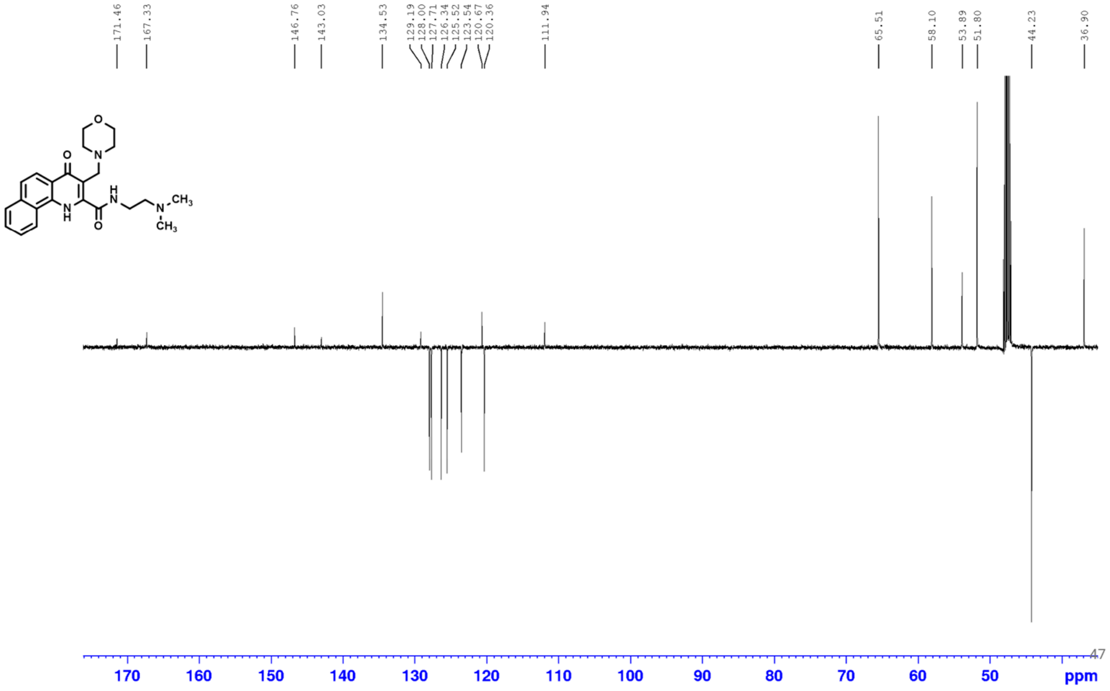

Figure S47. ^{13}C-NMR spectrum of **16a**

N-(2-(dimethylamino)ethyl)-4-oxo-3-(piperidin-1-ylmethyl)-1,4-dihydrobenzo[h]quinoline-2-carboxamide (**16b**)

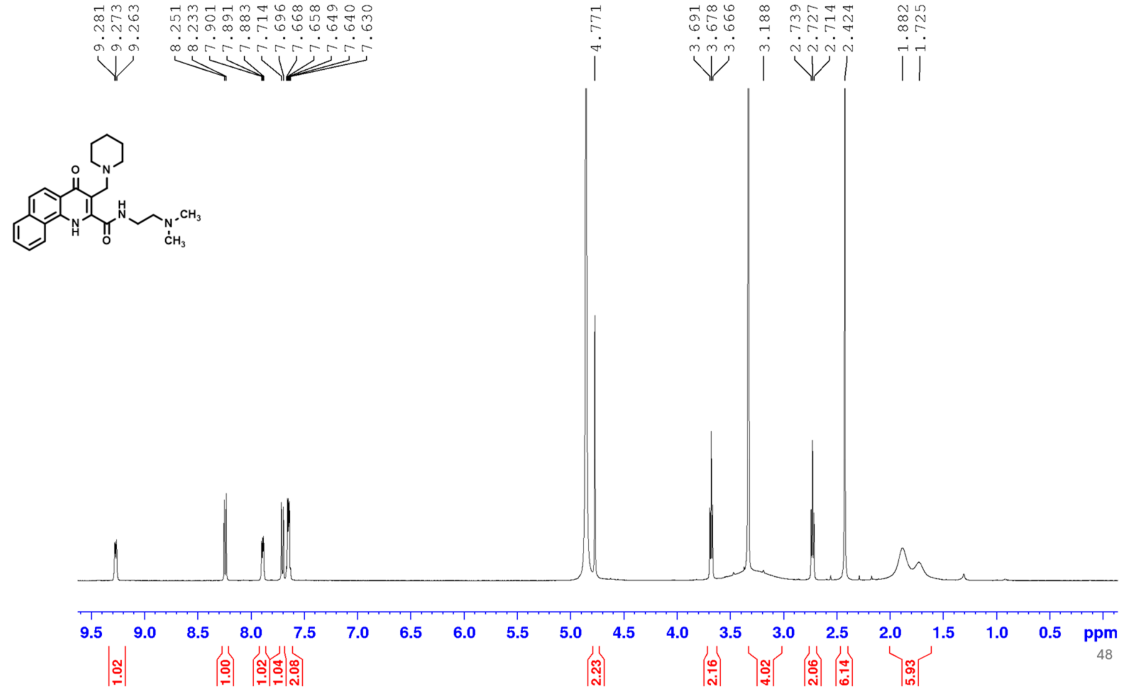

Figure S48. ^{1}H-NMR spectrum of **16b**

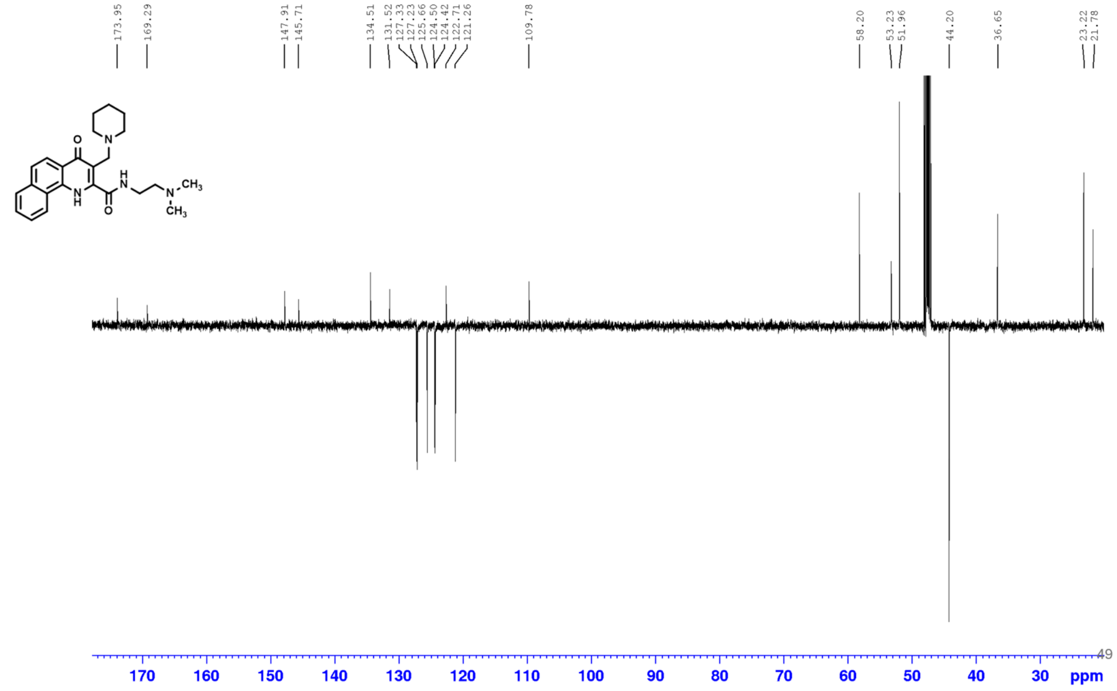

Figure S49. ^{13}C-NMR spectrum of **16b**

*N-(2-(dimethylamino)ethyl)-4-oxo-3-(pyrrolidin-1-ylmethyl)-1,4-dihydrobenzo[h]quinoline-2-carboxamide (**16c**)*

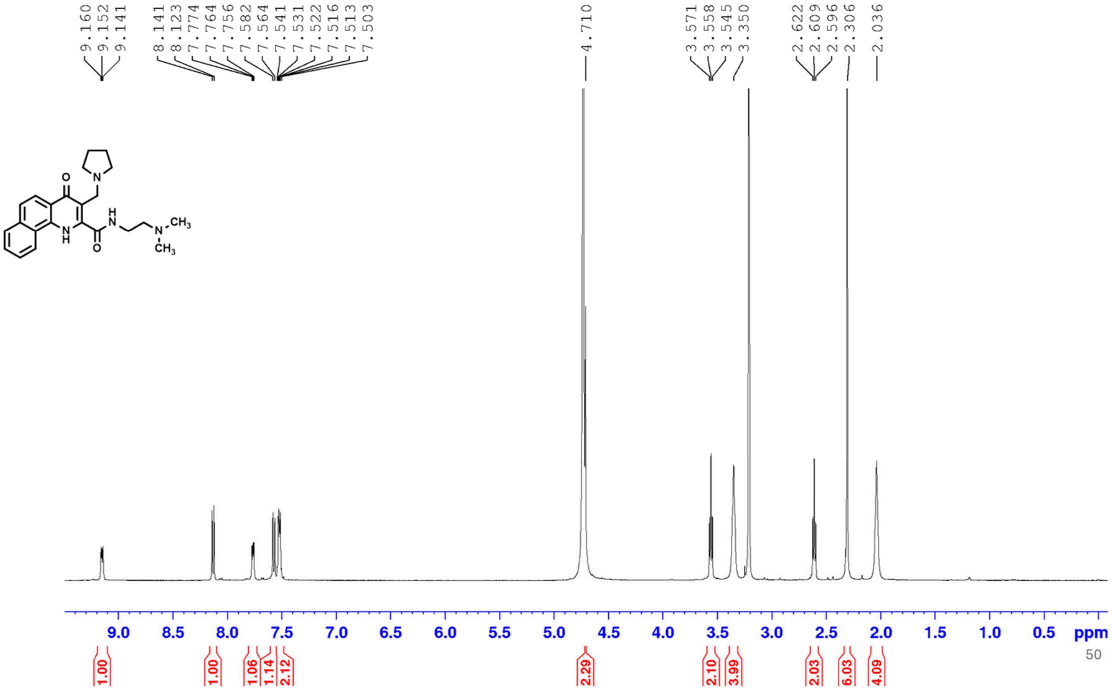

Figure S50. ^{1}H-NMR spectrum of **16c**

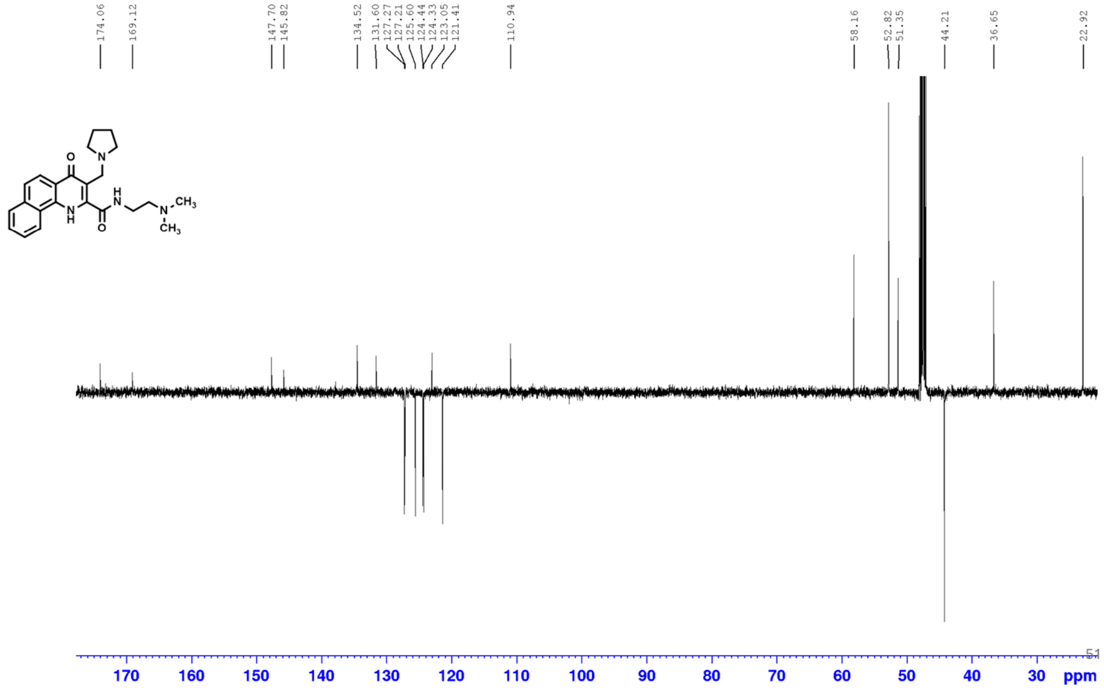

Figure S51. ^{13}C-NMR spectrum of **16c**

N-(2-(dimethylamino)ethyl)-3-((4-methylpiperazin-1-yl)methyl)-4-oxo-1,4-dihydrobenzo[h]quinoline-2-carboxamide (**16d**)

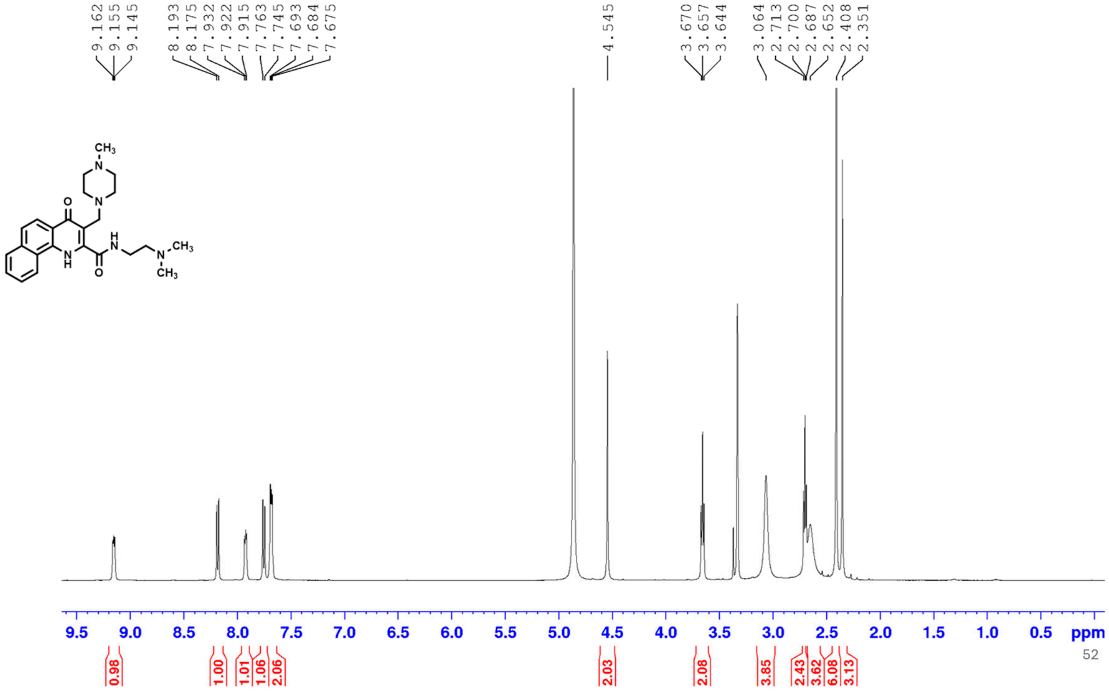

Figure S52. ^{1}H-NMR spectrum of **16d**

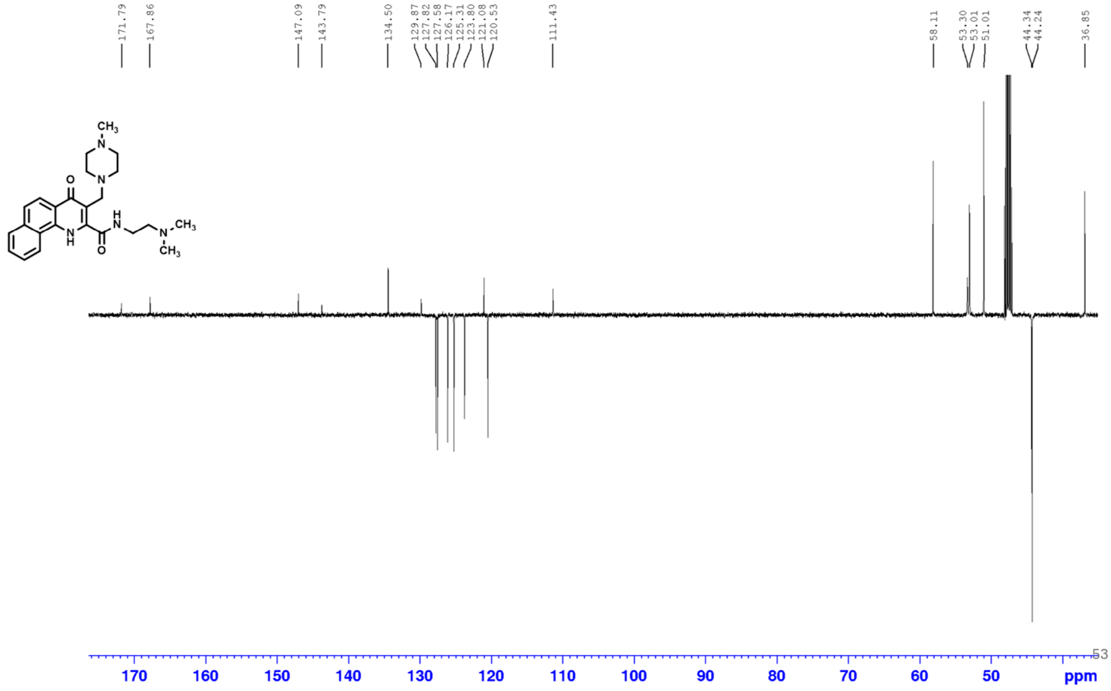

Figure S53. ^{13}C-NMR spectrum of **16d**

N-(2-(dimethylamino)ethyl)-3-(morpholinomethyl)-4-oxo-1,4-dihydro-1,10-phenanthroline-2-carboxamide (**17a**)

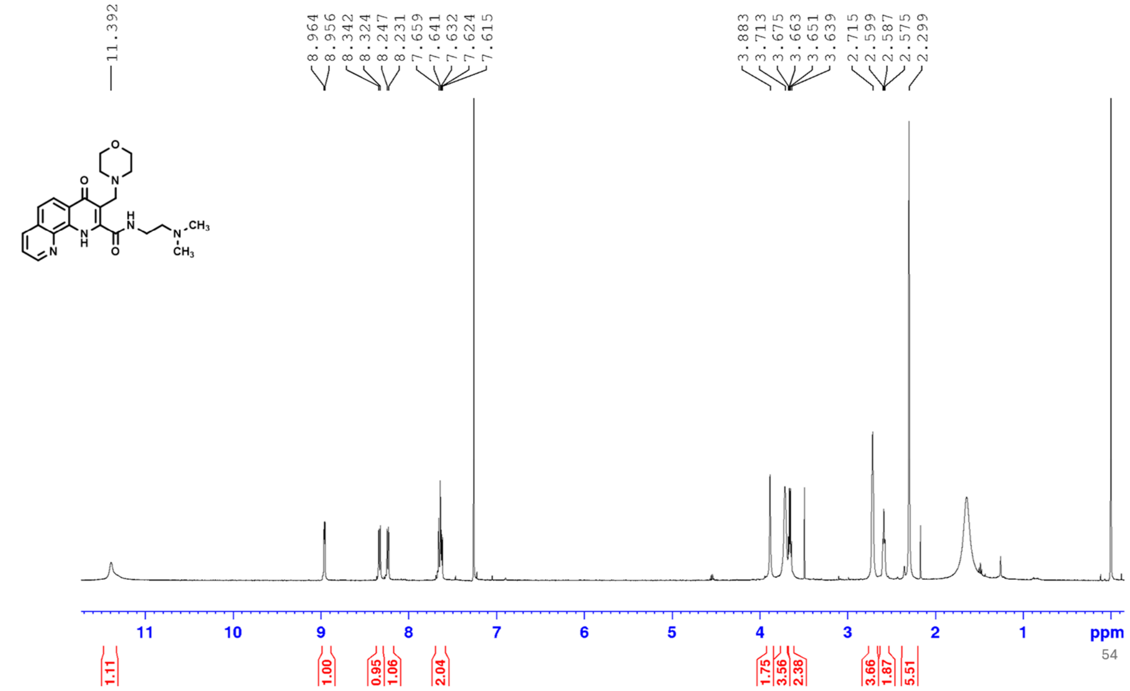

Figure S54. ^{1}H-NMR spectrum of **17a**

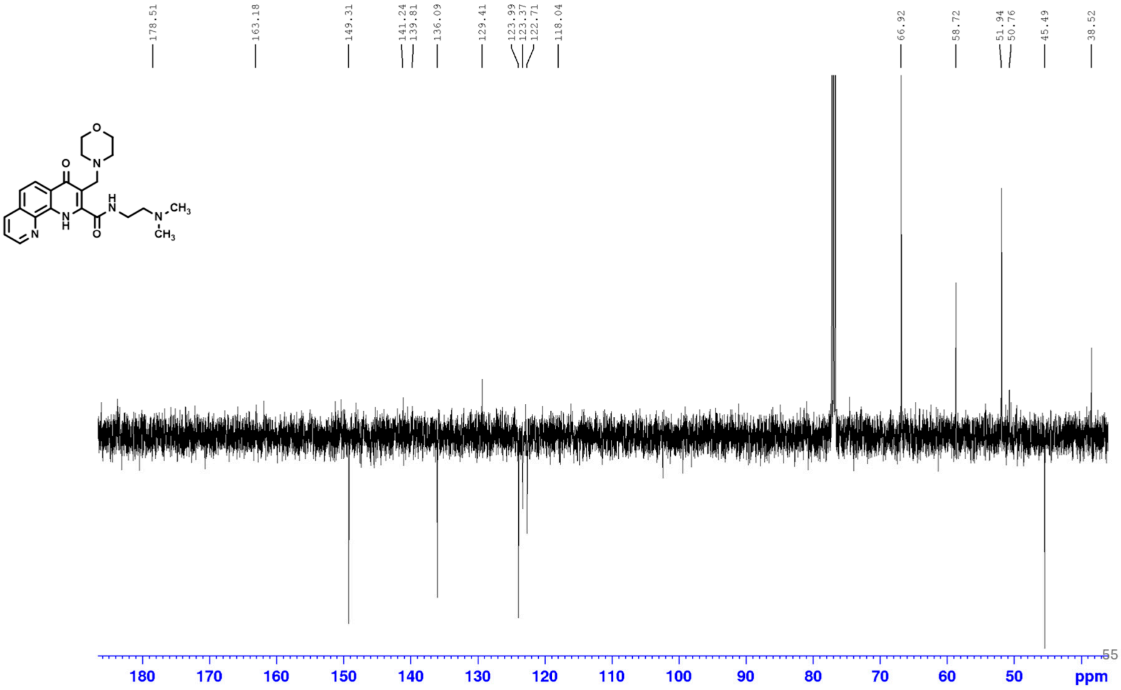

Figure S55. ^{13}C-NMR spectrum of **17a**

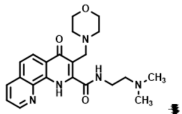

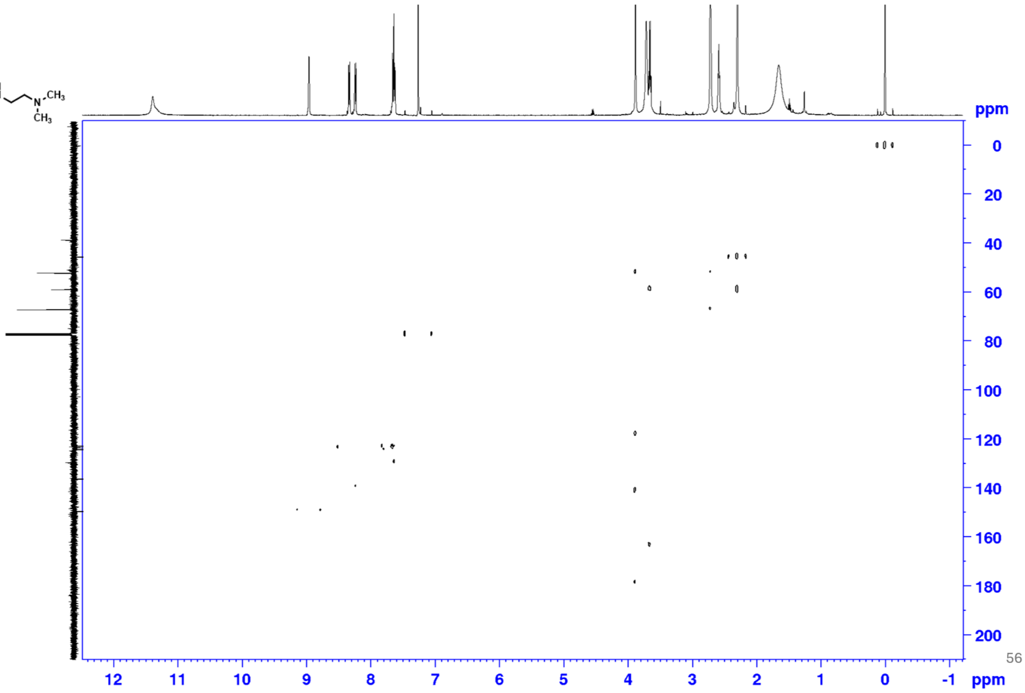

Figure S56. HMBC NMR spectrum of **17a**

*N-(2-(dimethylamino)ethyl)-4-oxo-3-(piperidin-1-ylmethyl)-1,4-dihydro-1,10-phenanthroline-2-carboxamide (**17b**)*

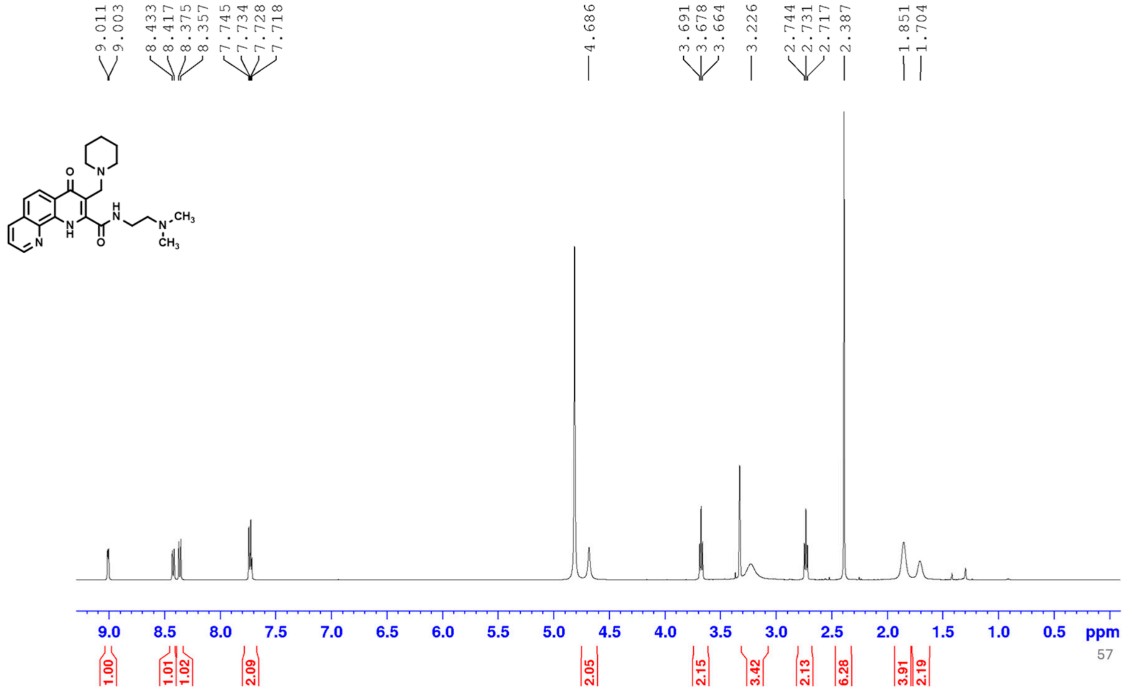

Figure S57. ^{1}H-NMR spectrum of **17b**

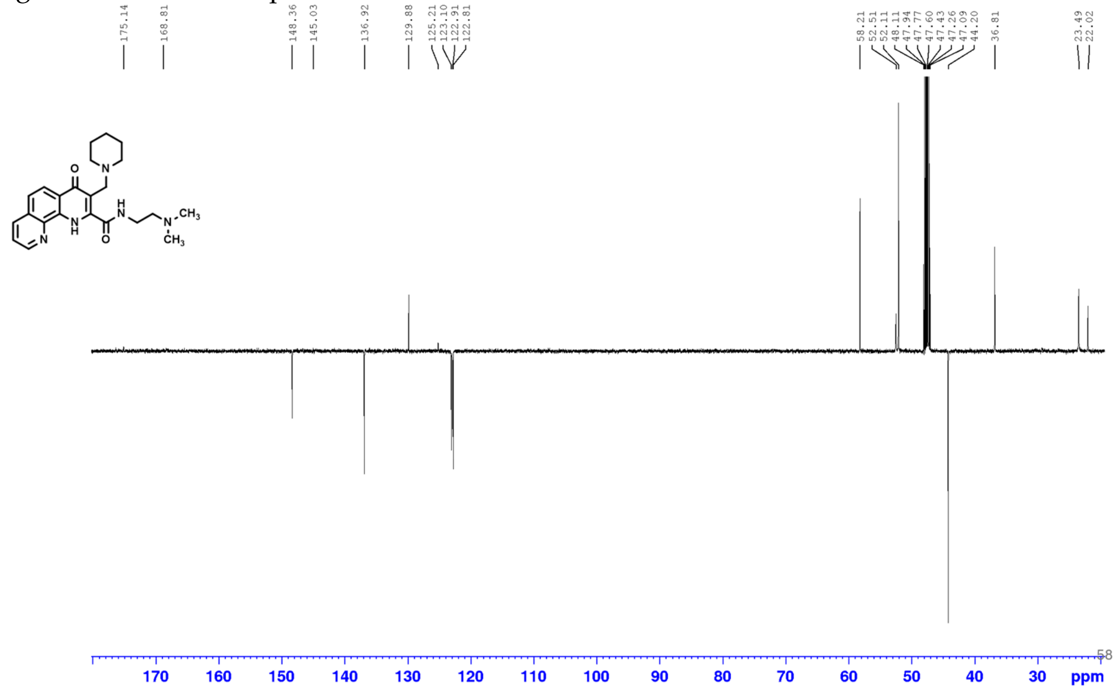

Figure S58. ^{13}C-NMR spectrum of **17b**

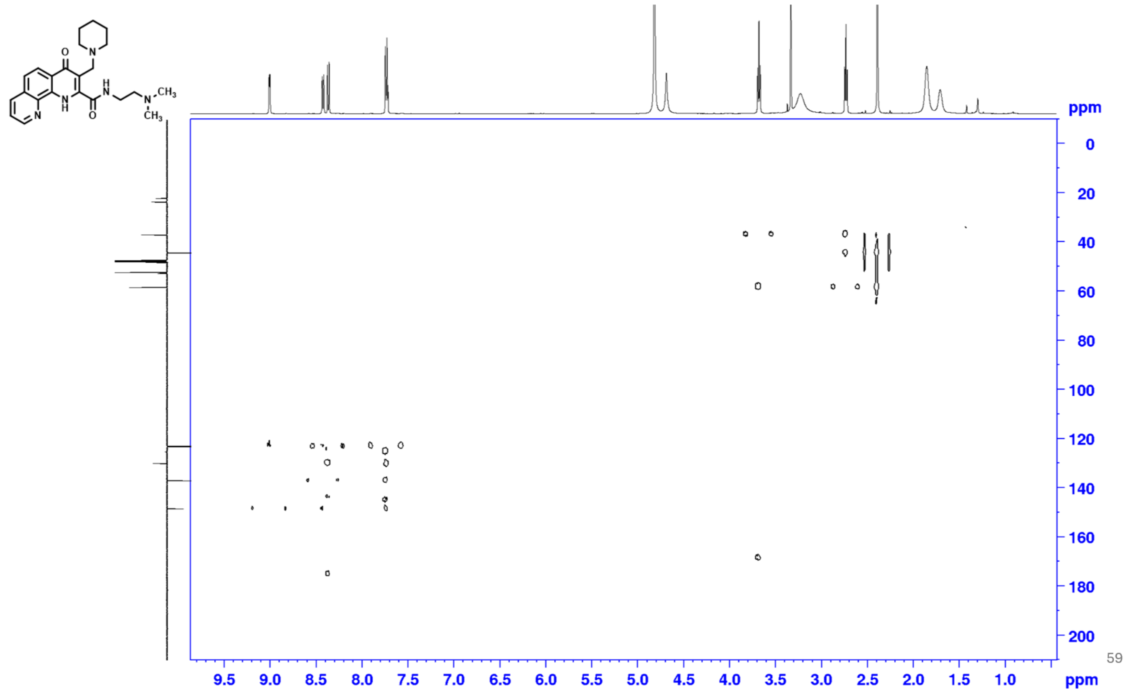

Figure S59. HMBC NMR spectrum of **17b**

N-(2-(dimethylamino)ethyl)-4-oxo-3-(pyrrolidin-1-ylmethyl)-1,4-dihydro-1,10-phenanthroline-2-carboxamide (**17c**)

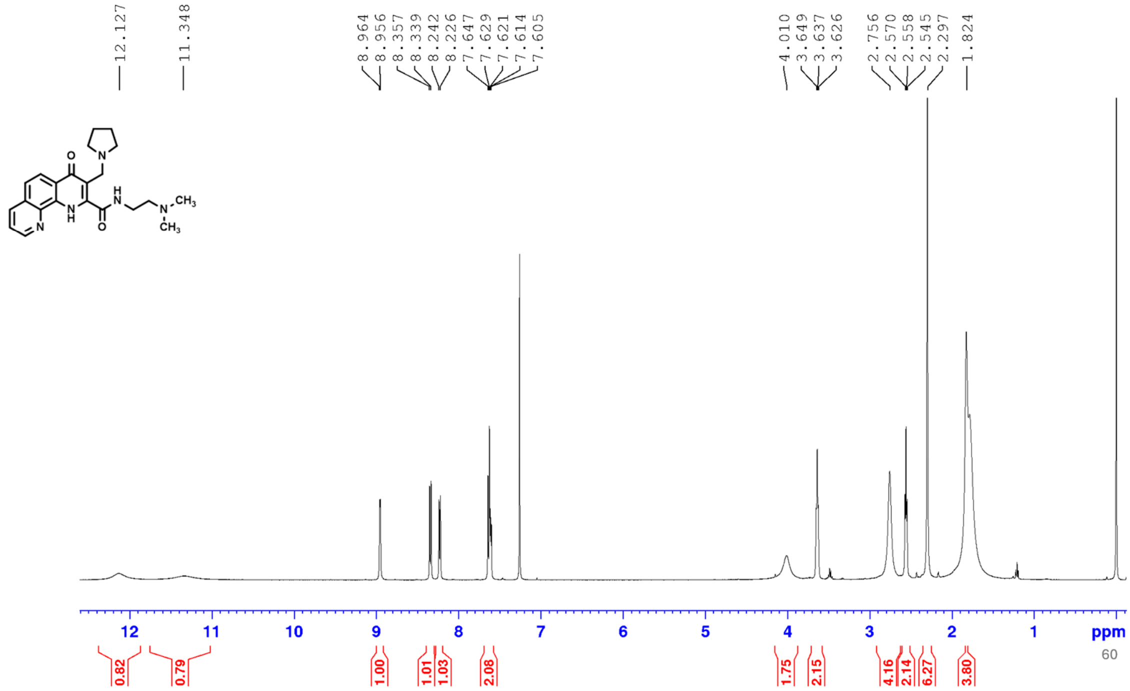

Figure S60. ^{1}H-NMR spectrum of **17c**

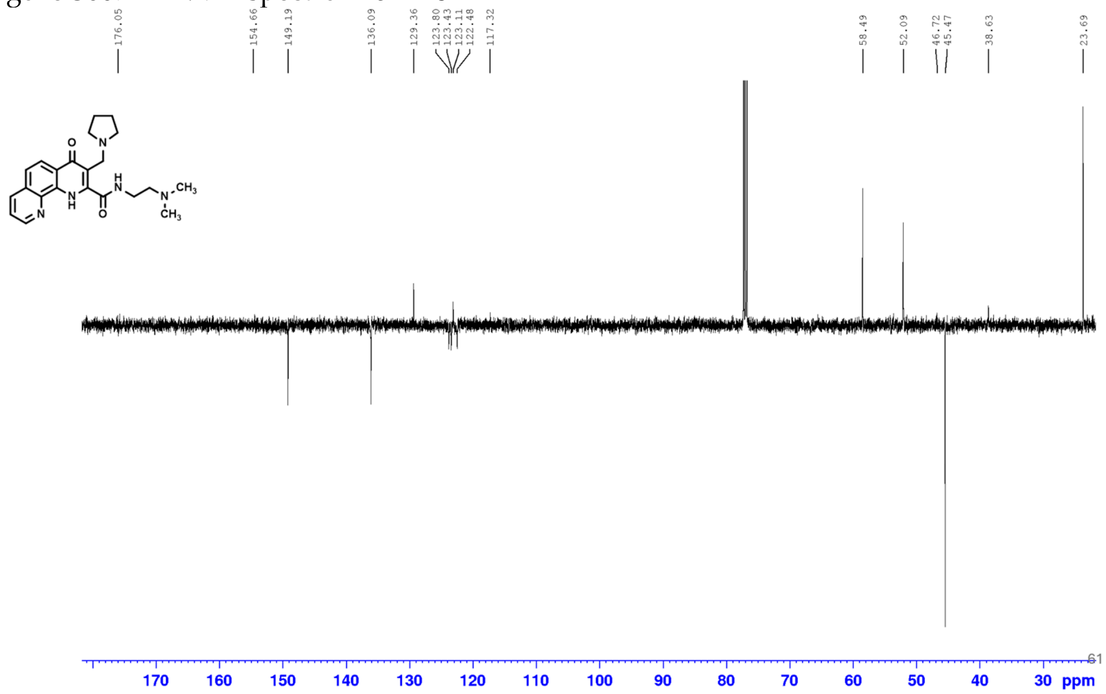

Figure S61. ^{13}C-NMR spectrum of **17c**

N-(2-(dimethylamino)ethyl)-3-((4-methylpiperazin-1-yl)methyl)-4-oxo-1,4-dihydro-1,10-phenanthroline-2-carboxamide (**17d**)

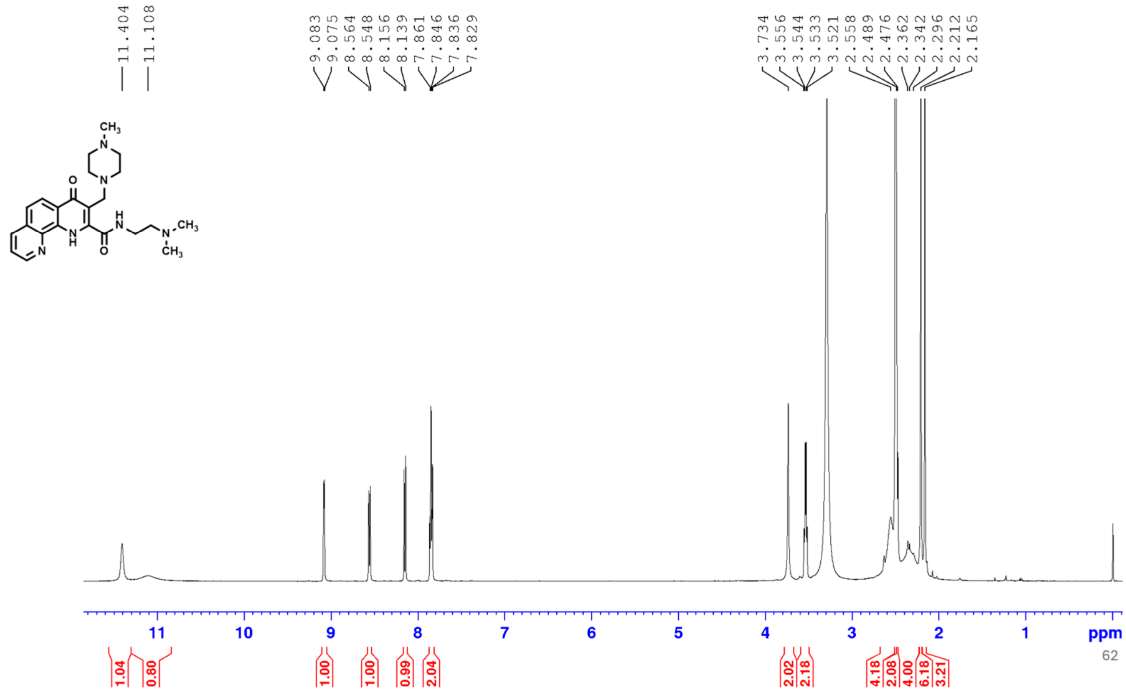

Figure S62. ^{1}H-NMR spectrum of **17d**

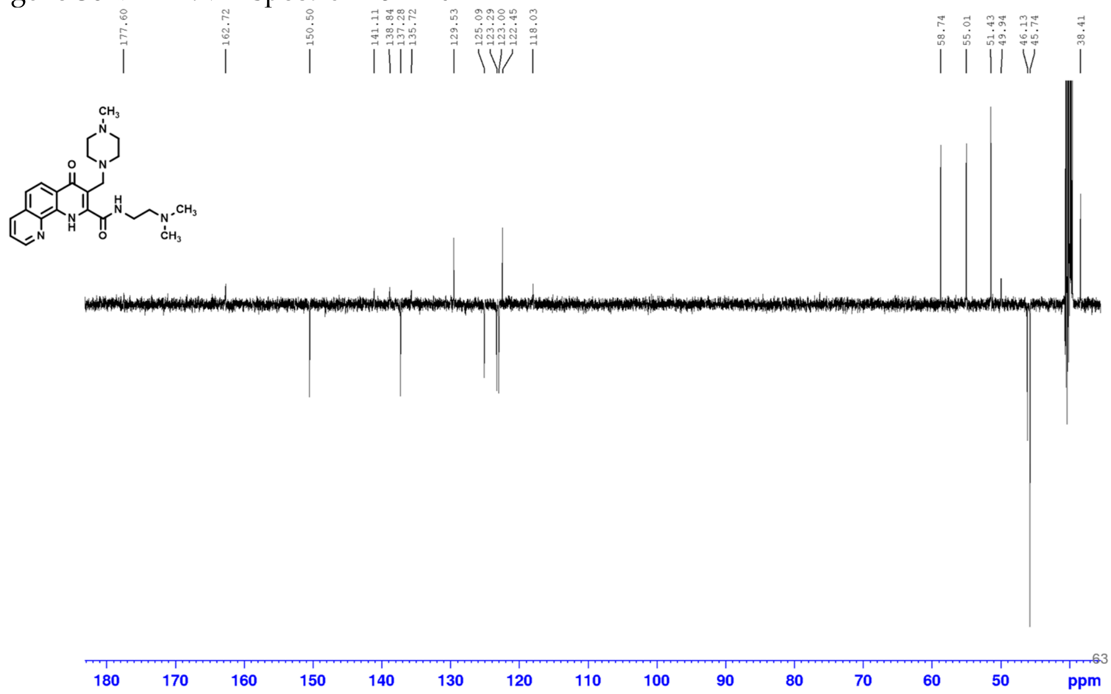

Figure S63. ^{13}C-NMR spectrum of **17d**

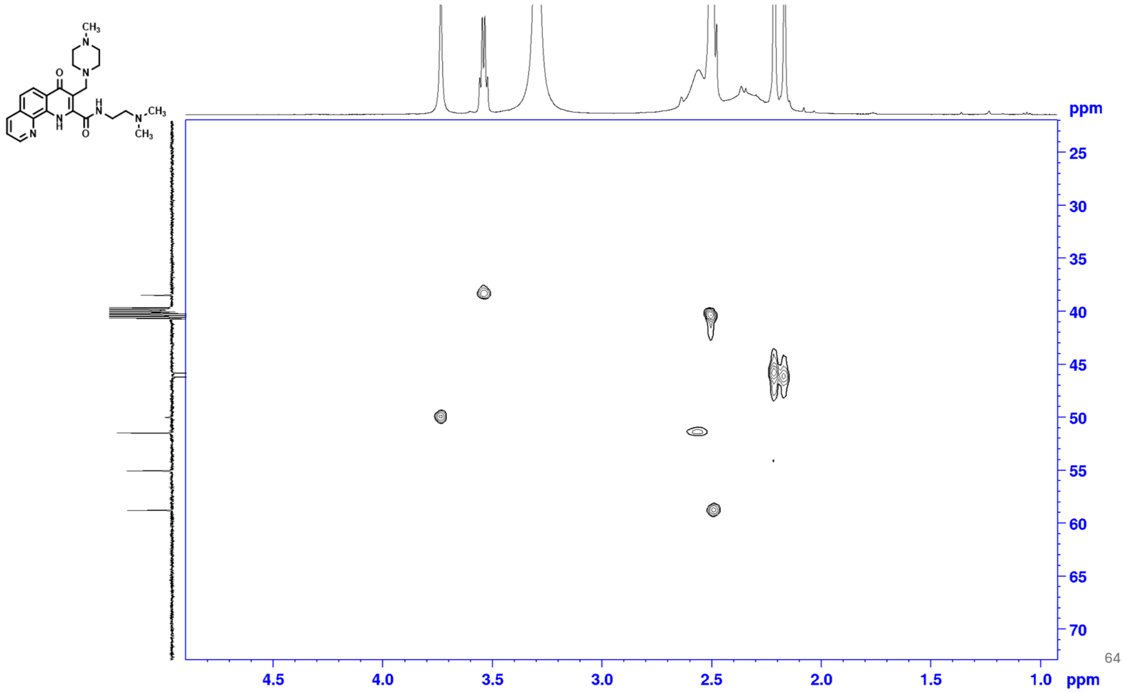

Figure S64. HSQC NMR spectrum of **17d**

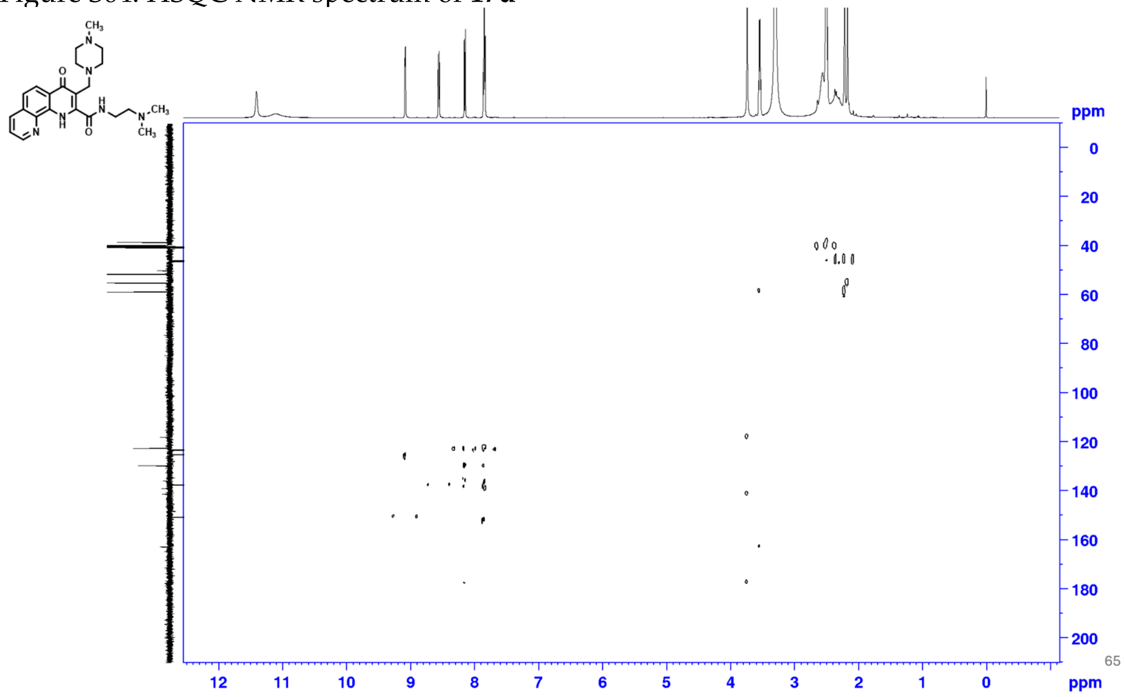

Figure S65. HMBC NMR spectrum of **17d**

N-(2-(dimethylamino)ethyl)-7-(morpholinomethyl)-6-oxo-6,9-dihydro-1H-pyrrolo[3,2-h]quinoline-8-carboxamide (**18a**)

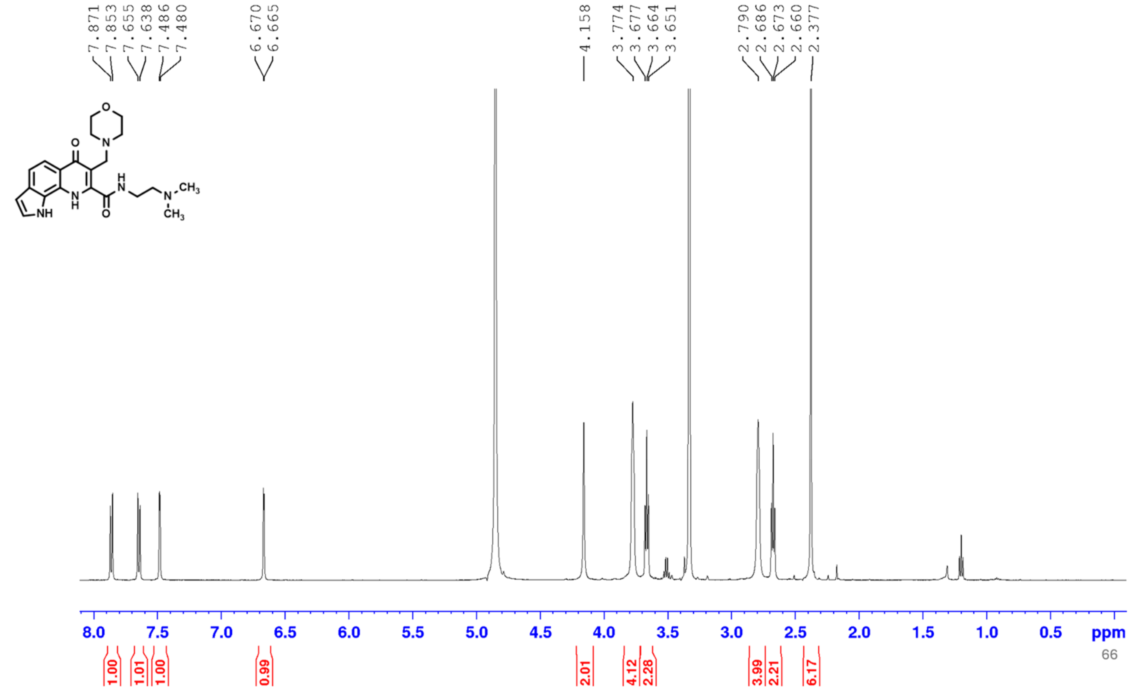

Figure S66. ^{1}H-NMR spectrum of **18a**

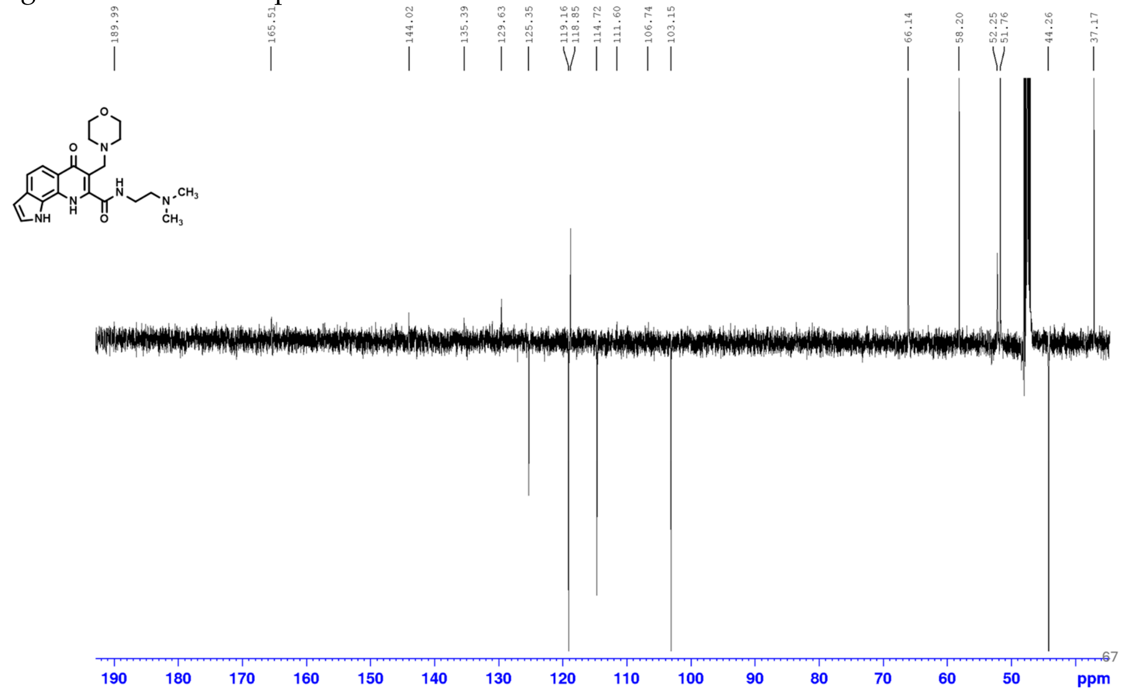

Figure S67. ^{13}C-NMR spectrum of **18a**

N-(2-(dimethylamino)ethyl)-6-oxo-7-(piperidin-1-ylmethyl)-6,9-dihydro-1H-pyrrolo[3,2-h]quinoline-8-carboxamide (**18b**)

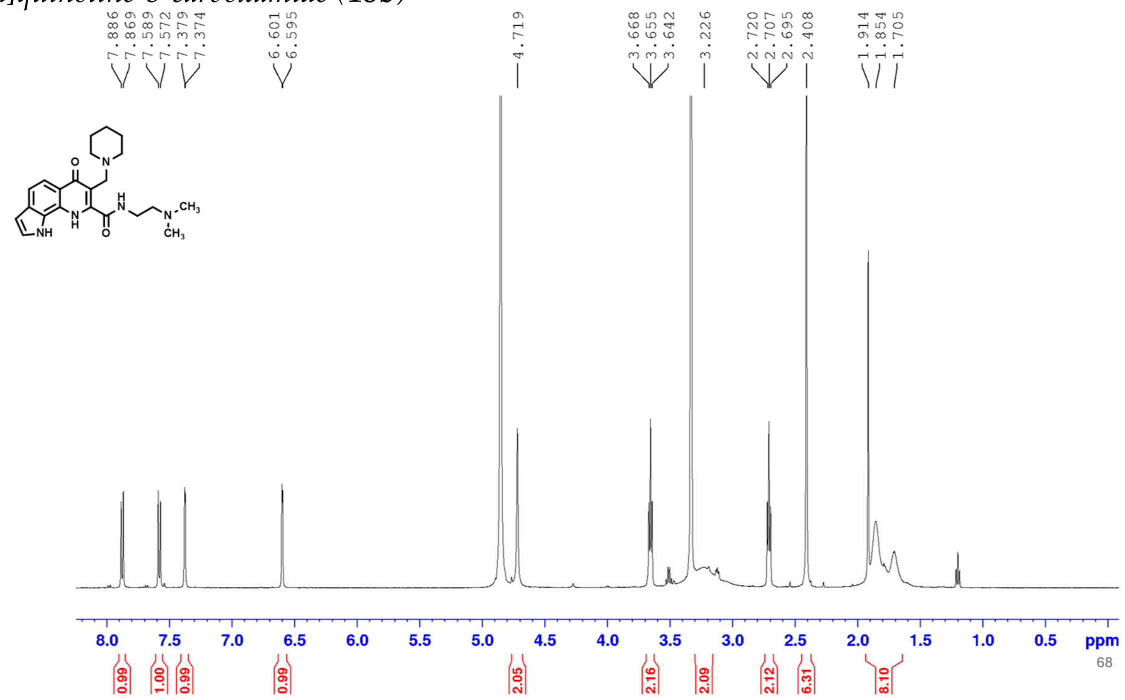

Figure S68. ^{1}H-NMR spectrum of **18b**

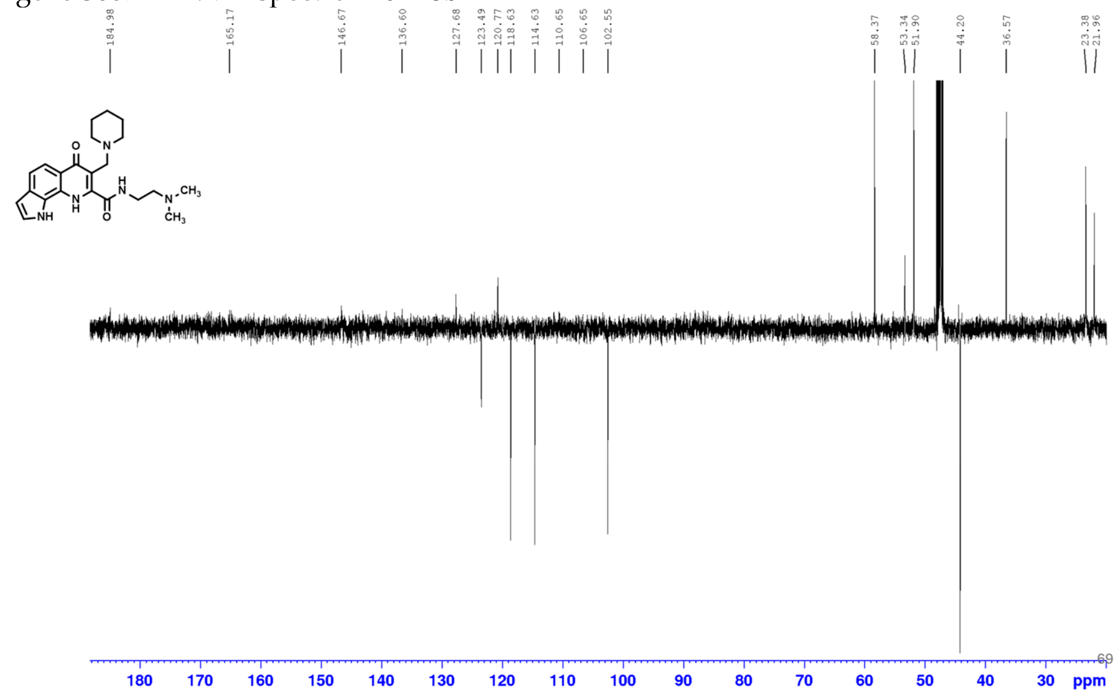

Figure S69. ^{13}C-NMR spectrum of **18b**

N-(2-(dimethylamino)ethyl)-6-oxo-7-(pyrrolidin-1-ylmethyl)-6,9-dihydro-1H-pyrrolo[3,2-h]quinoline-8-carboxamide (**18c**)

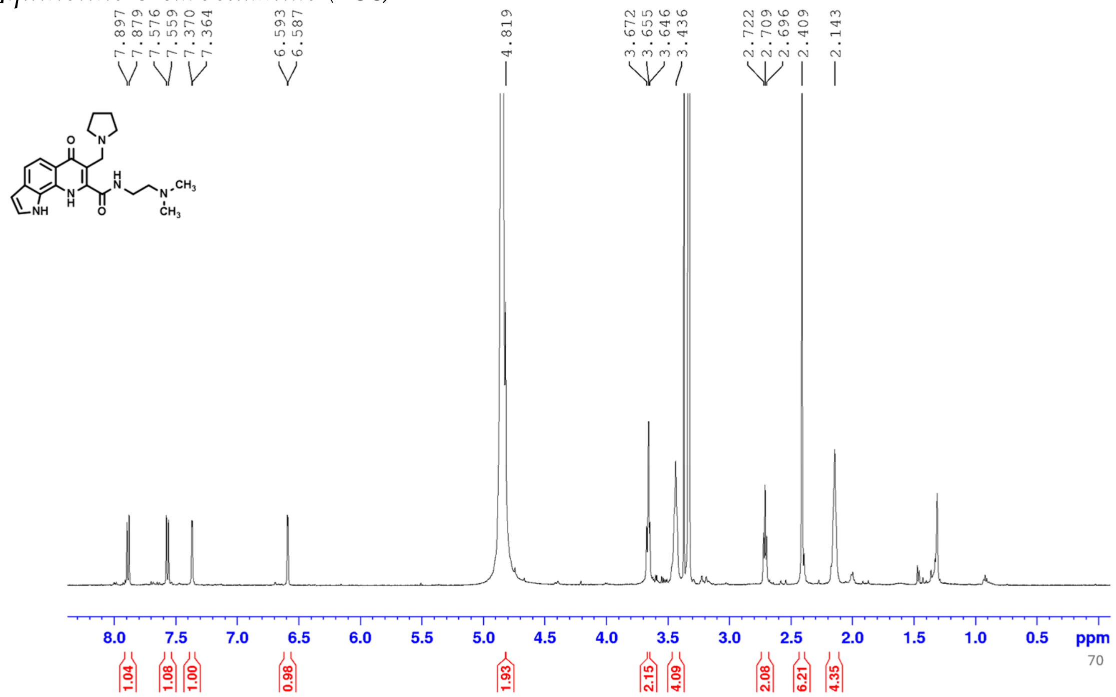

Figure S70. ^{1}H-NMR spectrum of **18c**

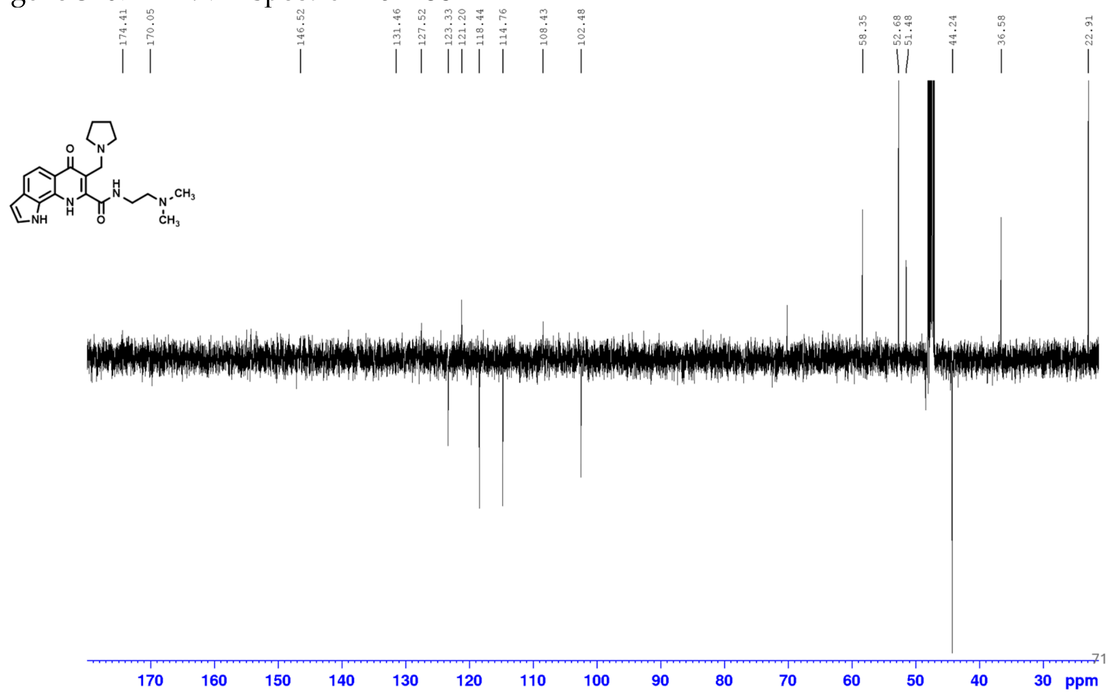

Figure S71. ^{13}C-NMR spectrum of **18c**

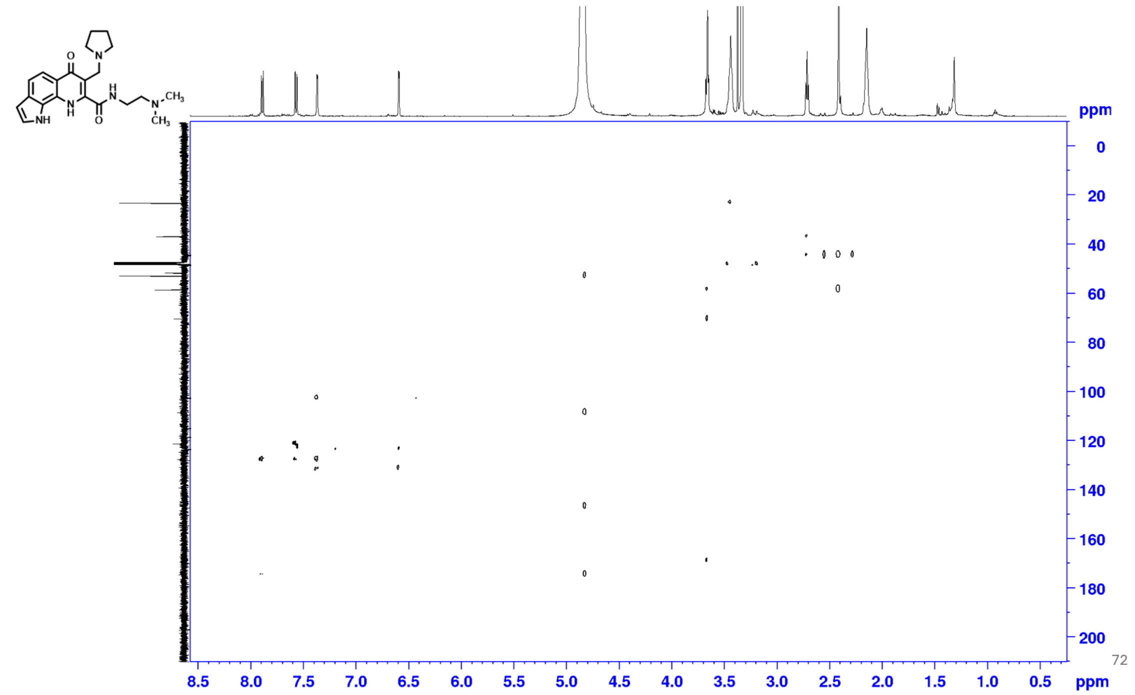

Figure S72. HMBC NMR spectrum of **18c**

N-(2-(dimethylamino)ethyl)-7-((4-methylpiperazin-1-yl)methyl)-6-oxo-6,9-dihydro-1H-pyrrolo[3,2-h]quinoline-8-carboxamide (**18d**)

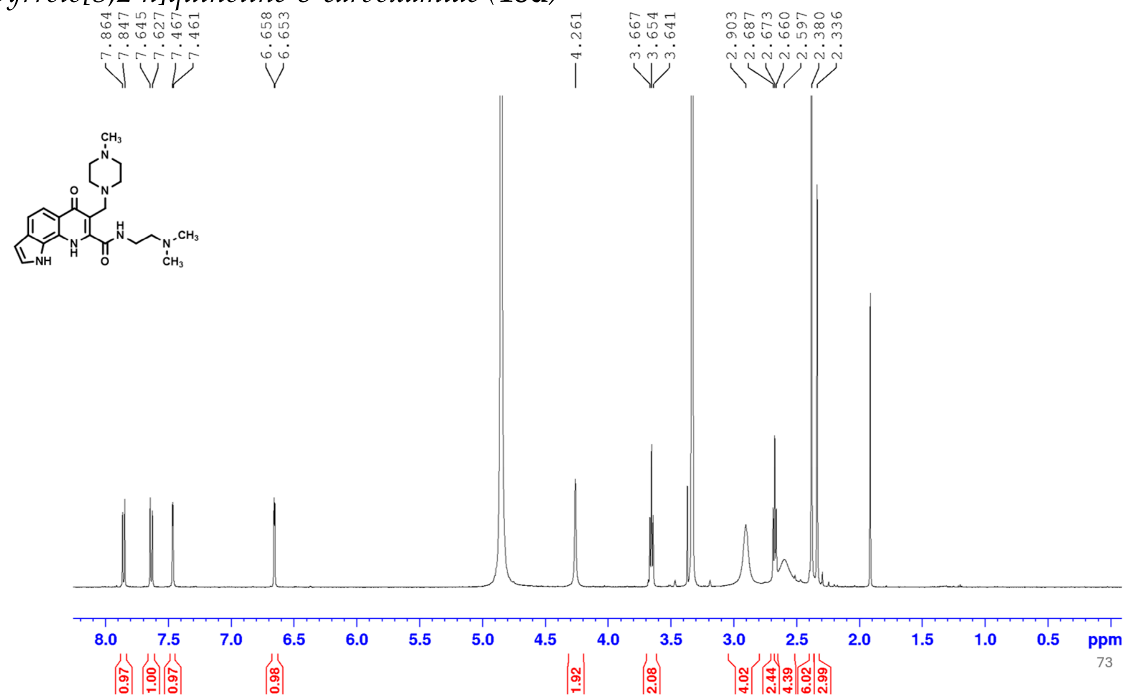

Figure S73. ^{1}H-NMR spectrum of **18d**

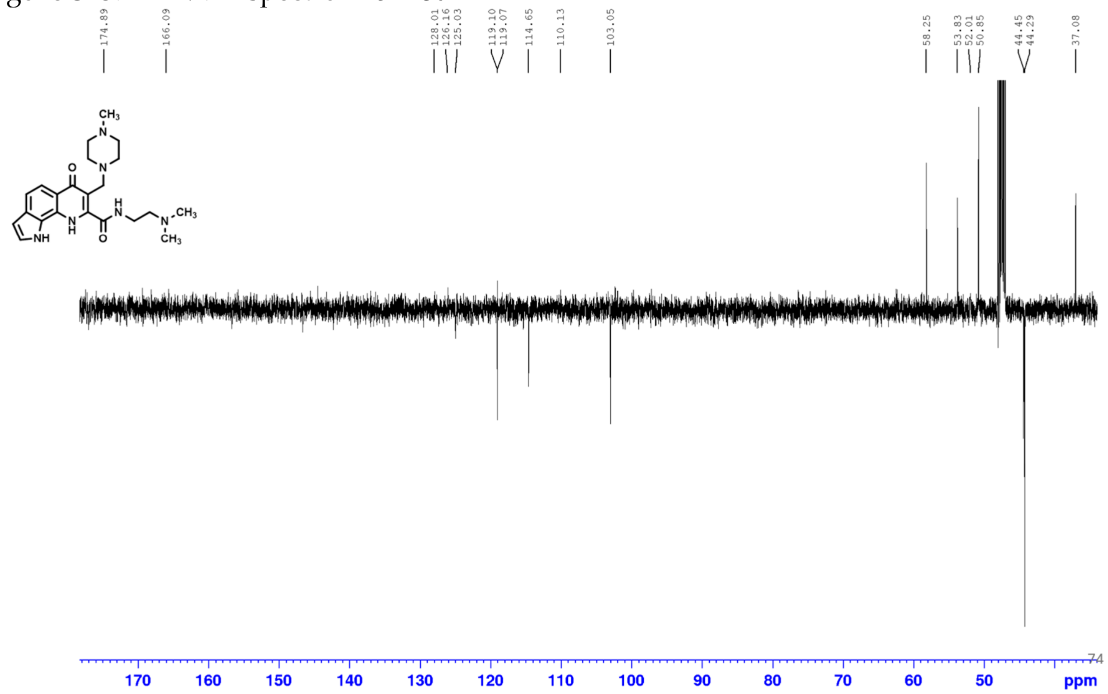

Figure S74. ^{13}C-NMR spectrum of **18d**

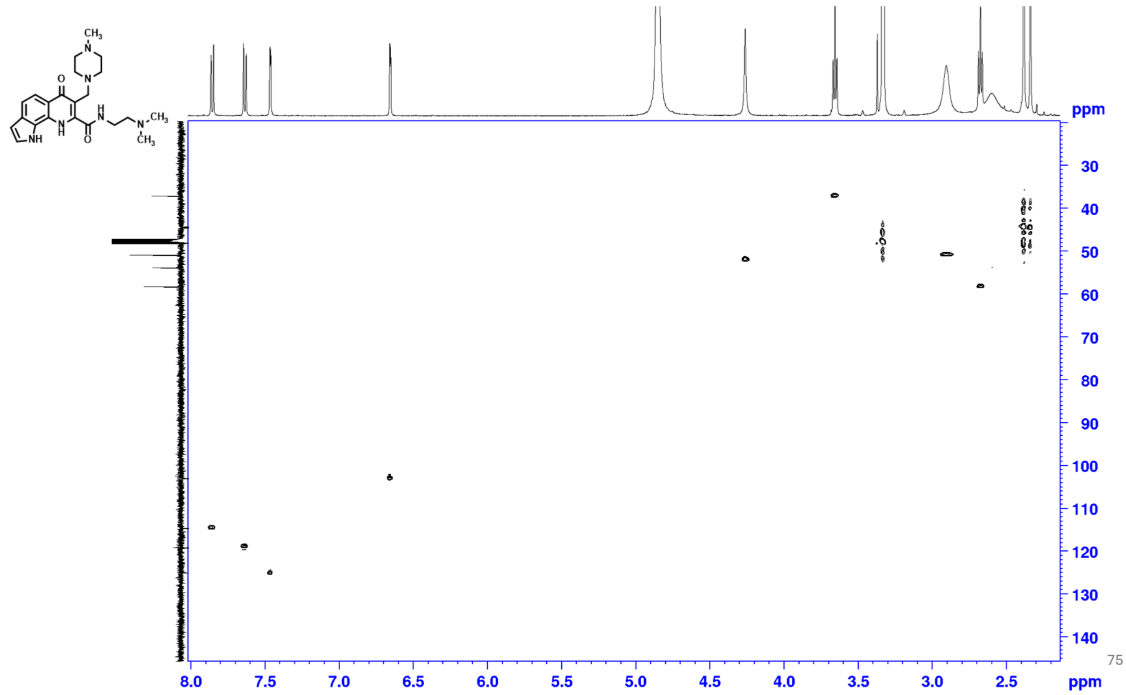

Figure S75. HSQC NMR spectrum of **18d**

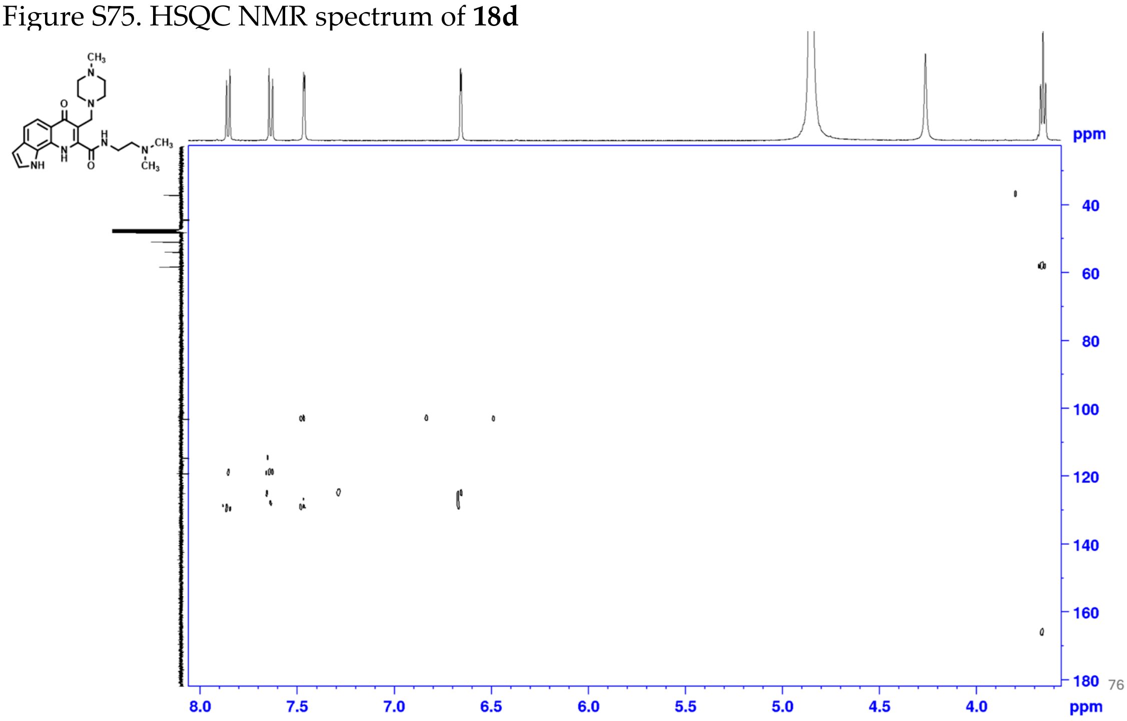

Figure S76. HMBC NMR spectrum of **18d**

Diethyl 2-(naphthalen-1-ylamino)fumarate (**4**)

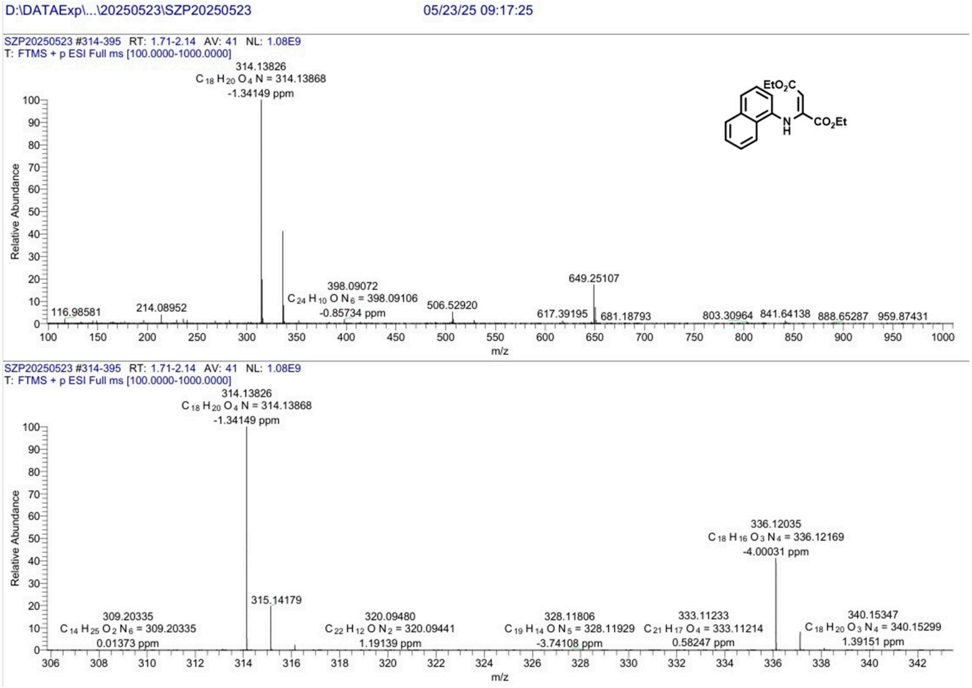

Figure S77. HR-MS spectrum of **4**

*Diethyl 2-(quinolin-8-ylamino)fumarate (**5**)*

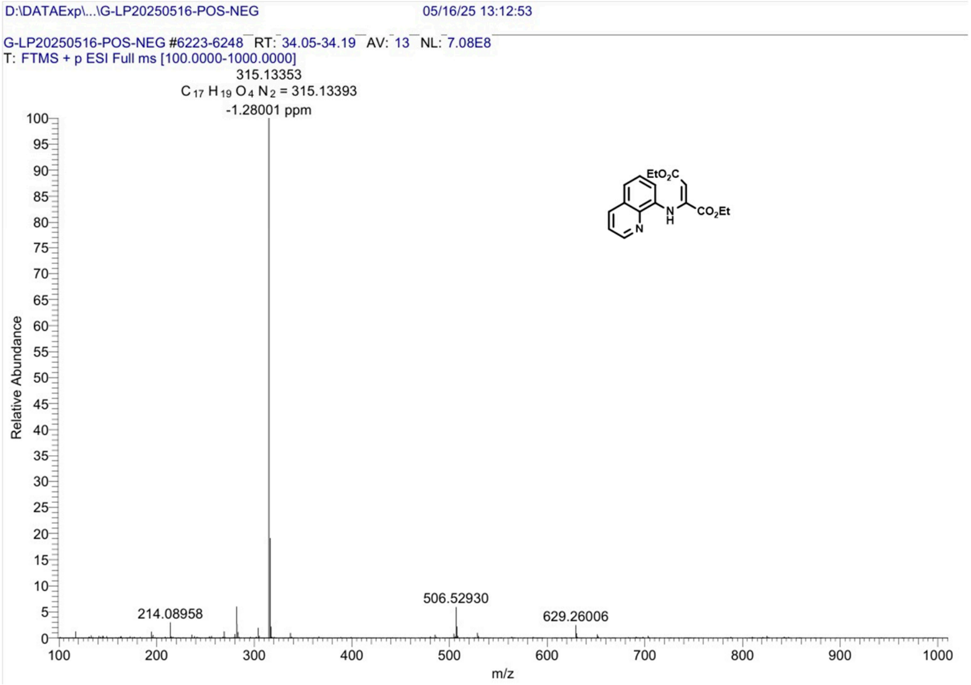

Figure S78. HR-MS spectrum of **5**

*Ethyl 4-oxo-1,4-dihydrobenzo[h]quinoline-2-carboxylate (**7**)*

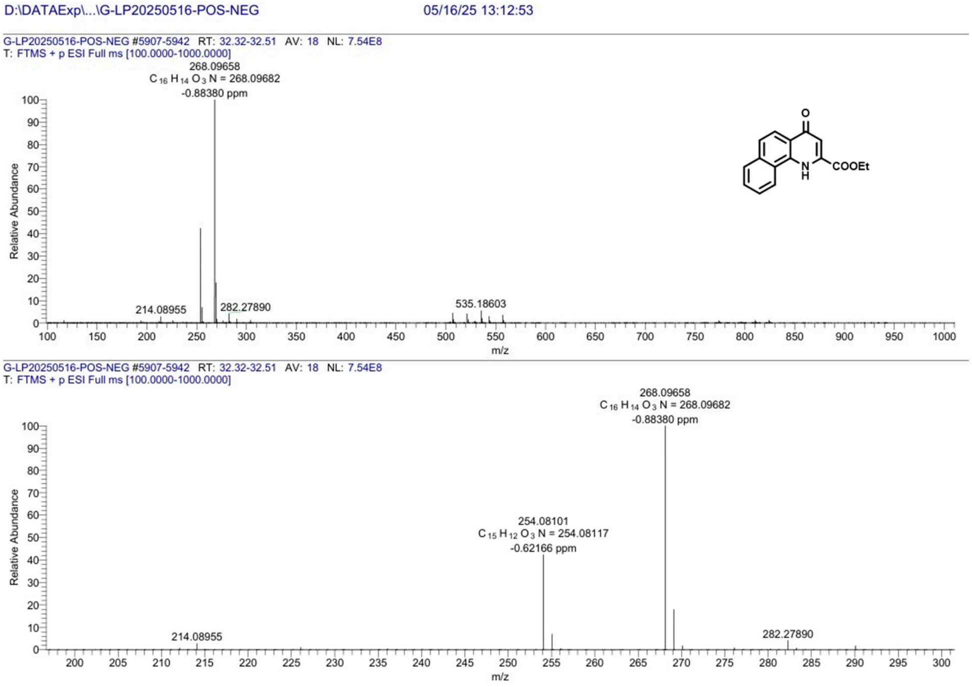

Figure S79. HR-MS spectrum of **7**

Ethyl 4-oxo-1,4-dihydro-1,10-phenanthroline-2-carboxylate (**8**)

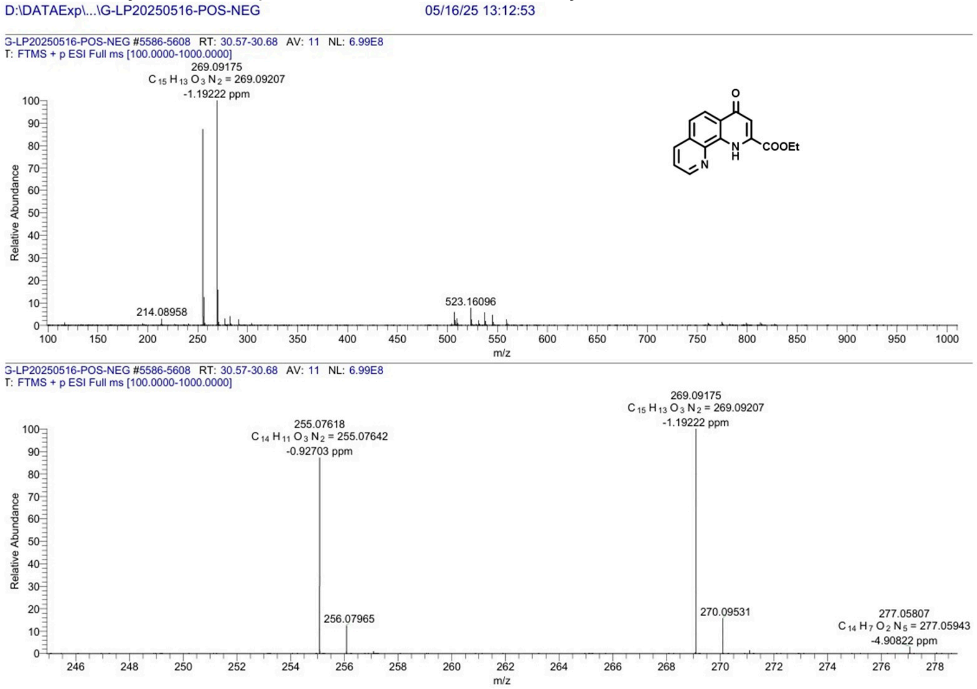

Figure S80. HR-MS spectrum of **8**

*Ethyl 6-oxo-6,9-dihydro-1H-pyrrolo[3,2-h]quinoline-8-carboxylate (**9**)*

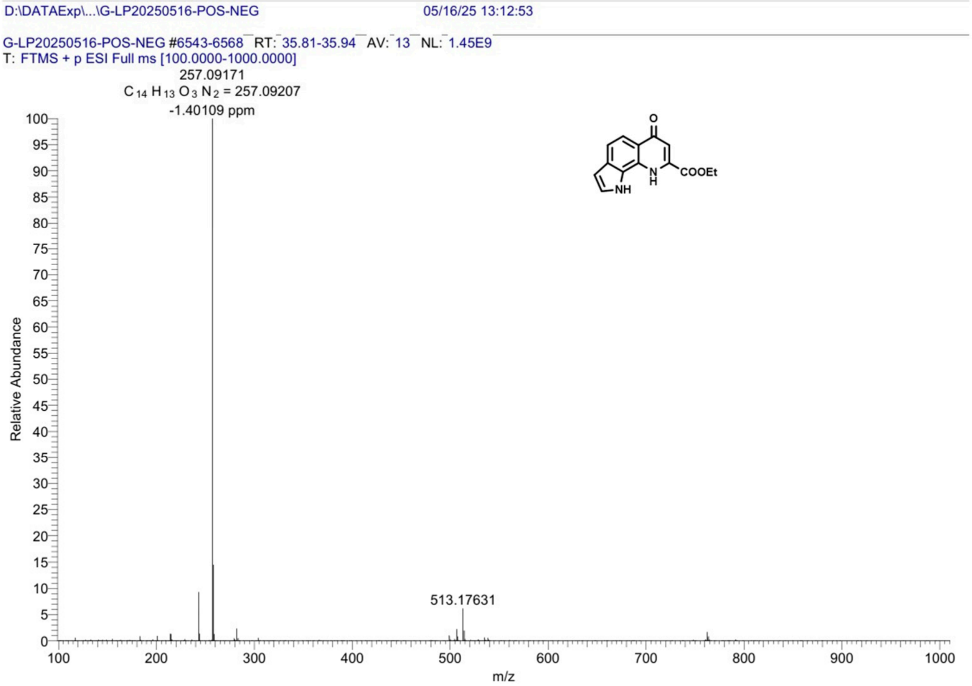

Figure S81. HR-MS spectrum of **9**

*3-(Morpholinomethyl)-4-oxo-1,4-dihydrobenzo[h]quinoline-2-carboxylic acid (**10a**)*

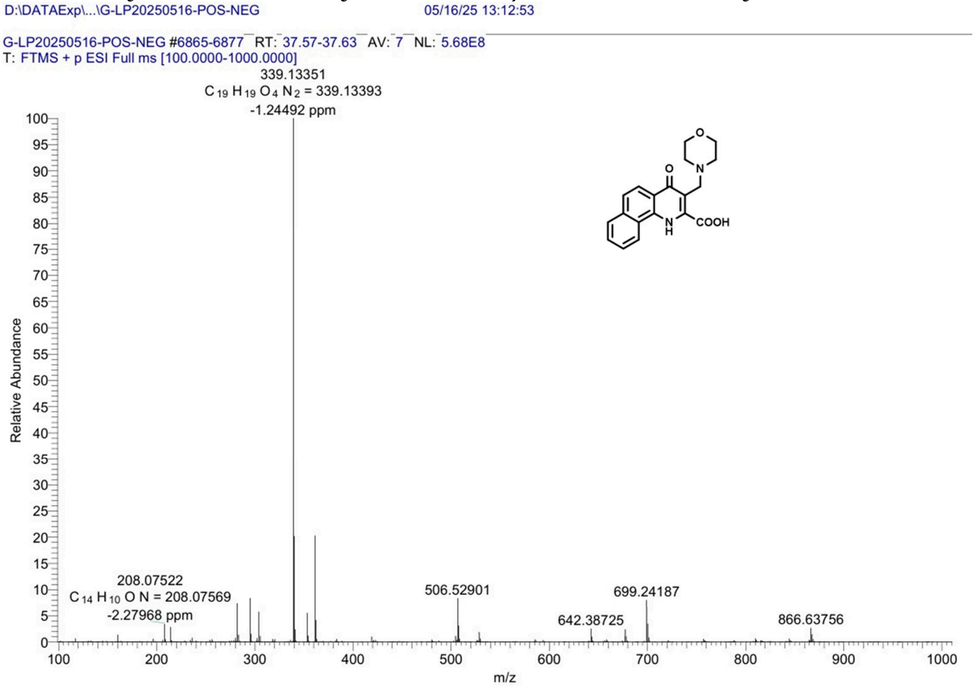

Figure S82. HR-MS spectrum of **10a**

*4-Oxo-3-(piperidin-1-ylmethyl)-1,4-dihydrobenzo[h]quinoline-2-carboxylic acid (**10b**)*

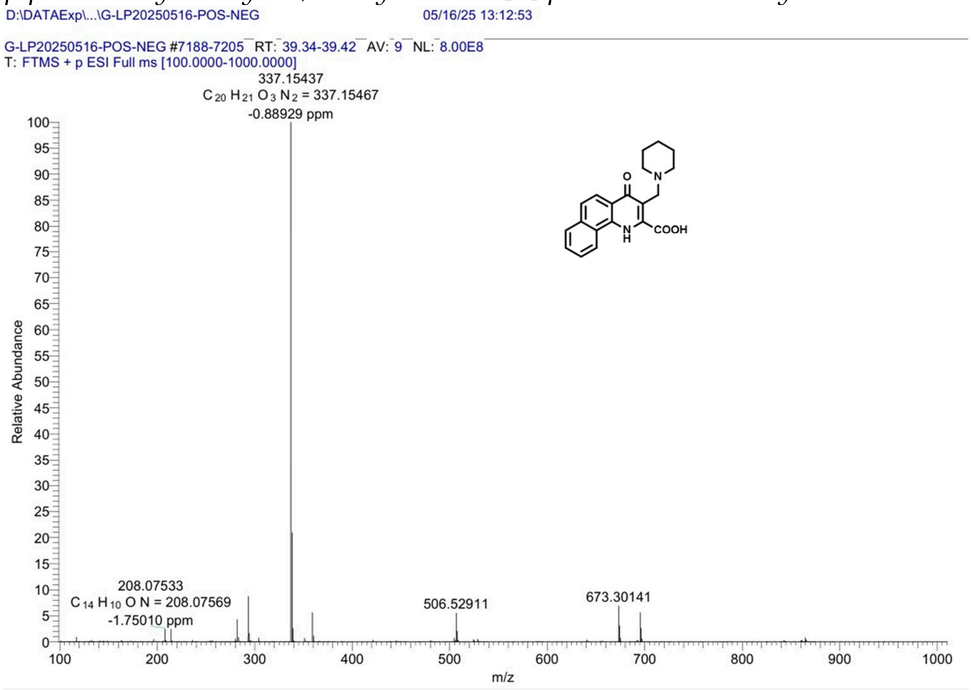

Figure S83. HR-MS spectrum of **10b**

*4-Oxo-3-(pyrrolidin-1-ylmethyl)-1,4-dihydrobenzo[h]quinoline-2-carboxylic acid (**10c**)*

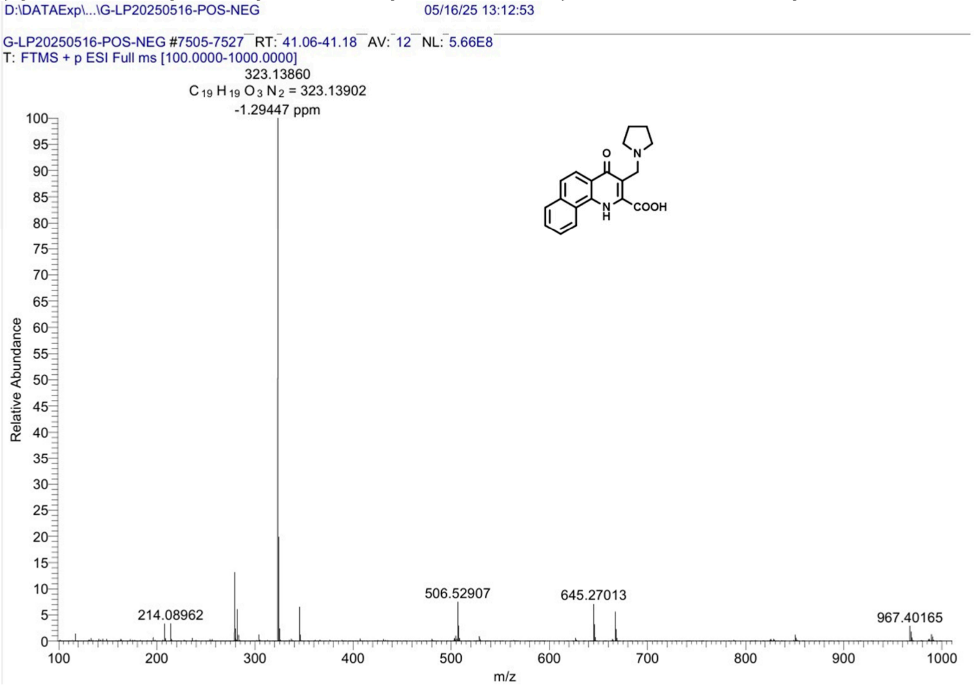

Figure S84. HR-MS spectrum of **10c**

*3-((4-Methylpiperazin-1-yl)methyl)-4-oxo-1,4-dihydrobenzo[h]quinoline-2-carboxylic acid (***10d***)*

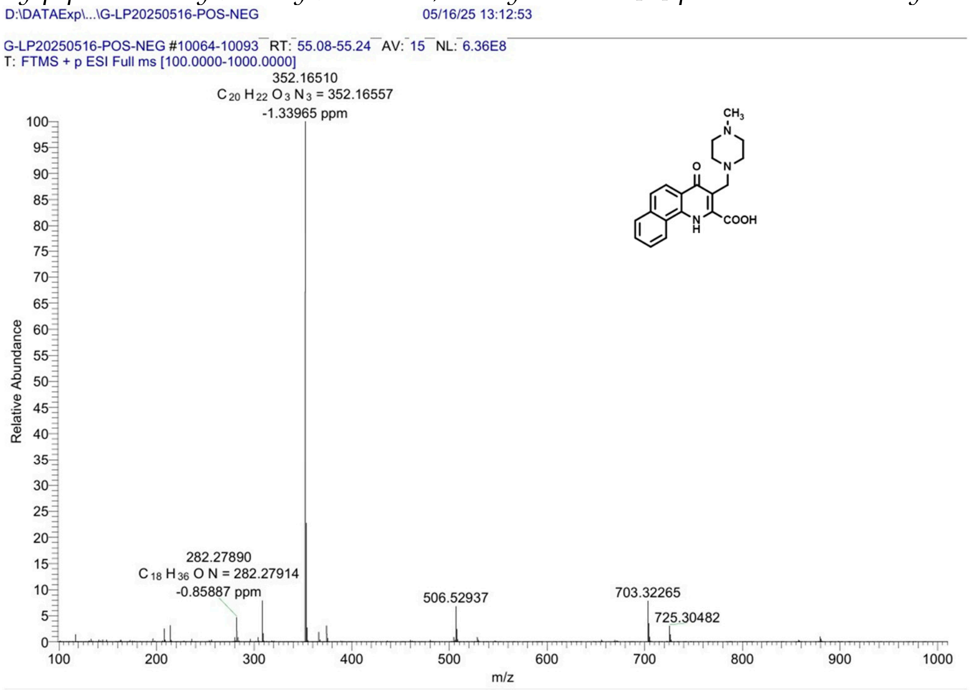

Figure S85. HR-MS spectrum of **10d**

*3-(Morpholinomethyl)-4-oxo-1,4-dihydro-1,10-phenanthroline-2-carboxylic acid (**11a**)*

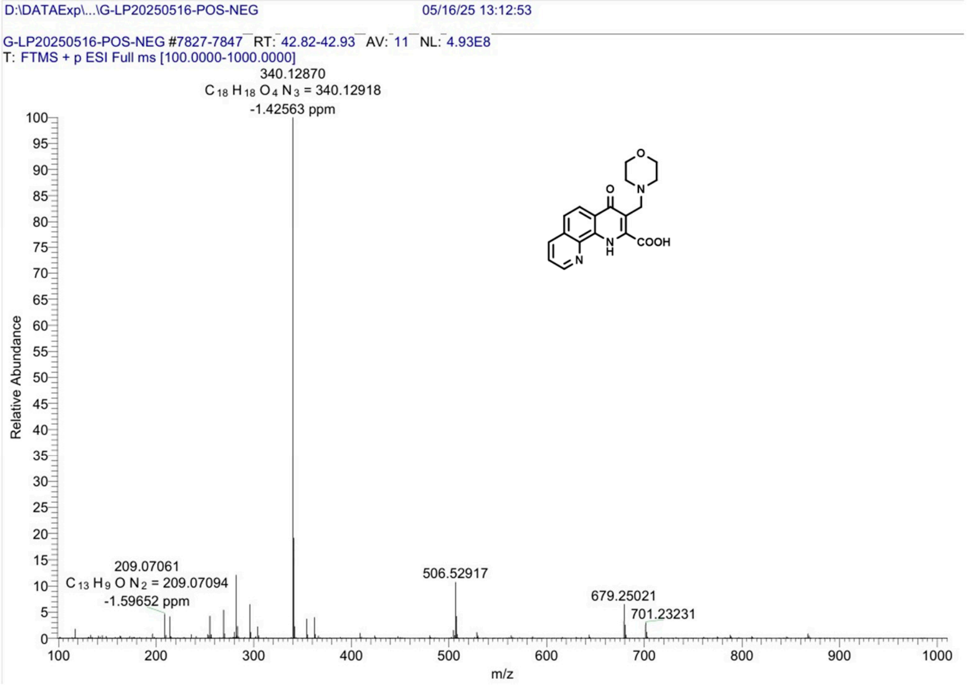

Figure S86. HR-MS spectrum of **11a**

*4-Oxo-3-(piperidin-1-ylmethyl)-1,4-dihydro-1,10-phenanthroline-2-carboxylic acid (**11b**)*

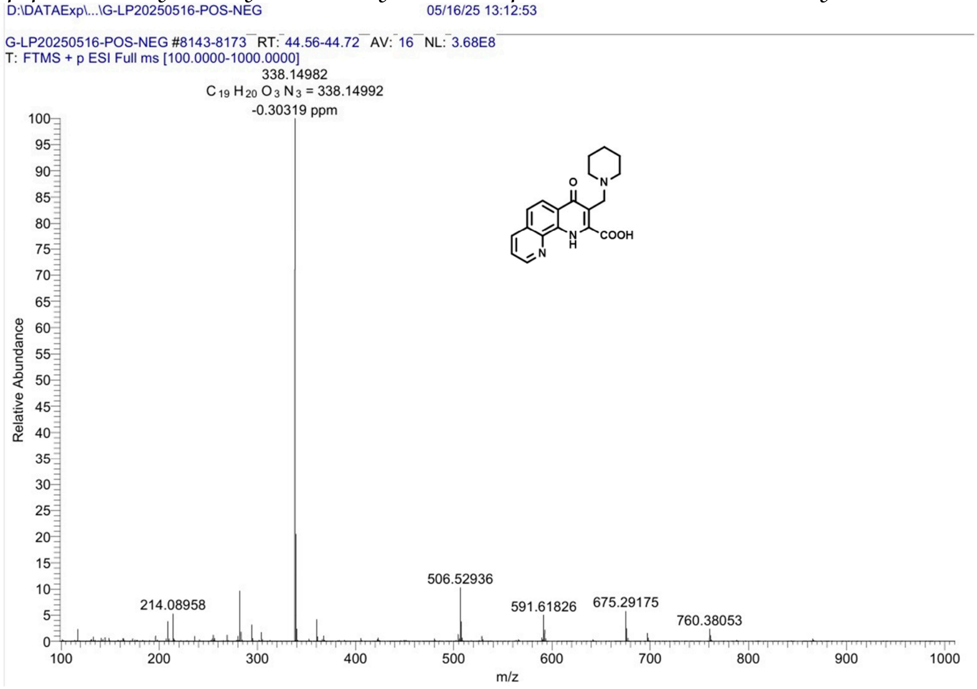

Figure S87. HR-MS spectrum of **11b**

*4-Oxo-3-(pyrrolidin-1-ylmethyl)-1,4-dihydro-1,10-phenanthroline-2-carboxylic acid (**11c**)*

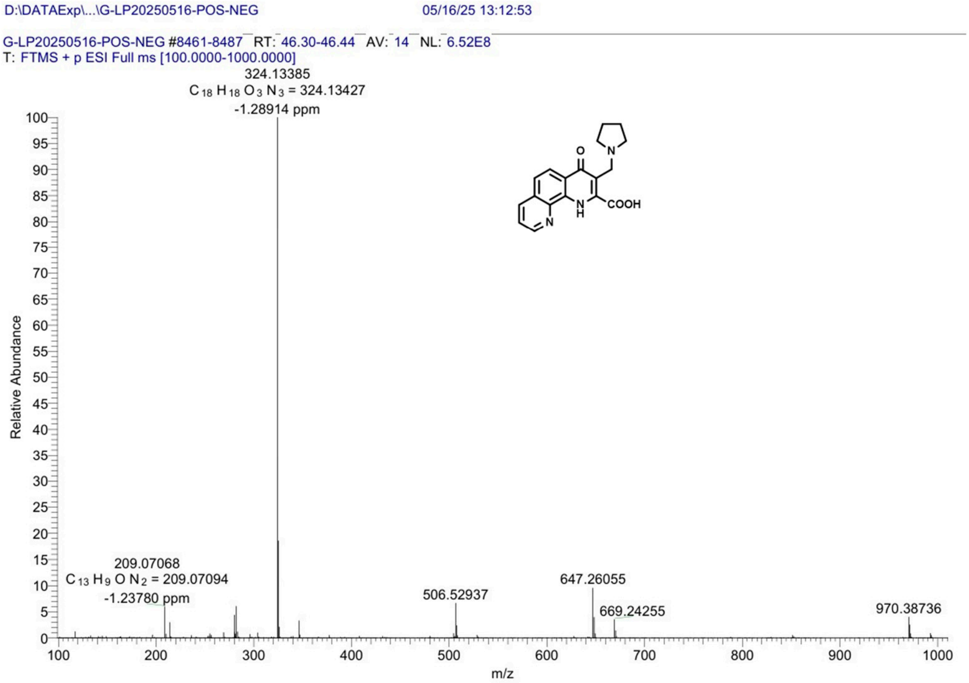

Figure S88. HR-MS spectrum of **11c**

*3-((4-Methylpiperazin-1-yl)methyl)-4-oxo-1,4-dihydro-1,10-phenanthroline-2-carboxylic acid (**11d**)*

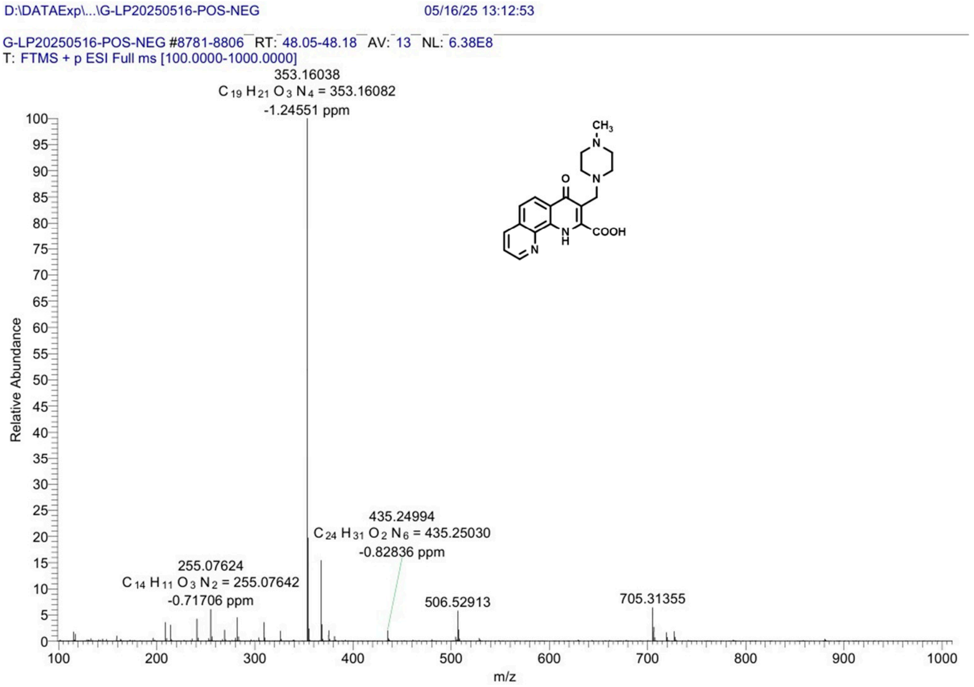

Figure S89. HR-MS spectrum of **11d**

*7-(Morpholinomethyl)-6-oxo-6,9-dihydro-1H-pyrrolo[3,2-h]quinoline-8-carboxylic acid (**12a**)*

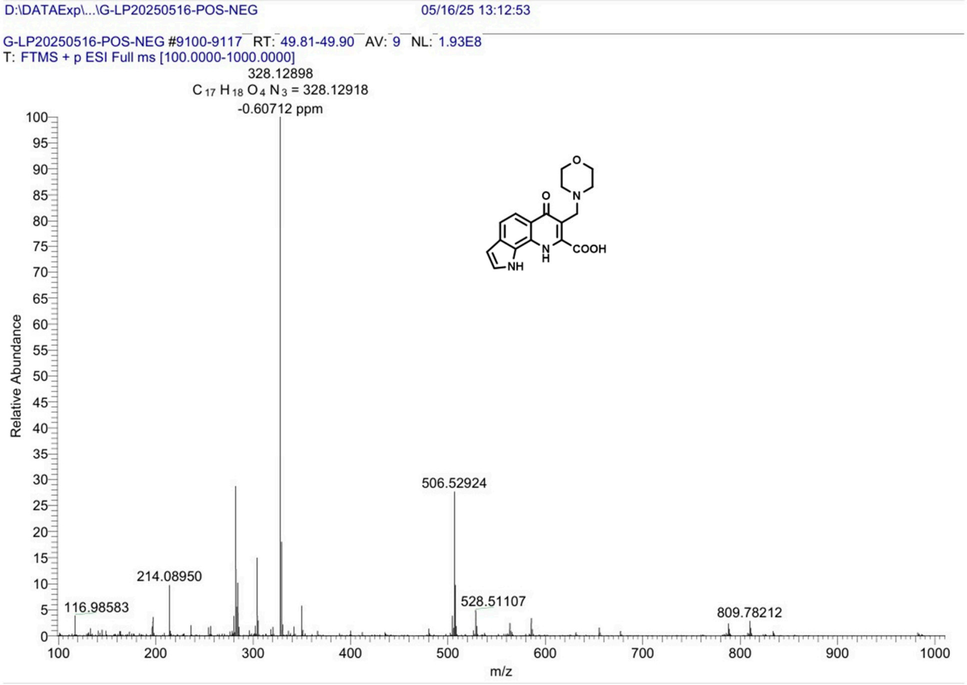

Figure S90. HR-MS spectrum of **12a**

*6-oxo-7-(piperidin-1-ylmethyl)-6,9-dihydro-1H-pyrrolo[3,2-h]quinoline-8-carboxylic acid (**12b**)*

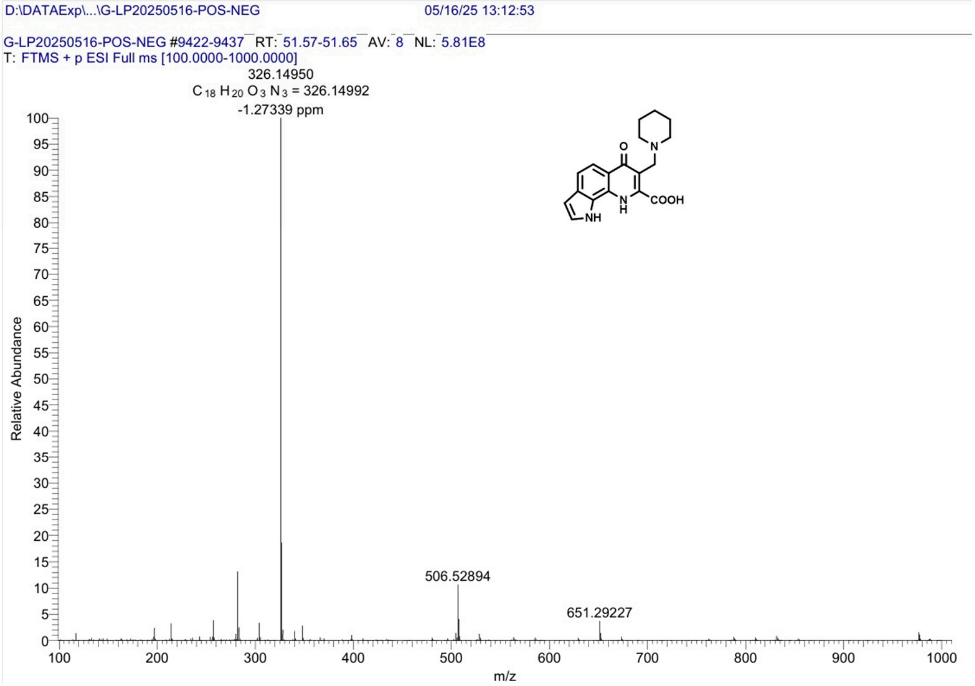

Figure S91. HR-MS spectrum of **12b**

*6-oxo-7-(pyrrolidin-1-ylmethyl)-6,9-dihydro-1H-pyrrolo[3,2-h]quinoline-8-carboxylic acid (**12c**)*

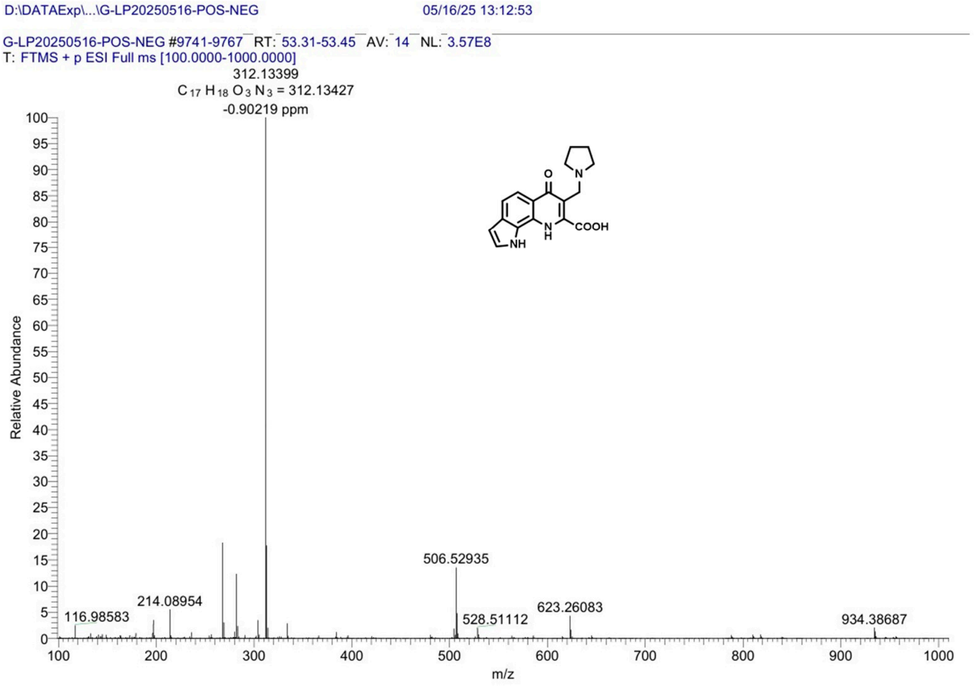

Figure S92. HR-MS spectrum of **12c**

*7-((4-methylpiperazin-1-yl)methyl)-6-oxo-6,9-dihydro-1H-pyrrolo[3,2-h]quinoline-8-carboxylic acid (**12d**)*

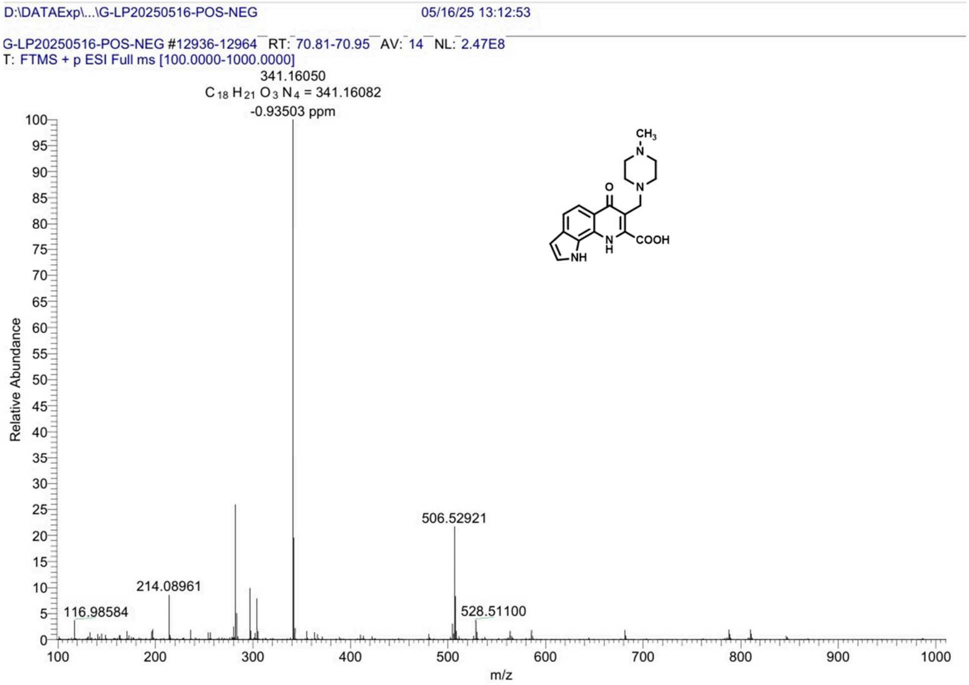

Figure S93. HR-MS spectrum of **12d**

*N-(2-(dimethylamino)ethyl)-4-oxo-1,4-dihydrobenzo[h]quinoline-2-carboxamide (**13**)*

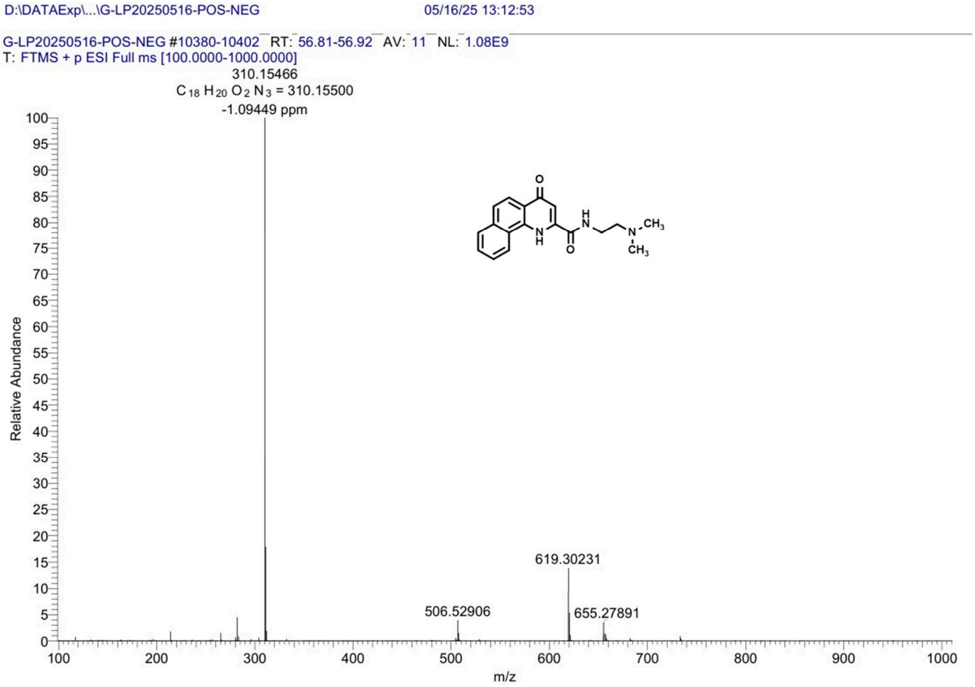

Figure S94. HR-MS spectrum of **13**

*N-(2-(dimethylamino)ethyl)-4-oxo-1,4-dihydro-1,10-phenanthroline-2-carboxamide (**14**)*

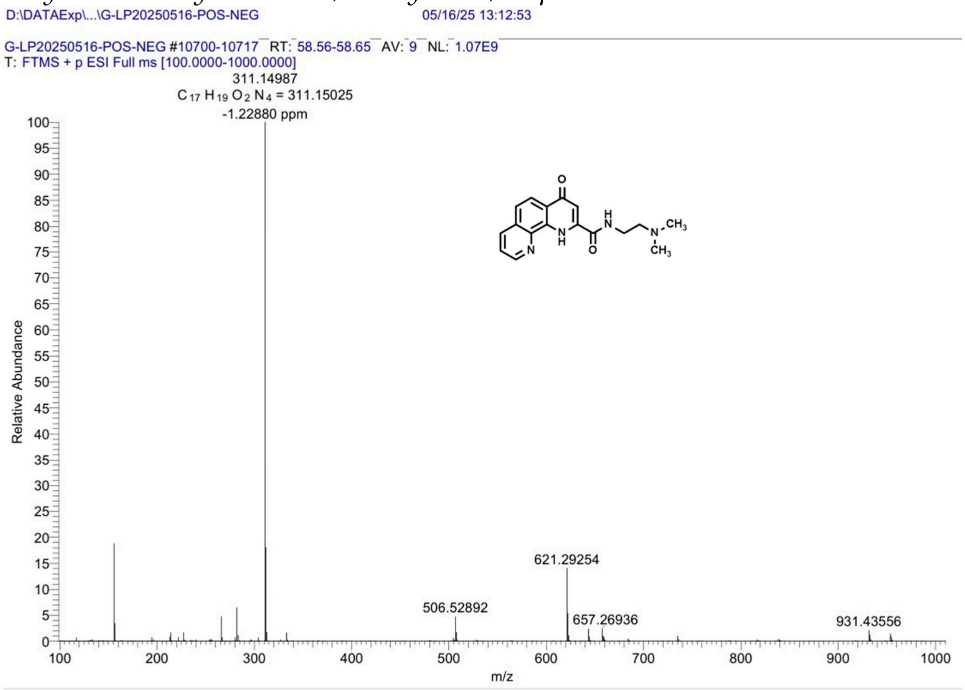

Figure S95. HR-MS spectrum of **14**

N-(2-(dimethylamino)ethyl)-6-oxo-6,9-dihydro-1H-pyrrolo[3,2-h]quinoline-8-carboxamide (**15**)

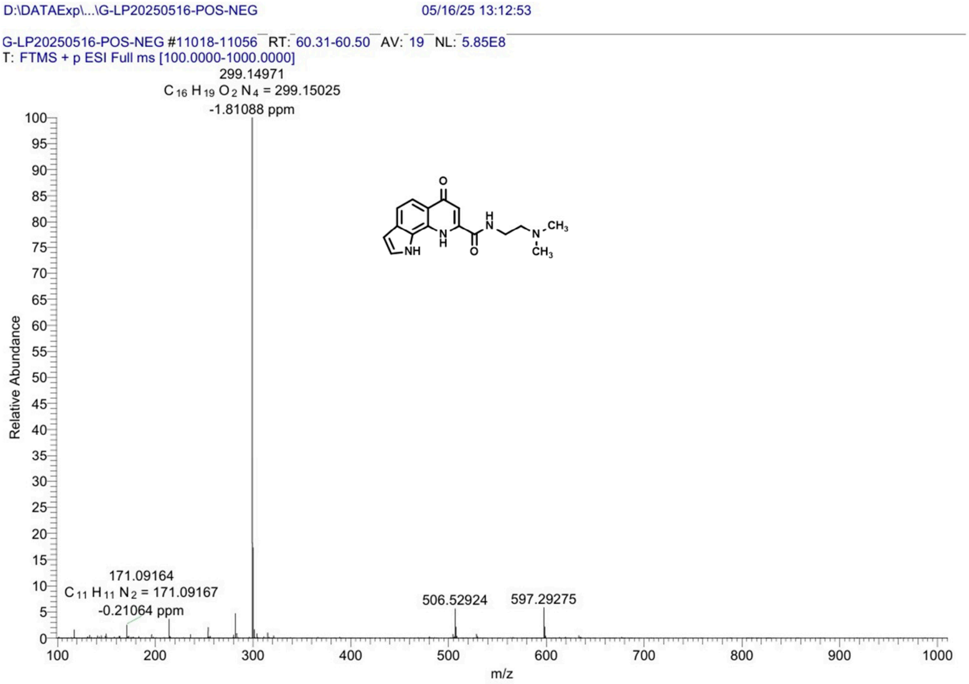

Figure S96. HR-MS spectrum of **15**

*N-(2-(dimethylamino)ethyl)-3-(morpholinomethyl)-4-oxo-1,4-dihydrobenzo[h]quinoline-2-carboxamide (***16a***)*

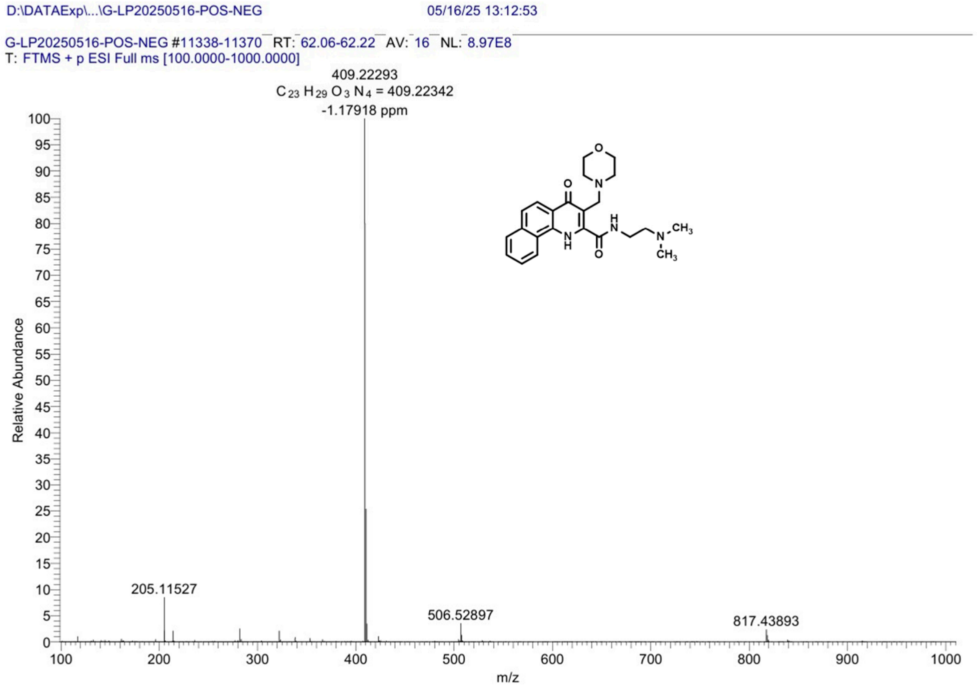

Figure S97. HR-MS spectrum of **16a**

*N-(2-(dimethylamino)ethyl)-4-oxo-3-(piperidin-1-ylmethyl)-1,4-dihydrobenzo[h]quinoline-2-carboxamide (**16b**)*

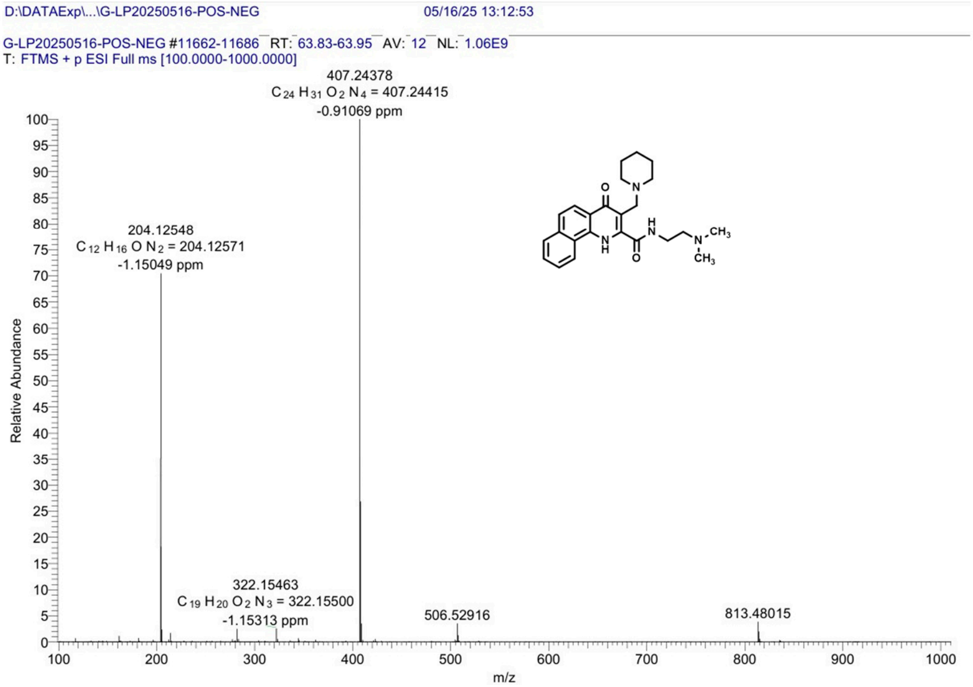

Figure S98. HR-MS spectrum of **16b**

N-(2-(dimethylamino)ethyl)-4-oxo-3-(pyrrolidin-1-ylmethyl)-1,4-dihydrobenzo[h]quinoline-2-carboxamide (**16c**)

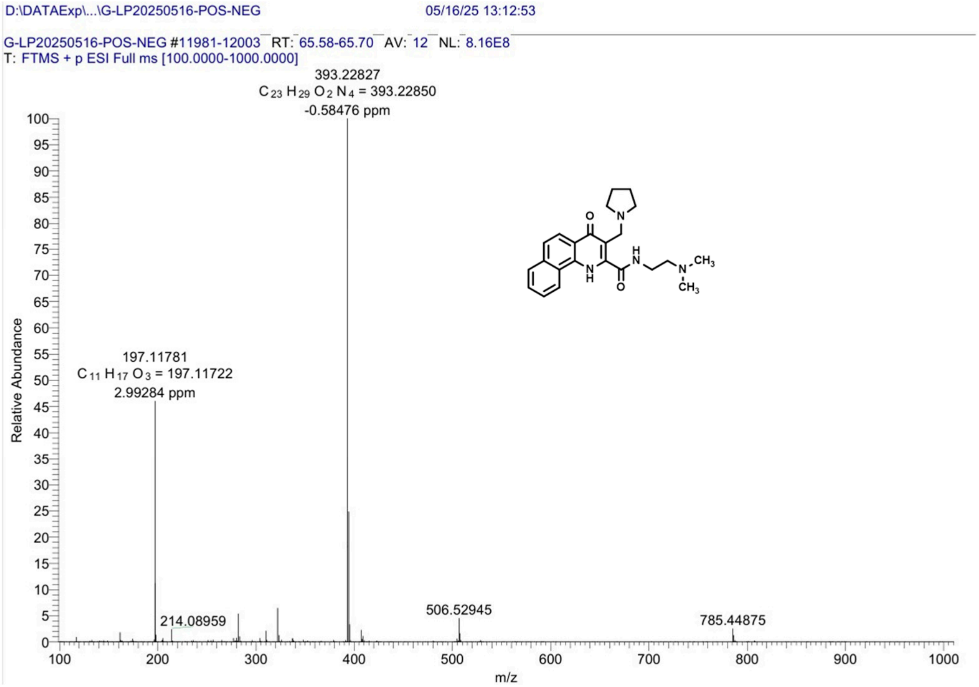

Figure S99. HR-MS spectrum of **16c**

*N-(2-(dimethylamino)ethyl)-3-((4-methylpiperazin-1-yl)methyl)-4-oxo-1,4-dihydrobenzo[h]quinoline-2-carboxamide (**16d**)*

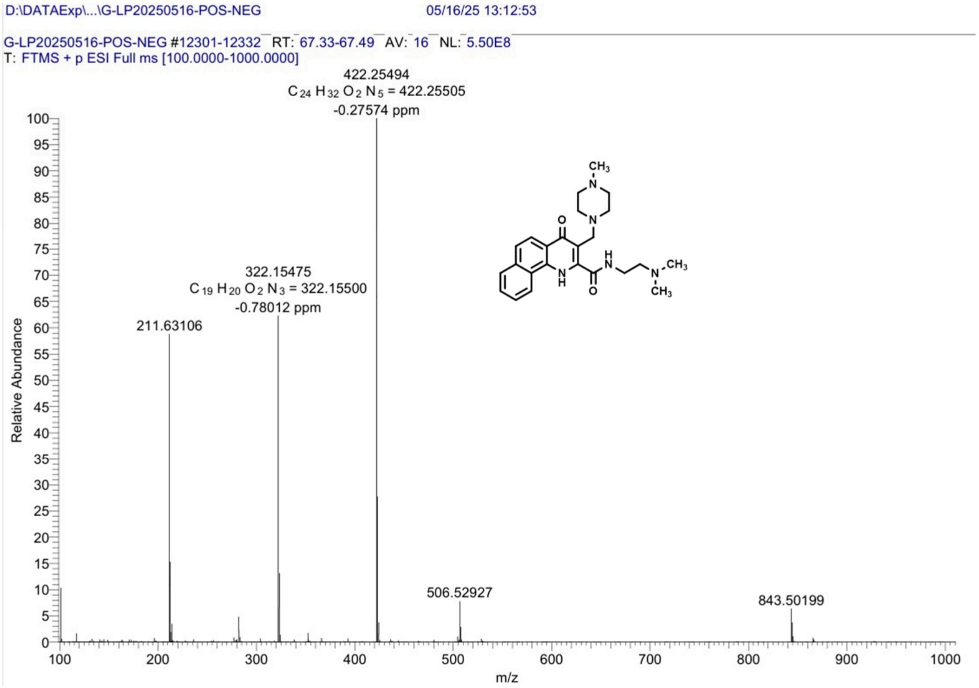

Figure S100. HR-MS spectrum of **16d**

*N-(2-(dimethylamino)ethyl)-3-(morpholinomethyl)-4-oxo-1,4-dihydro-1,10-phenanthroline-2-carboxamide (***17a***)*

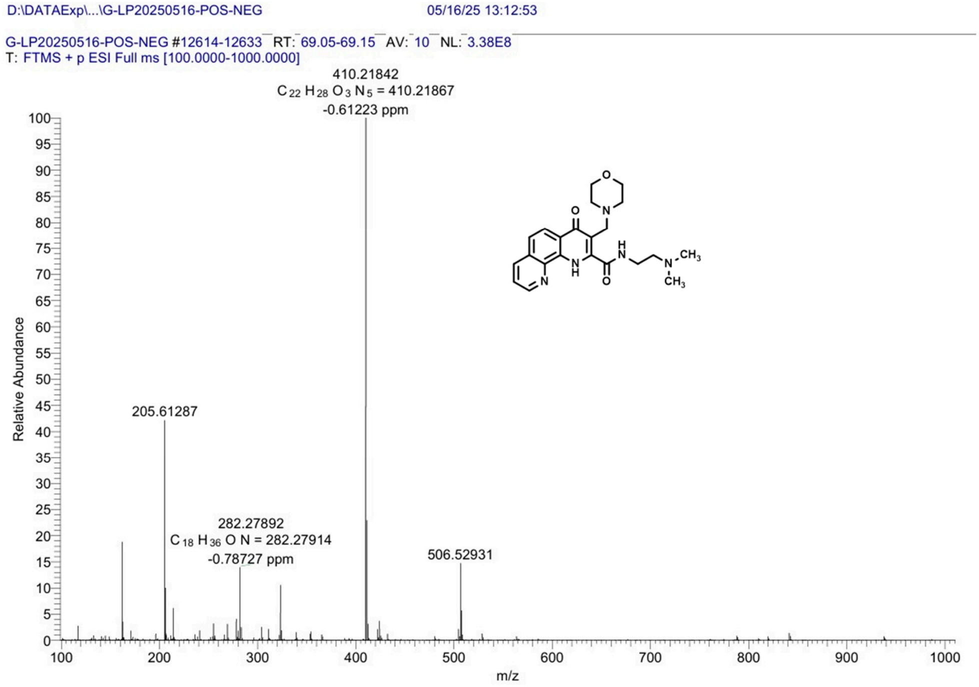

Figure S101. HR-MS spectrum of **17a**

*N-(2-(dimethylamino)ethyl)-4-oxo-3-(piperidin-1-ylmethyl)-1,4-dihydro-1,10-phenanthroline-2-carboxamide (**17b**)*

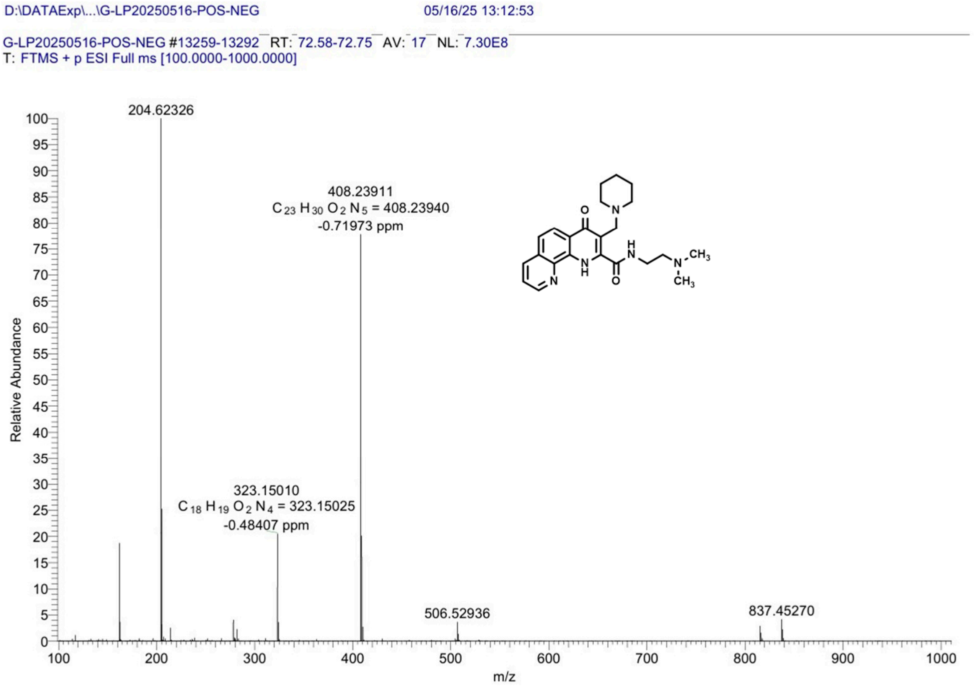

Figure S102. HR-MS spectrum of **17b**

*N-(2-(dimethylamino)ethyl)-4-oxo-3-(pyrrolidin-1-ylmethyl)-1,4-dihydro-1,10-phenanthroline-2-carboxamide (**17c**)*

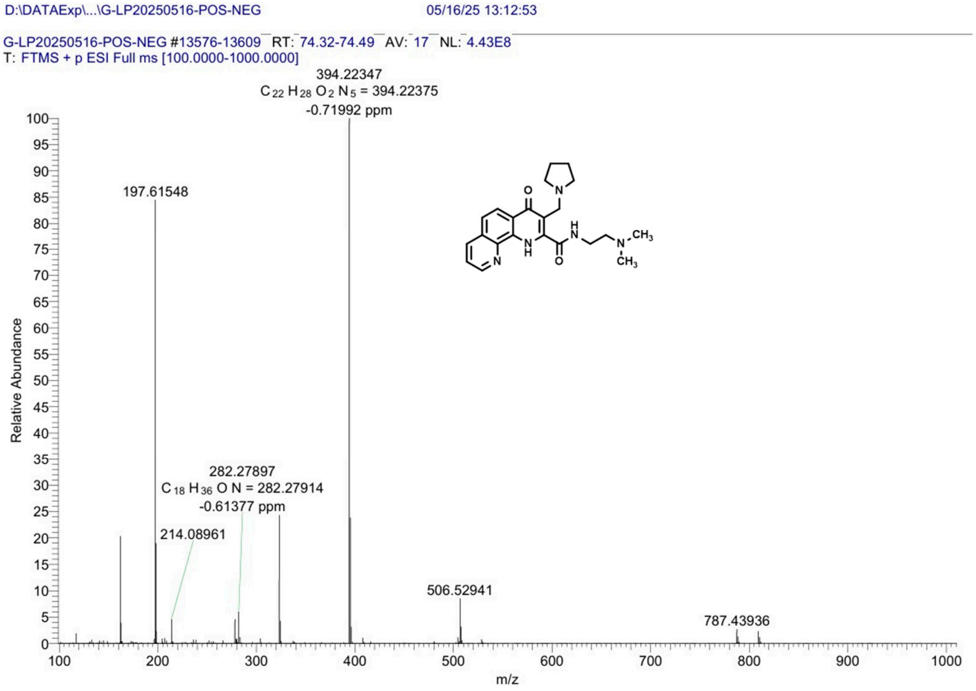

Figure S103. HR-MS spectrum of **17c**

*N-(2-(dimethylamino)ethyl)-3-((4-methylpiperazin-1-yl)methyl)-4-oxo-1,4-dihydro-1,10-phenanthroline-2-carboxamide (***17d***)*

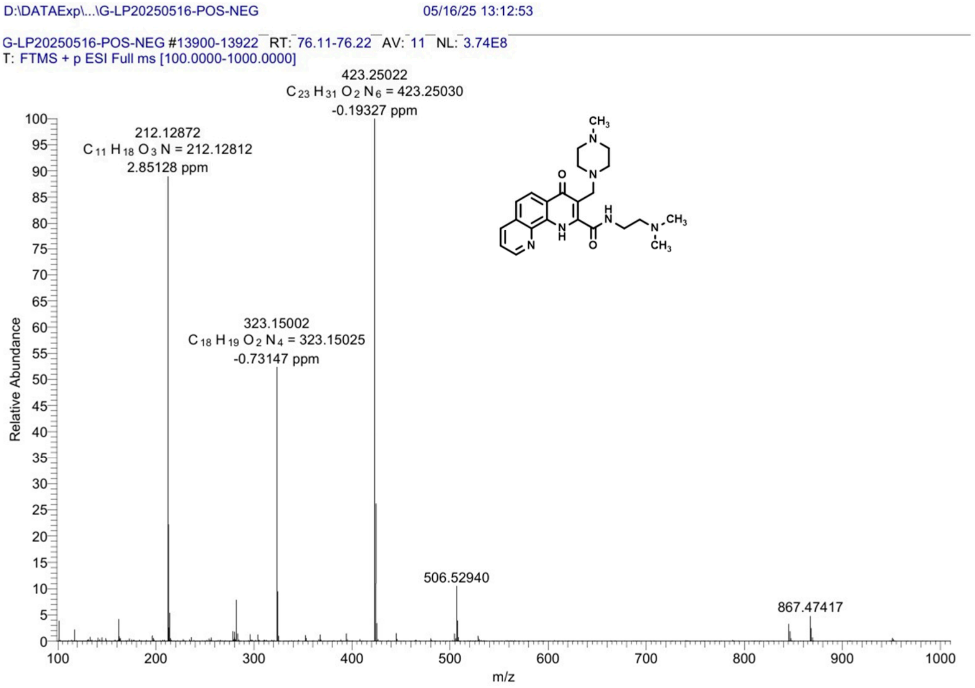

Figure S104. HR-MS spectrum of **17d**

*N-(2-(dimethylamino)ethyl)-7-(morpholinomethyl)-6-oxo-6,9-dihydro-1H-pyrrolo[3,2-h]quinoline-8-carboxamide (***18a***)*

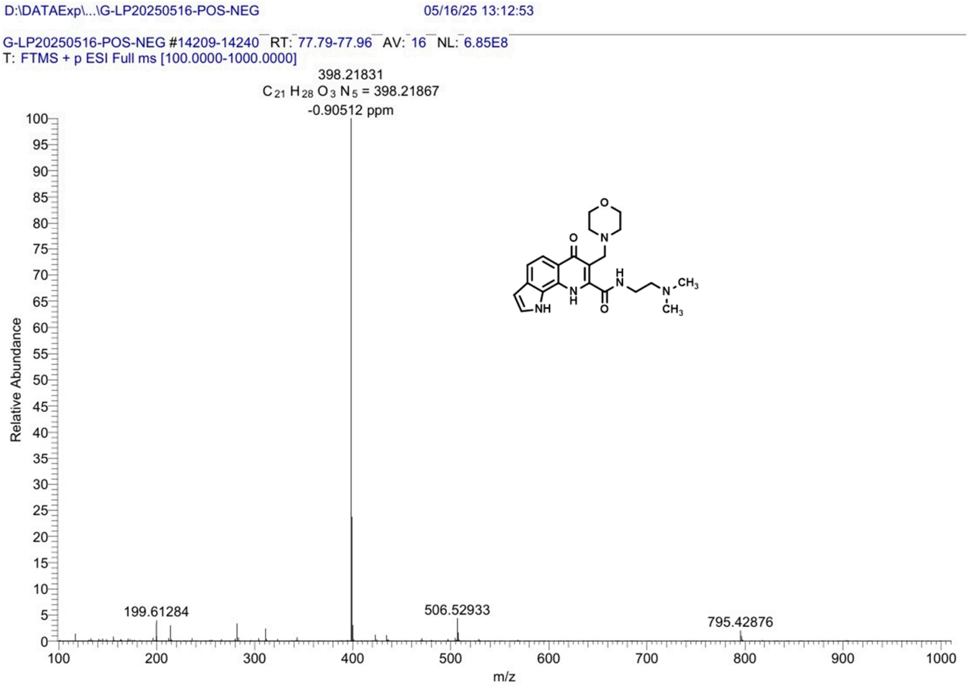

Figure S105. HR-MS spectrum of **18a**

*N-(2-(dimethylamino)ethyl)-6-oxo-7-(piperidin-1-ylmethyl)-6,9-dihydro-1H-pyrrolo[3,2-h]quinoline-8-carboxamide (**18b**)*

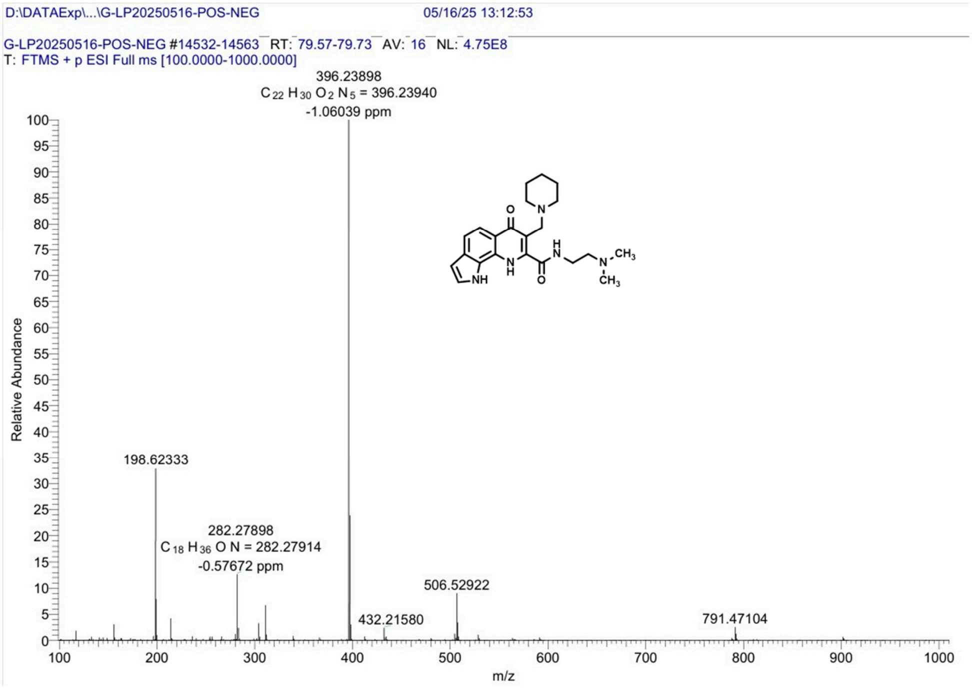

Figure S106. HR-MS spectrum of **18b**

*N-(2-(dimethylamino)ethyl)-6-oxo-7-(pyrrolidin-1-ylmethyl)-6,9-dihydro-1H-pyrrolo[3,2-h]quinoline-8-carboxamide (***18c***)*

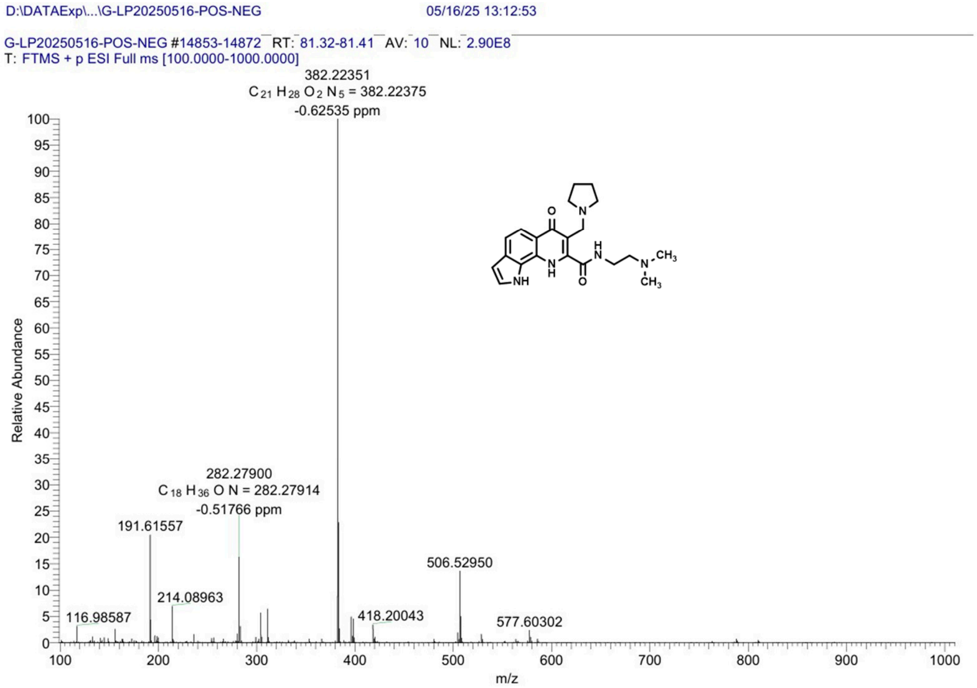

Figure S107. HR-MS spectrum of **18c**

N-(2-(dimethylamino)ethyl)-7-((4-methylpiperazin-1-yl)methyl)-6-oxo-6,9-dihydro-1H-pyrrolo[3,2-h]quinoline-8-carboxamide (**18d**)

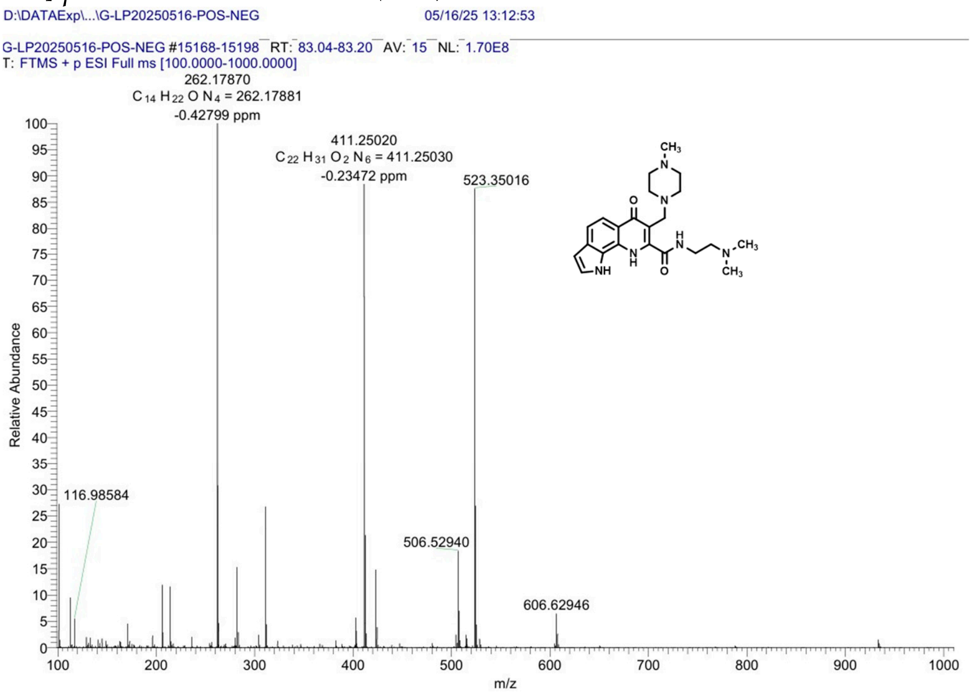

Figure S108. HR-MS spectrum of **18d**
